# Supplementary material for: Chirally locked and dynamic bis-perylene diimide macrocycles with multiple sources of chirality
Source: Commun Chem. 2026 Jan 23;9:102. doi: 10.1038/s42004-026-01904-z (PMC12932710; doi:10.1038/s42004-026-01904-z)
Supplement: Supplementary file 2 — Supplementary Information [file 42004_2026_1904_MOESM2_ESM.pdf]

Supplementary Information

Chirally locked and dynamic bis-perylene diimide macrocycles with multiple sources of chirality

Denis Hartmann,[a] Samuel E. Penty,[b] Robert Pal,[b] Timothy A. Barendt[a,*]

[a]University of Birmingham, School of Chemistry, Edgbaston Campus, Birmingham, B15 2TT, United Kingdom
[b]University of Durham, Department of Chemistry, Stockton Road, Durham, DH1 3LE, United Kingdom

Contents

1) Synthesis 2
2) Spectroscopy 13
3) Chiral HPLC 19
4) Host/Guest Chemistry 20
5) Crystallography 30
6) Computational Chemistry 32
7) NMR Spectra 39
8) Variable Temperature NMR 62
9) Supplementary References 75

1) Synthesis

General Synthesis

Reagents were purchased from commercial sources (Merck, Fisher Scientific, Acros Organics, Fluorochem and Alfa Aesar) and used without further purification. Solvents were used as supplied (analytical/HPLC-grade from Fisher or Sigma-Aldrich). If required, solvents were dried over molecular sieves (3 Å) overnight before use, and stored under N_2 atmosphere. Petroleum ether (PE) over a boiling point range of 40–60 °C was used. Eluent mixtures are reported in volume:volume or %vol. Column chromatography was carried out using Merck Silica Gel 60 Å, 230-400 mesh, 40-63 µm particle size. TLC was carried out on Merck silica gel 60 F254 Al plates. Preparative TLC was performed using 20 × 20 cm plates with 1 cm silica thickness. NMR spectroscopy measurements were recorded using a Bruker AVIII300, AVIII400, AV Neo 400 or AV Neo 500 instrument and peaks were referenced to the residual solvent peak. Electrospray Ionisation Mass Spectrometry (ESI-MS) measurements were carried out Waters Xevo G2-XS TOF Mass Spectrometer.

L-Valinol-Perylene Diimide-1,7-Dibromide 1

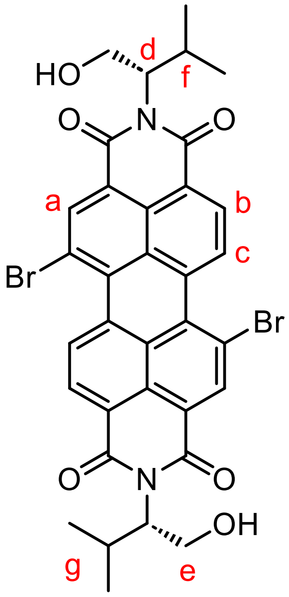

To a round-bottom flask under N2 atmosphere was added dibromo-perylene tetracarboxylic acid dianhydride (1 g, 1.82 mmol), L-valinol (375 mg, 3.64 mmol), 1,4-dioxane (20 mL) and dimethylacetamide (20 mL). The reaction mixture was stirred at 140 °C for 24 h. The mixture was then cooled, and the solvent reduced *in vacuo*. The resulting residue was purified by flash column chromatography (SiO_2, 50% EtOAc/PE_{40-60}) to yield a bright red solid as the title compound as a mixture of 1,7- and 1,6-brominated species (956 mg, 1.33 mmol, 73%).

$\mathbf{R_f}$ (SiO_2, 50% EtOAc/PE_{40-60}) 0.24.

^{1}H NMR (400 MHz, $CDCl_3$) δ/ppm 9.37 (d, *J* = 8.0 Hz, 2H, **b** or **c**), 8.85 (s, *J* = 6.6 Hz, 2H, **a**), 8.60 (d, *J* = 8.2 Hz, 2H, **b** or **c**), 5.01 (ddd, *J* = 10.9, 8.0, 3.0 Hz, 2H, **d**), 4.35 (dd, *J* = 12.3, 8.0 Hz, 2H, **e'**), 4.05 (dd, *J* = 12.3, 3.0 Hz, 2H, **e''**), 2.79 – 2.62 (m, 2H, **f**), 1.18 (d, *J* = 6.6 Hz, 6H, **g'**), 0.84 (d, *J* = 6.7 Hz, 6H, **g''**).

^{13}C NMR (101 MHz, $CDCl_3$) δ/ppm 164.27, 163.74, 138.58, 132.96, 132.86, 130.52, 129.11, 128.53, 127.16, 123.14, 122.76, 121.09, 62.58, 62.37, 26.42, 20.52, 20.05.

HRMS (ESI^+) found 721.0335, $[C_{34}H_{29}{}^{79}Br^{81}BrN_2O_6]^+$ requires 721.0372.

TBS-O-L-Valinol PDI Dibromide 2

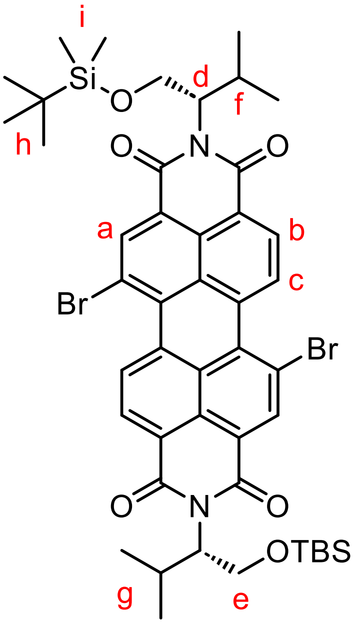

To a dry round-bottom flask under N_2 atmosphere was added **1** (630 mg, 0.718 mmol) as a mixture of 1,7- and 1,6-brominated species. To this was then added imidazole (298 mg, 4.38 mmol) and TBDMSCl (660 mg, 4.38 mmol) and DMF (18 mL). The reaction was stirred at r.t. for 4 h, after which TLC showed complete conversion to the di-protected material. The solvent was removed *in vacuo* and the resulting residue purified by flash column chromatography (SiO_2, 5-10% EtOAc/PE_{40-60}). To separate the 1,7- from the 1,6-isomer, the resulting residue was the subjected to HPLC. The isomers were separated on a COSMOSIL Buckyprep column (28 mm x 250 mm) using a gradient of 20-38% DCM in Hexane over 20 minutes, with the first, and major species being the 1,7-isomer, which was collected (590 mg, 0.622 mmol, 71%).

$\mathbf{R}_f$ (SiO_2, 10% EtOAc/PE_{40-60}) 0.33.

^{1}H NMR (400 MHz, $CDCl_3$) δ/ppm 9.50 (d, *J* = 8.2 Hz, 2H, **b**), 8.90 (d, *J* = 22.6 Hz, 2H, **a**), 8.67 (dd, *J* = 22.5, 8.2 Hz, 2H, **c**), 4.99 (td, *J* = 10.0, 5.0 Hz, 2H, **d**), 4.32 (t, *J* = 9.9 Hz, 2H, **e'**), 4.05 (dd, *J* = 10.2, 5.2 Hz, 2H, **e''**), 2.64 (dp, *J* = 10.5, 6.6 Hz, 2H, **f**), 1.12 (d, *J* = 6.6 Hz, 6H, **g'**), 0.88 (d, *J* = 6.7 Hz, 6H, **g''**), 0.67 (s, 18H, **h**), -0.02 (s, 6H, **i'**), -0.11 (s, 6H, **i''**).

^{13}C NMR (101 MHz, $CDCl_3$) δ/ppm 164.34, 163.87, 163.22, 162.67, 138.47, 137.73, 133.01, 132.84, 130.51, 129.70, 129.39, 128.61, 127.26, 123.81, 123.39, 123.26, 122.83, 120.85, 62.47, 61.50, 27.52, 25.76, 20.91, 20.38, 18.05, -5.30, -5.42.

HRMS (ESI$^+$) found 947.2077, $[C_{46}H_{57}{}^{79}Br_2N_2O_6Si_2]^+$ requires 947.2117.

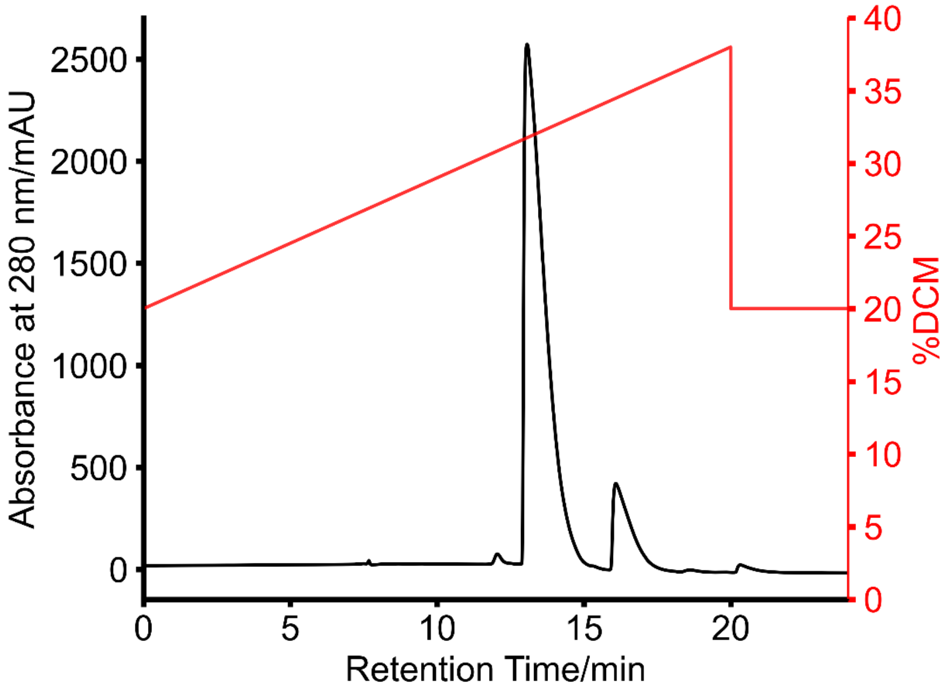

Supplementary Figure 1-1: HPLC purification of 1,7-isomer of **2** on a Cosmosil Buckyprep column using a gradient of 20-38% CH_2Cl_2 in Hexane.

TBS-O-bis(phenyl)-L-Valinol PDI 3

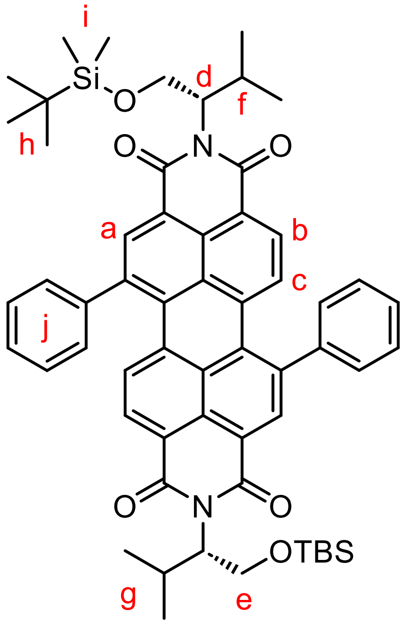

1,7-dibromo-PDI **2** (1 g, 1.06 mmol) and phenyl boronic acid (886 mg, 6.33 mmol, 6 eq.) were dissolved in PhMe (30 mL). To this was added EtOH (10 mL) and a solution of K_2CO_3 in H_2O (2 M, 5 mL). The resulting solution was degassed with N_2 before addition of $Pd(PPh_3)_2Cl_2$ (94 mg, 7 mol%). The reaction was heated to 70 °C and left stirring overnight. The solution was then cooled and the majority of the solvent removed *in vacuo*, before resuspension in CH_2Cl_2 (50 mL). The solution was then washed with H_2O (2x 20 mL) and brine (20 mL), dried ($MgSO_4$) and the solvent removed. The residue was purified by flash column chromatography (SiO_2, 5% EtOAc/PE) to yield the title compound as a red solid (1 g, 1.06 mmol, 100%)

^{1}H NMR (400 MHz, $CDCl_3$) δ/ppm 8.61 (d, *J* = 21.7 Hz, 2H, **a**), 8.12 (dd, *J* = 23.4, 8.1 Hz, 2H, **b**), 7.83 (d, *J* = 8.2 Hz, 2H, **c**), 7.62 – 7.44 (m, 10H, **j**), 4.98 (dp, *J* = 10.3, 5.1 Hz, 2H, **d**), 4.30 (t, *J* = 9.7 Hz, 2H, **e'**), 4.05 (dd, *J* = 10.2, 5.2 Hz, 2H, **e''**), 2.64 (tp, *J* = 12.3, 6.5 Hz, 2H, **f**), 1.11 (d, *J* = 6.7 Hz, 6H, **g'**), 0.88 (d, *J* = 6.7 Hz, 6H, **g'**), 0.68 (d, *J* = 3.9 Hz, 18H, **h**), -0.03 (s, 6H, **i'**), -0.11 (s, 6H, **i''**).

^{13}C NMR (101 MHz, $CDCl_3$) δ/ppm 164.95, 163.88, 142.39, 141.13, 135.80, 135.01, 134.92, 132.63, 130.45, 130.25, 130.01, 129.32, 129.23, 128.73, 127.99, 122.93, 122.61, 122.38, 122.04, 62.24, 61.80, 27.70, 25.80, 20.96, 20.43, -5.29, -5.44.

HRMS (ESI$^+$) found 943.4554, $[C_{58}H_{67}N_2O_6Si_2]^+$ requires 943.4532.

OH-bis(phenyl)-L-Valinol PDI 4

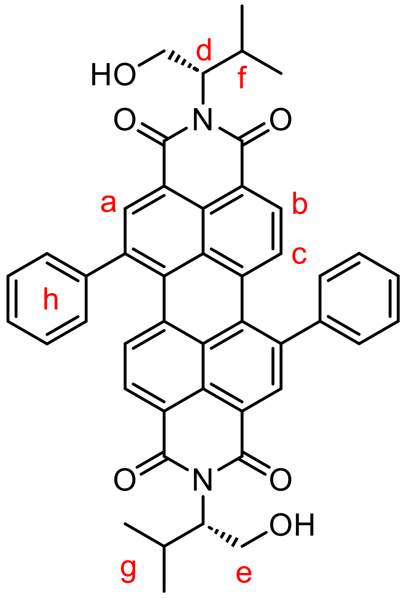

In a round-bottom flask under nitrogen, TBS-protected, Ph-substituted PDI **3** (875 mg, 0.928 mmol) were dissolved in Et_2O (10 mL). To this was added HCl in Et_2O (2 M, 10 mL), and the reaction left stirring until TLC indicated full conversion. The reaction mixture was diluted with CH_2Cl_2 (50 mL) and washed with H_2O (2 x 20 mL) and brine (10 mL), dried ($MgSO_4$) and reduced *in vacuo*. The residue was then purified using flash column chromatography (SiO_2, 5-50% EtOAc/PE) to yield a dark purple solid as the desired product. (532 mg, 0.745 mmol, 80%).

^{1}H NMR (400 MHz, TCE-d_2, 373 K) δ/ppm 8.64 (s, 2H, **a**), 8.17 (d, *J* = 8.2 Hz, 2H, **b** or **c**), 7.87 (d, *J* = 8.2 Hz, 2H, **b** or **c**), 7.58 (pd, *J* = 4.9, 2.6 Hz, 10H, **h**), 5.03 (ddd, *J* = 10.7, 7.7, 3.2 Hz, 2H, **d**), 4.33 (dd, *J* = 12.0, 7.7 Hz, 2H, **e'**), 4.06 (dd, *J* = 12.0, 3.3 Hz, 2H, **e''**), 2.77 (dp, *J* = 10.3, 6.6 Hz, 2H, **f**), 1.21 (d, *J* = 6.6 Hz, 6H, **g'**), 0.93 (d, *J* = 6.7 Hz, 6H, **g''**).

^{13}C NMR (101 MHz, TCE-d_2,) δ/ppm 164.54, 141.51, 141.10, 135.79, 134.42, 132.14, 130.20, 129.66, 128.78, 128.63, 127.31, 121.74, 121.36, 62.18, 61.78, 26.06, 20.35, 19.91.

HRMS (ESI$^+$) found 715.2811, $[C_{46}H_{39}N_2O_6]^+$ requires 715.2803.

Bis(phenyl)-L-Valinol PDI Macrocycle 5

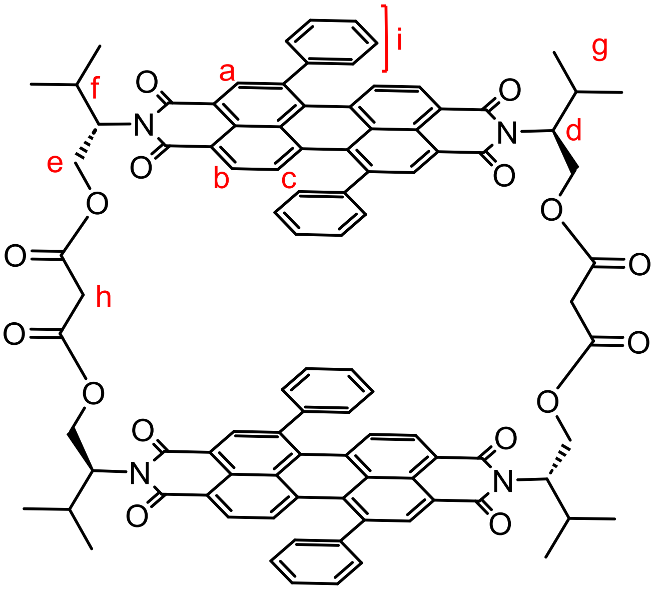

To a 1L round-bottom flask under nitrogen, Ph-substituted diol-PDI **4** (400 mg, 0.560 mmol) were dissolved in dry CH_2Cl_2 (500 mL). To this dry pyridine (1.11 g, 14 mmol, 25 eq.) were added. A solution of malonyl chloride (55 µL, 0.560 mmol) in dry CH_2Cl_2 (5 mL) was then added dropwise via syringe pump over ~8 hours and the reaction left stirring for 16 h. The solvent was then removed under vacuum and the residue purified by flash column chromatography (SiO_2, 5-40% EtOAc/Hexane). The fractions containing the macrocycle by mass spectrometry were combined and purified by preparative TLC (3% EtOAc/Hexane) to yield the title compound (10 mg, 6.4 µmol, 3%).

^{1}H NMR (400 MHz, 373 K, TCE-*d*$_2$) δ/ppm 8.56 (s, 4H, **a**), 7.98 (s, 4H), 7.76 (s, 4H), 7.57 (s, 20H, **i**), 4.99 (s, 4H, **d**), 4.77 (s, 4H, **e'**), 4.68 (s, 4H, **e''**), 3.18 (s, 4H, **h**), 2.70 (s, 4H, **f**), 1.35 (d, J = 6.8 Hz, 12H, **g'**), 1.19 (d, J = 6.6 Hz, 12H, **g''**).

^{13}C NMR (101 MHz, 373 K, TCE-*d*$_2$) δ/ppm 165.43, 163.60, 142.07, 140.89, 135.29, 134.70, 132.28, 130.03, 129.80, 128.99, 128.79, 128.49, 127.71, 124.37, 123.50, 122.09, 120.21, 64.58, 58.61, 40.95, 27.77, 20.24, 19.69.

HRMS (ESI$^+$) found 1587.5122, $[C_{98}H_{76}N_4O_{16}Na]^+$ requires 1587.5148.

TBS-O-bis(terphenyl)-L-Valinol PDI 6

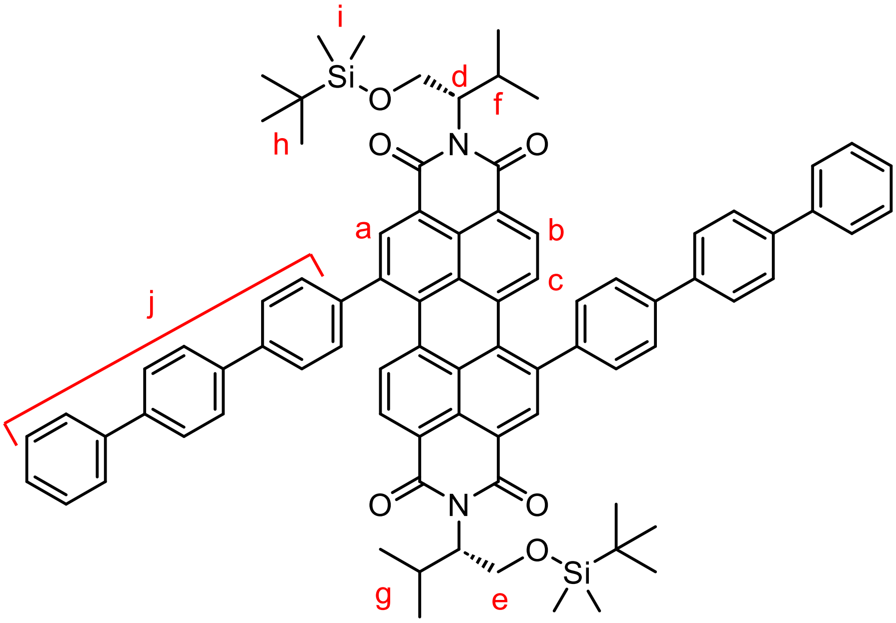

1,7-dibromo-PDI **2** (500 mg, 0.527 mmol) and 4(biphenyl)phenyl boronic acid (867 mg, 3.16 mmol, 6 eq.) were dissolved in PhMe (15 mL). To this was added EtOH (4.6 mL) and a solution of K_2CO_3 in H_2O (2 M, 2.5 mL). The resulting solution was degassed with N_2 before addition of $Pd(PPh_3)_2Cl_2$ (48 mg, 13 mol%). The reaction was heated to 70 °C and left stirring for 4 hours. The solution was then cooled and the majority of the solvent removed *in vacuo*, before resuspension in CH_2Cl_2 (50 mL). The solution was then washed with H_2O (2x 20 mL) and brine (20 mL), dried ($MgSO_4$) and the solvent removed. The residue was purified by flash column chromatography (SiO_2, 0-10% EtOAc/Hexane) to yield the title compound as a purple solid (655 mg, 0.525 mmol, 99%)

^{1}H NMR (400 MHz, $CDCl_3$) δ/ppm 8.68 (d, *J* = 21.8 Hz, 2H, **a**), 8.17 (dd, *J* = 24.7, 8.2 Hz, 2H, **b** or **c**), 8.00 (d, *J* = 7.7 Hz, 2H, **b** or **c**), 7.88 – 7.32 (m, 26H, **j**), 5.09 – 4.87 (m, 2H, **d**), 4.31 (t, *J* = 9.8 Hz, 2H, **e'**), 4.06 (dd, *J* = 10.3, 5.2 Hz, 2H, **e''**), 2.73 – 2.57 (m, 2H, **f**), 1.11 (d, *J* = 6.6 Hz, 6H, **g'**), 0.89 (d, *J* = 6.7 Hz, 6H, **g''**), 0.79 – 0.60 (m, 18H), -0.02 (s, 6H), -0.09 (s, 6H).

^{13}C NMR (101 MHz, $CDCl_3$) δ/ppm 164.97, 163.85, 141.34, 141.07, 140.85, 140.74, 139.06, 135.84, 134.97, 132.70, 130.47, 130.10, 129.84, 129.44, 129.03, 128.74, 128.42, 128.08, 127.84, 127.64, 127.25, 123.03, 122.70, 122.48, 122.13, 115.86, 62.27, 61.84, 27.69, 25.84, 20.97, 20.44, -5.26, -5.40.

HRMS (ESI$^+$) found 1247.5745, $[C_{82}H_{83}N_2O_6Si]^+$ requires 1247.5784.

OH-bis(terphenyl)-L-Valinol PDI 7

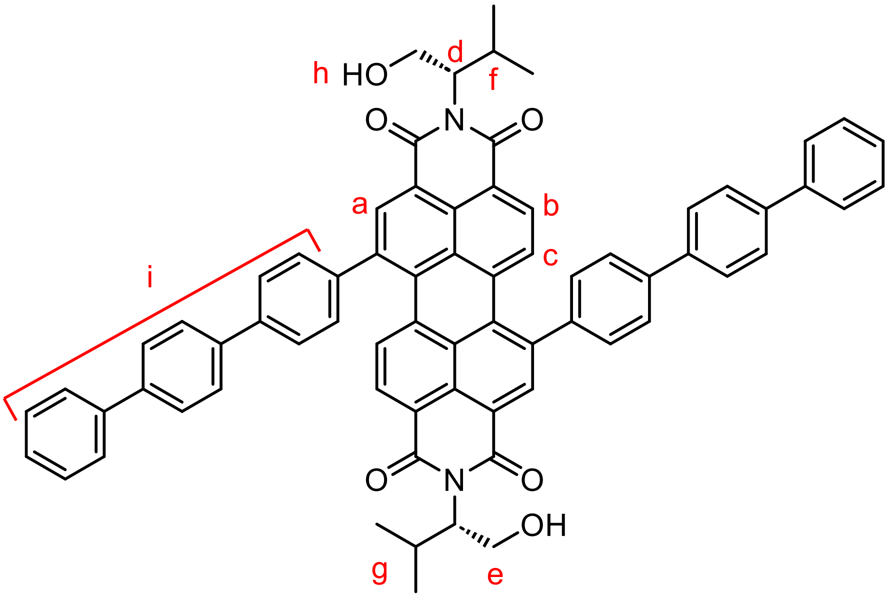

In a round-bottom flask under nitrogen, TBS-protected, terphenyl-substituted PDI **6** (100 mg, 0.080 mmol) were dissolved in Et_2O (5 mL). To this was added HCl in Et_2O (2 M, 5 mL), and the reaction left stirring for 24 hours. The reaction mixture was concentrated *in vacuo* and then diluted with CH_2Cl_2 (20 mL) and washed with H_2O (2 x 20 mL) and brine (10 mL), dried ($MgSO_4$) and reduced *in vacuo*. The residue was purified using flash column chromatography (SiO_2, 5-50% EtOAc/Hexane) to yield a dark purple solid as the desired product. (71 mg, 0.070 mmol, 88%).

^{1}H NMR (400 MHz, TCE-*d*$_2$, 373 K) δ/ppm 8.74 (s, 2H, **a**), 8.26 (d, *J* = 8.2 Hz, 2H, **c**), 8.08 (d, *J* = 8.2 Hz, 2H, **b**), 8.01 – 7.33 (m, 26H, **i**), 5.05 (ddd, *J* = 10.7, 7.5, 3.2 Hz, 2H, **d**), 4.34 (dd, *J* = 12.0, 7.6 Hz, 2H, **e'**), 4.07 (dd, *J* = 12.0, 3.2 Hz, 2H, **e''**), 2.90 – 2.64 (m, 4H, **f** and **h**), 1.22 (d, *J* = 6.6 Hz, 6H, **g'**), 0.96 (d, *J* = 6.7 Hz, 6H, **g''**).

^{13}C NMR (101 MHz, TCE-*d*$_2$, 298 K) δ/ppm 164.66, 164.56, 140.70, 140.65, 140.58, 140.37, 140.20, 138.49, 134.78, 132.46, 130.34, 129.42, 128.85, 128.54, 127.53, 127.40, 126.96, 121.90, 121.39, 62.42, 61.67, 25.95, 20.31, 19.89.

HRMS (ESI$^+$) found 1019.4045, $[C_{70}H_{55}N_2O_6]^+$ requires 1019.4055.

Bis(terphenyl)-L-Valinol PDI Macrocycle 8

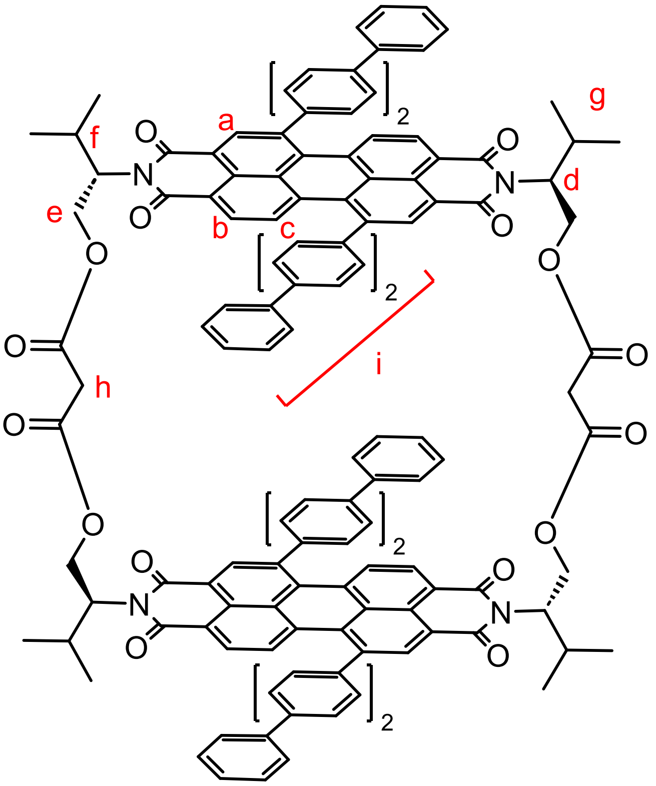

Terphenyl-substituted diol-PDI **7** (383 mg, 0.375 mmol) was dissolved in dry CH_2Cl_2 (500 mL). To this dry pyridine (800 μL, 9.8 mmol) was added. A solution of malonyl chloride (38 μL, 0.392 mmol) in dry CH_2Cl_2 (5 mL) was then added dropwise *via* syringe pump over ~8 hours and the reaction left stirring for 16 hours. The solvent was removed under vacuum and the residue purified by flash column chromatography (SiO_2, 5-50% EtOAc/Hexane). The fractions containing the macrocycle (5% EtOAc) were further purified by preparative TLC (1% EtOAc/ CH_2Cl_2) to yield the title compound as its MM isomer (5 mg, 2.3 μmol, 1%), PP isomer (5 mg, 2.3 μmol, 1%) and MP isomer (14 mg, 6.4 μmol, 2%).

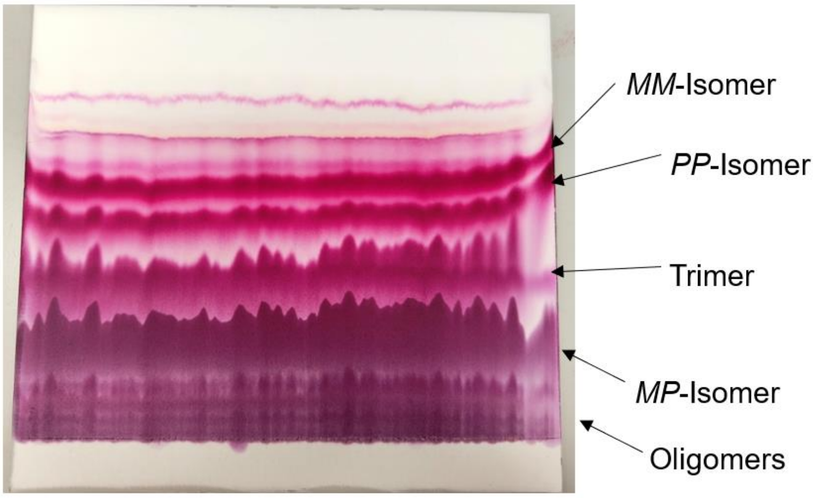

Supplementary Figure 1-2: Preparative TLC (1% EtOAc/CH_2Cl_2) of macrocycle.

HRMS (ESI$^+$) found 2173.7830, $[C_{136}H_{109}N_4O_{16}]^+$ requires 2173.7833.

MM Isomer:

^{1}H NMR (400 MHz, $CDCl_3$) δ/ppm 8.53 (s, 4H, **a**), 8.08 (d, *J* = 8.2 Hz, 4H, **b** or **c**), 7.86 – 7.34 (m, 56H, **i + b** or **c**), 5.02 (td, *J* = 10.0, 4.3 Hz, 4H, **d**), 4.86 (t, *J* = 10.6 Hz, 4H, **e'**), 4.65 (dd, *J* = 11.4, 4.3 Hz, 4H, **e''**), 3.10 (s, 4H, **h**), 2.73 – 2.59 (m, 4H, **f**), 1.14 (d, *J* = 6.5 Hz, 12H, **g'**), 0.83 (d, *J* = 6.6 Hz, 12H, **g''**).

^{13}C NMR (101 MHz, $CDCl_3$) δ/ppm 166.08, 164.37, 163.63, 141.20, 140.95, 140.92, 140.74, 140.67, 138.78, 135.37, 134.96, 132.58, 130.33, 130.27, 129.61, 129.30, 129.03, 128.78, 128.00, 127.84, 127.59, 127.23, 122.54, 121.64, 64.16, 58.63, 40.92, 29.85, 27.58, 20.75, 19.90.

PP Isomer:

^{1}H NMR (400 MHz, $CDCl_3$) δ/ppm 8.61 (s, 4H, **a**), 8.01 (d, *J* = 8.1 Hz, 4H, **b** or **c**), 7.87 – 7.33 (m, 56H, **i + b** or **c**), 5.05 (td, *J* = 10.3, 3.6 Hz, 4H, **d**), 4.89 (t, *J* = 10.8 Hz, 4H, **e'**), 4.67 (d, *J* = 11.1 Hz, 4H, **e''**), 3.17 (s, 4H, **h**), 2.70 (dt, *J* = 10.7, 6.6 Hz, 4H, **f**), 1.17 (d, *J* = 6.6 Hz, 12H, **g'**), 0.84 (d, *J* = 6.6 Hz, 12H, **g''**).

^{13}C NMR (101 MHz, $CDCl_3$) δ/ppm 166.07, 164.53, 163.54, 141.20, 140.94, 140.84, 140.61, 138.83, 136.07, 135.03, 132.63, 131.10, 129.78, 129.36, 129.02, 127.76, 127.57, 127.20, 122.09, 64.09, 58.99, 41.24, 29.85, 27.24, 20.83, 19.90.

MP Isomer:

^{1}H NMR (400 MHz, TCE-d_2) δ/ppm 8.67 (s, 2H, **a'**), 8.62 (s, 2H, **a''**), 8.29 – 7.34 (m, 64H, **b**, **c**, **i**), 4.96 (t, *J* = 11.4 Hz, 4H, **d**), 4.90 – 4.79 (m, 4H, **e'**), 4.72 (d, *J* = 8.5 Hz, 2H **e''**), 4.63 (d, *J* = 8.5 Hz, 2H, **e''**), 3.24 (s, 2H, **h'**), 3.23 (s, 2H, **h''**), 2.75 (dq, *J* = 13.4, 6.6 Hz, 2H, **f'**), 2.70 – 2.53 (m, 2H, **f''**), 1.19 (d, *J* = 6.6 Hz, 6H, **g'**), 1.16 (d, *J* = 6.5 Hz, 6H, **g''**), 0.87 (d, *J* = 6.5 Hz, 6H, **g'''**), 0.83 (d, *J* = 6.6 Hz, 6H, **g''''**).

^{13}C NMR (101 MHz, TCE-d_2) δ/ppm 166.00, 165.95, 164.13, 164.06, 163.45, 162.99, 141.19, 140.76, 140.68, 140.55, 140.43, 140.34, 140.21, 140.14, 140.08, 140.01, 138.30, 135.85, 135.11, 134.64, 132.51, 132.20, 128.92, 128.77, 127.64, 127.42, 127.35, 126.95, 126.85, 122.08, 121.89, 121.81, 121.17, 64.83, 64.56, 58.68, 41.45, 27.64, 27.25, 20.56, 20.46, 19.77.

^{1}H NMR (400 MHz, $CDCl_3$) δ/ppm 8.66 (s, 2H), 8.61 (s, 2H), 8.25 – 7.33 (m, 64H), 4.95 (q, *J* = 9.6 Hz, 4H), 4.90 – 4.81 (m, 2H), 4.77 – 4.67 (m, 2H), 4.58 (d, *J* = 9.0 Hz, 2H), 3.24 – 3.12 (m, 4H), 2.76 (dq, *J* = 13.3, 6.7 Hz, 2H), 2.62 (q, *J* = 7.4 Hz, 2H), 1.18 (dd, *J* = 10.1, 6.6 Hz, 12H), 0.83 (dd, *J* = 13.7, 6.6 Hz, 12H).

2) Spectroscopy

UV-Visible absorbance spectra were recorded on a Shimadzu UV-3600i Plus Spectrometer with a wavelength accuracy of ± 0.2 nm in the visible and UV region, a baseline flatness of ± 0.002 nm (200 – 3000 nm) and a noise level of <0.00008 Abs (900 nm) or a Cary50 UV-vis spectrometer.

Fluorescence spectra were recorded on a Cary Eclipse fluorescence spectrometer.

Quantum yields (Φ) were recorded on an Edinburgh Instruments FLS1000 Photoluminescence Spectrometer equipped with an integrating sphere and obtained using direct methods. Samples were prepared at ~0.1 OD at the excitation wavelength. Spectra were recorded with an excitation bandwidth of 5 nm, an emission bandwidth of 0.85 nm (for **5**) or 0.90 nm (for **8a-c**) with 0.5 nm steps and a dwell time of 0.5 s. Quantum yields were calculated using the Fluoracle software, and errors of the calculated yields are ±2%.

Circular Dichroism measurements were taken on a JASCO J-1500 CD Spectrophotometer with a wavelength accuracy ± 0.2 nm (250 to 500 nm), ± 0.5 nm (500 to 800 nm), a photometric accuracy of ± 0.01 Abs and a CD root mean square noise < 0.007 mdeg (500 nm). Recorded traces were then baseline corrected and smoothed using the provided software.

CPL was measured with a home-built (modular) spectrometer.[1] The excitation source was a broad band (200 – 1000 nm) laser- driven light source EQ 99 (Elliot Scientific). The excitation wavelength was selected by feeding the broadband light into an Acton SP-2155 monochromator (Princeton Instruments, 7 μm slit); the collimated light was focused into the sample cell (1 cm quartz cuvette, temperature controlled at 20 ºC). Sample PL emission was collected perpendicular to the excitation direction with a lens (f = 150 mm). The emission was fed through a photoelastic modulator (PEM) (Hinds Series II/FS42AA) and through a linear sheet polariser (Comar). The light was then focused into a second scanning monochromator (Acton SP-2155, 7 μm slit) and subsequently on to a photomultiplier tube (PMT) (Hamamatsu H10723 series). The detection of the CPL signal was achieved using the field modulation lock-in technique. The electronic signal from the PMT was fed into a lock-in amplifier (Hinds Instruments Signaloc Model 2100). The reference signal for the lock-in detection was provided by the PEM control unit. The monochromators, PEM control unit and lock-in amplifier were interfaced to a desktop PC and controlled by a custom-written Labview graphic user interface. The lock-in amplifier provided two signals, an AC signal corresponding to (I_L- I_R) and a DC signal corresponding to (I_L + I_R) after background subtraction. The emission dissymmetry factor was therefore readily obtained from the experimental data, as 2 AC/DC. Errors of g_{lum} are ±5 x 10^{-5}. The emission spectra were recorded with 2 nm step size and the slits of the detection monochromator were set to a slit width corresponding to a spectral resolution of 3.5 nm. CPL spectra (as well as total emission spectra) were obtained through an averaging procedure of several scans. Measurements were performed in $CHCl_3$ at ~0.1 OD (~2.5 μM).

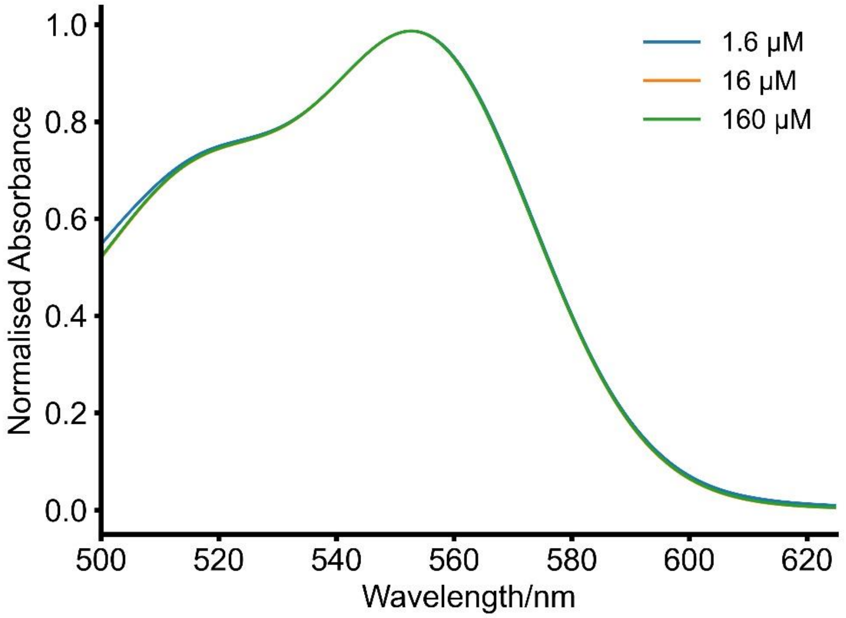

Supplementary Figure 2-1: Normalised absorbance plots for compound **3** in $CHCl_3$ at three different concentrations. The unchanged spectral shape and consistent A_{0-0}/A_{0-1} ratio indicate that the molecule is monomeric over the range of concentrations tested.

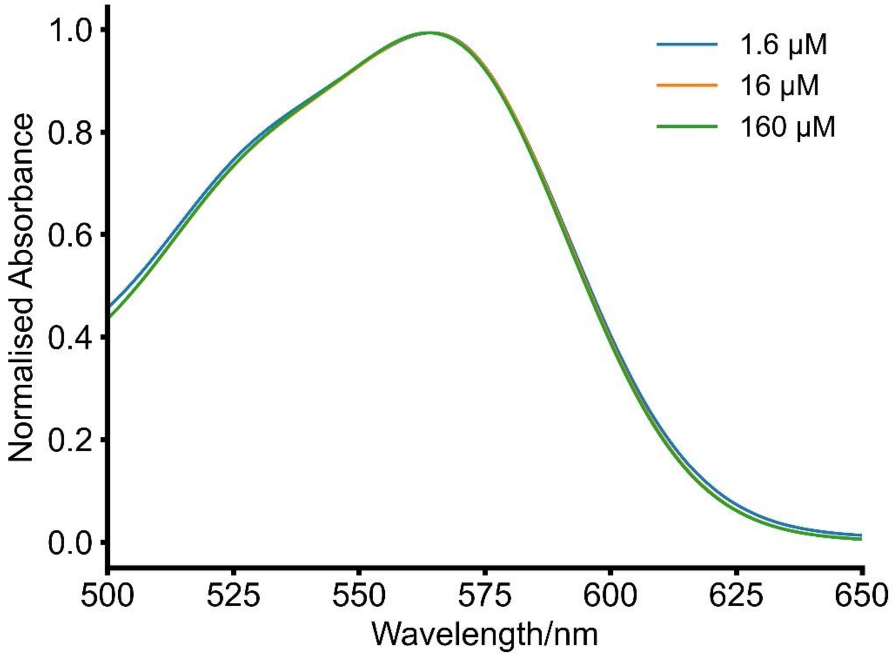

Supplementary Figure 2-2: Normalised absorbance plots for compound **6** in $CHCl_3$ at three different concentrations. The unchanged spectral shape and consistent A_{0-0}/A_{0-1} ratio indicate that the molecule is monomeric over the range of concentrations tested.

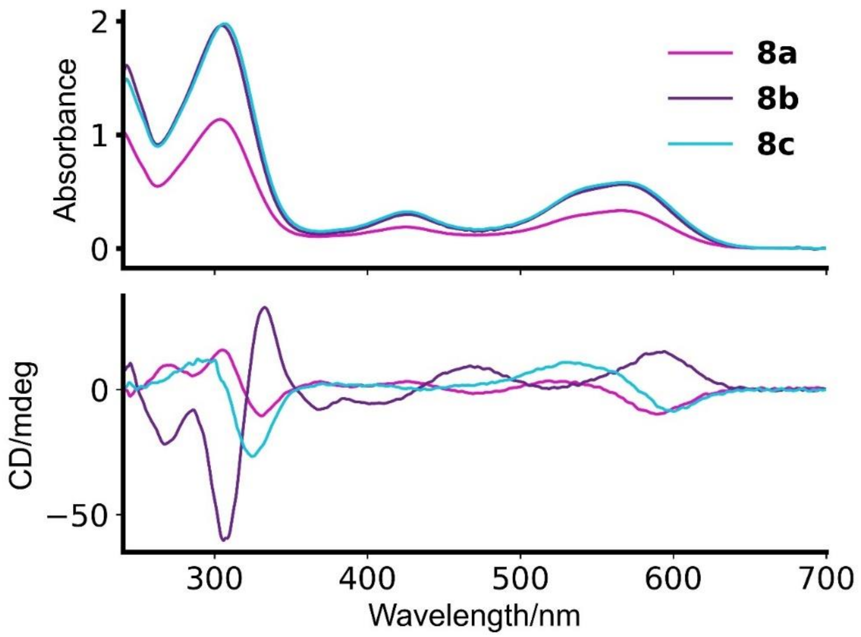

Supplementary Figure 2-3: Raw absorbance and CD spectra for the 3 isomers of **8** in $CHCl_3$.

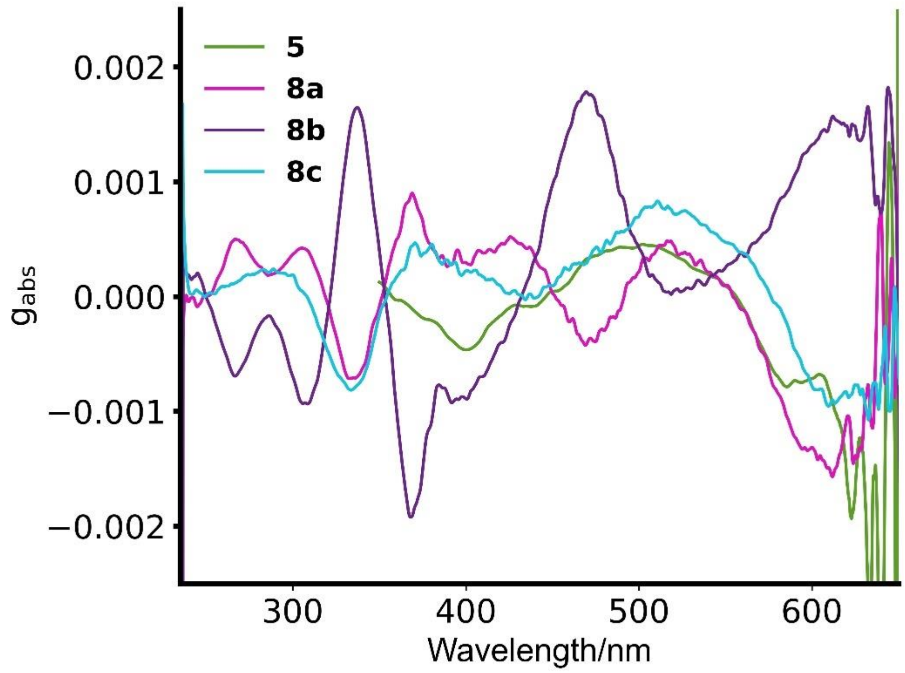

Supplementary Figure 2-4: Plot of g_{abs} of the individual macrocycles in $CHCl_3$.

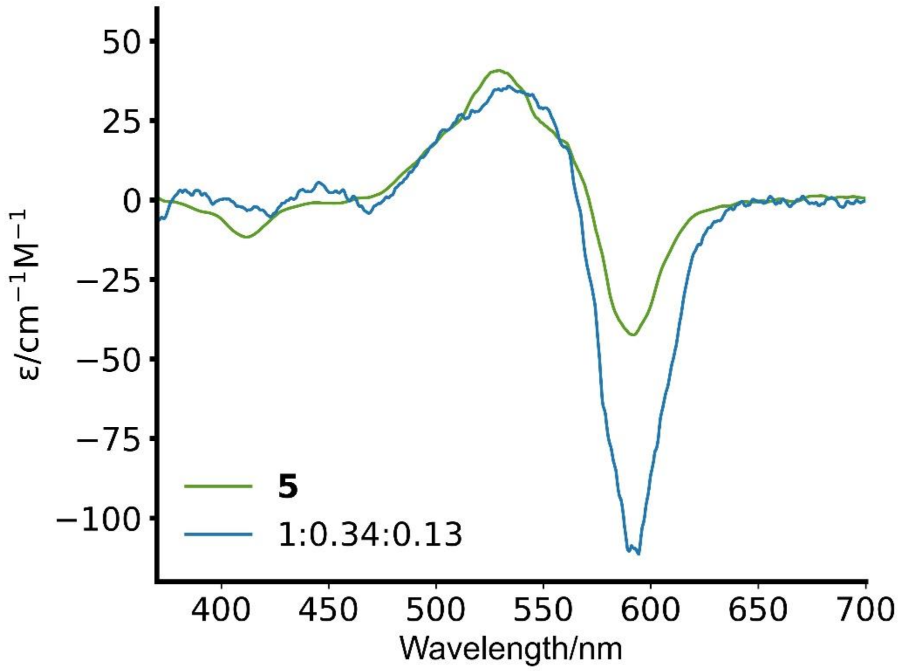

Supplementary Figure 2-5: Comparison of dynamic macrocycle **5**'s CD (shifted by +15 nm for easier comparison) with weighted averages of *MP:MM:PP* isomers of **8**, according to NMR integration.

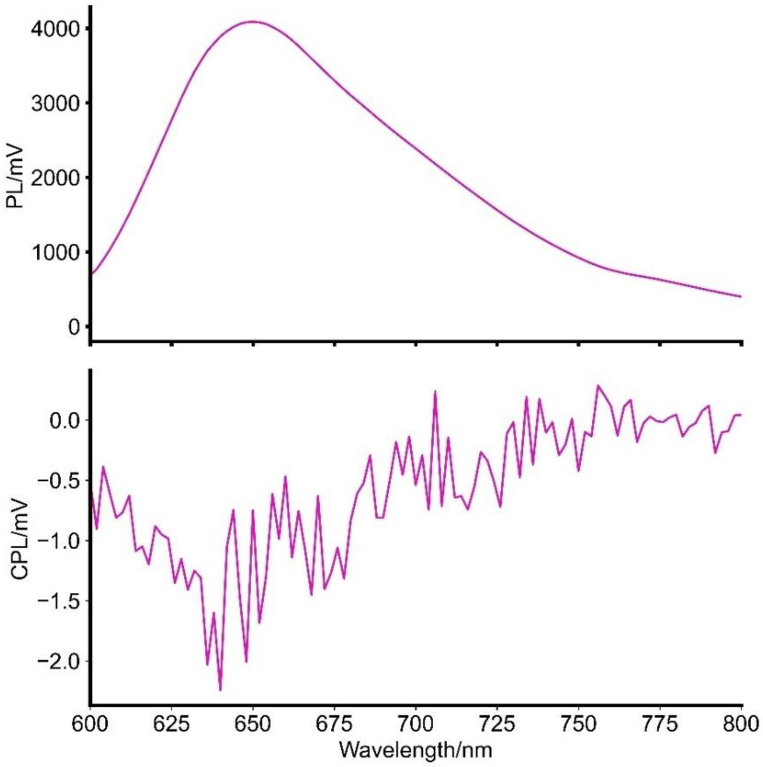

Supplementary Figure 2-6: PL and CPL of *MM*-isomer macrocycle **8a** in $CHCl_3$. λ_{ex} = 550 nm.

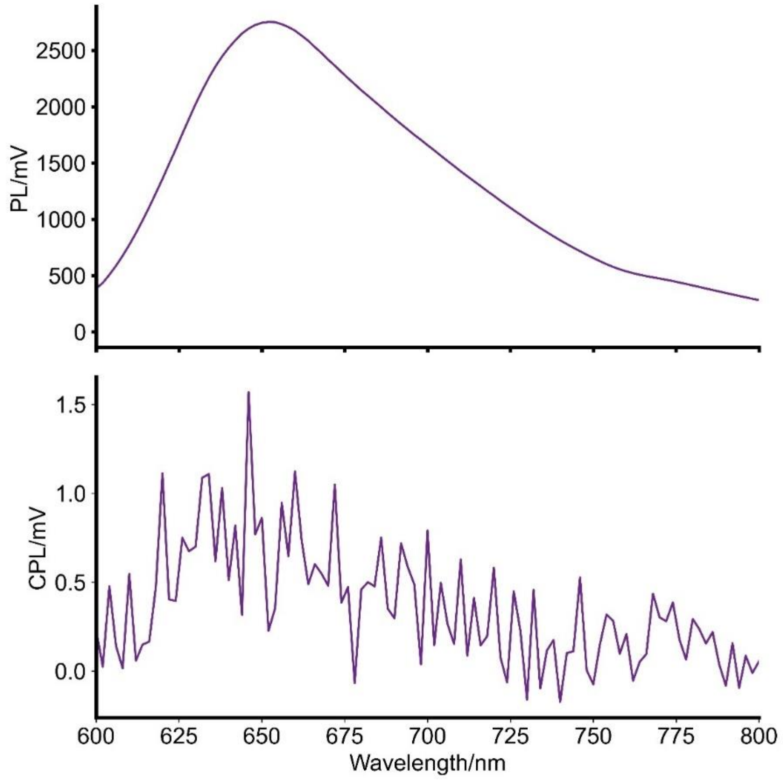

Supplementary Figure 2-7: PL and CPL of *PP*-isomer macrocycle **8b** in $CHCl_3$. λ_{ex} = 550 nm.

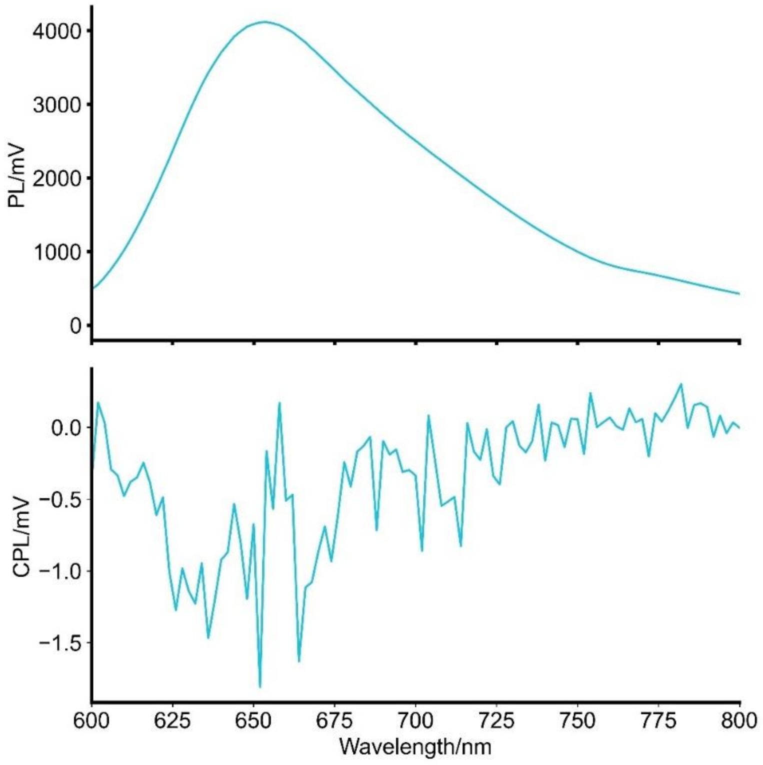

Supplementary Figure 2-8: PL and CPL of *MP*-isomer macrocycle **8c** in $CHCl_3$. λ_{ex} = 550 nm.

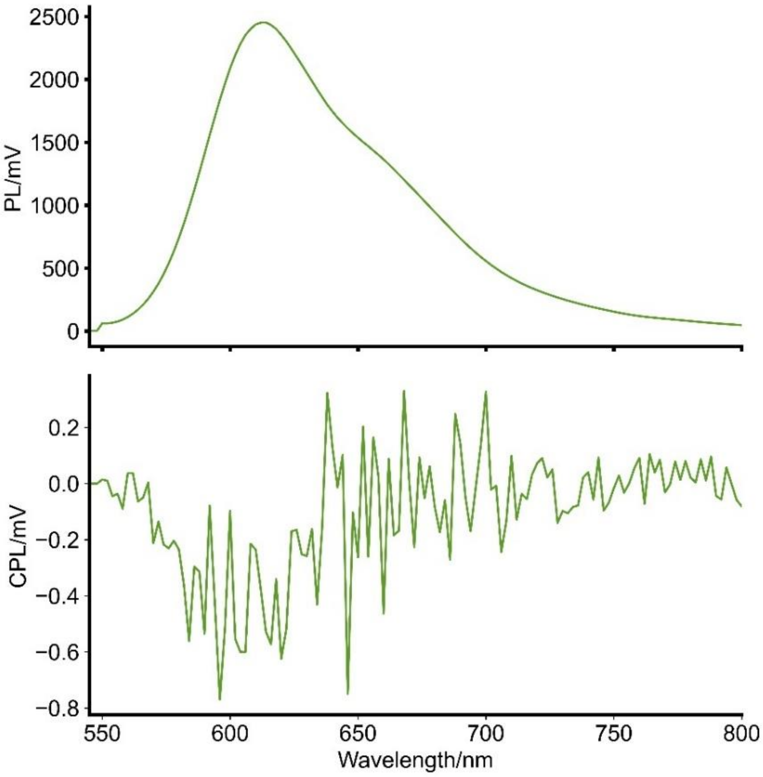

Supplementary Figure 2-9: PL and CPL of macrocycle **5** in $CHCl_3$. λ_{ex} = 520 nm.

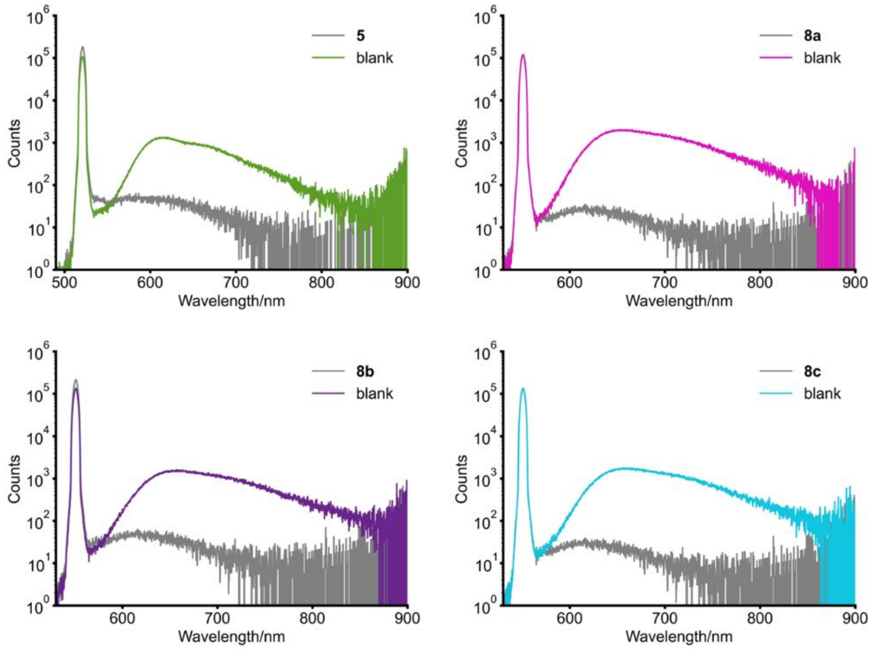

Supplementary Figure 2-10: Quantum yield measurements of macrocycles **5** and **8a-c** in $CHCl_3$. Samples were prepared at an OD <0.1 at the excitation wavelength. λ_{ex} = 520 nm for **5** and λ_{ex} = 550 nm for **8a-c**.

3) Chiral HPLC

Chiral resolution HPLC was performed on a Phenomenex Lux 5 µm i-Amylose-1 (C 250 x 4.6 mm) column using a gradient of 5-30% CH_2Cl_2 in Hexane over 30 minutes at a flow rate of 1 ml/min.

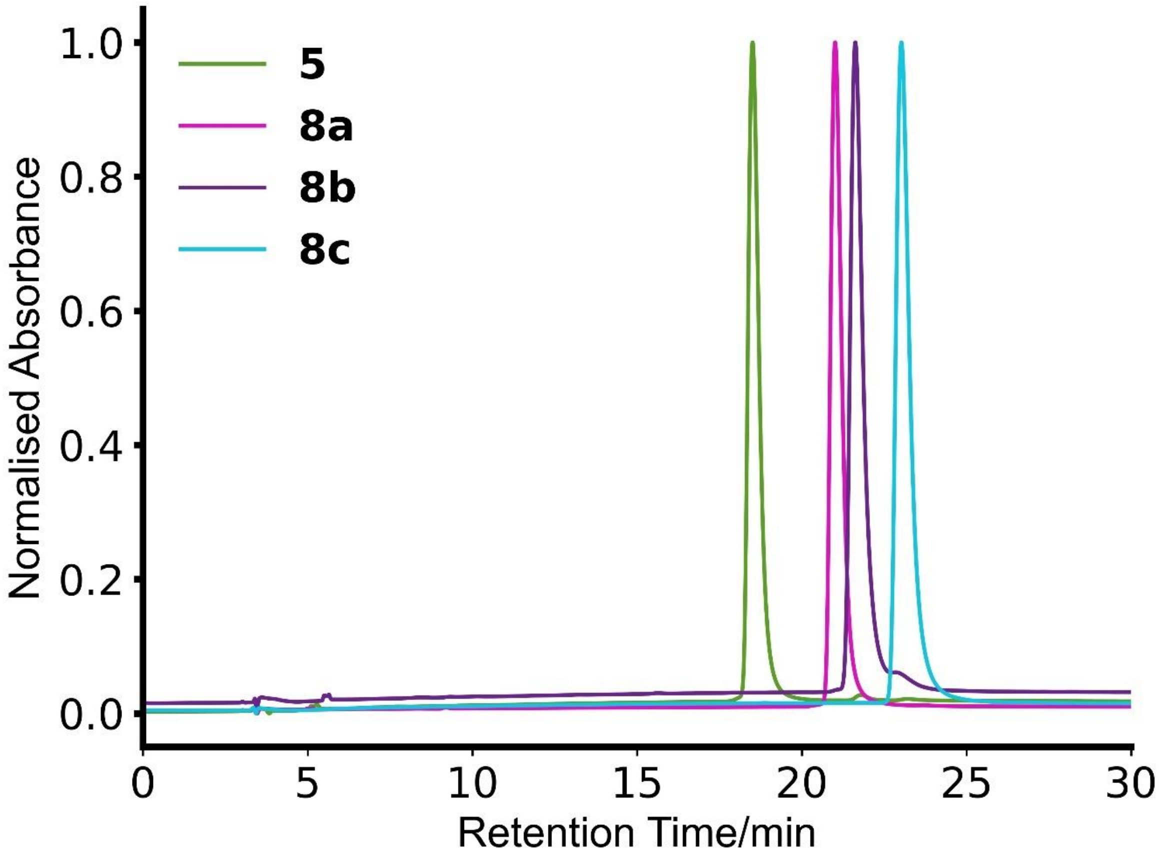

Supplementary Figure 3-1: Chromatogram of the macrocycles on the amylose column. Note that the order of elution mimics the order found on preparative TLC.

4) Host/Guest Chemistry

General

All host/guest titrations were performed in $CHCl_3$ at ~10 μM host species and links to the fitting are given with each titration. The best fit was obtained using a 1:1 host–guest stoichiometric model, with all fitting errors ≤ 1 %. Further justification for the 1:1 stoichiometric binding model comes from the random distribution of residuals because deviation from the theoretical binding isotherm is caused by random experimental errors.[2] Alternative 1:2 and 2:1 host–guest stoichiometric binding models were ruled out due to non-sensical K_a values, and higher fitting errors.

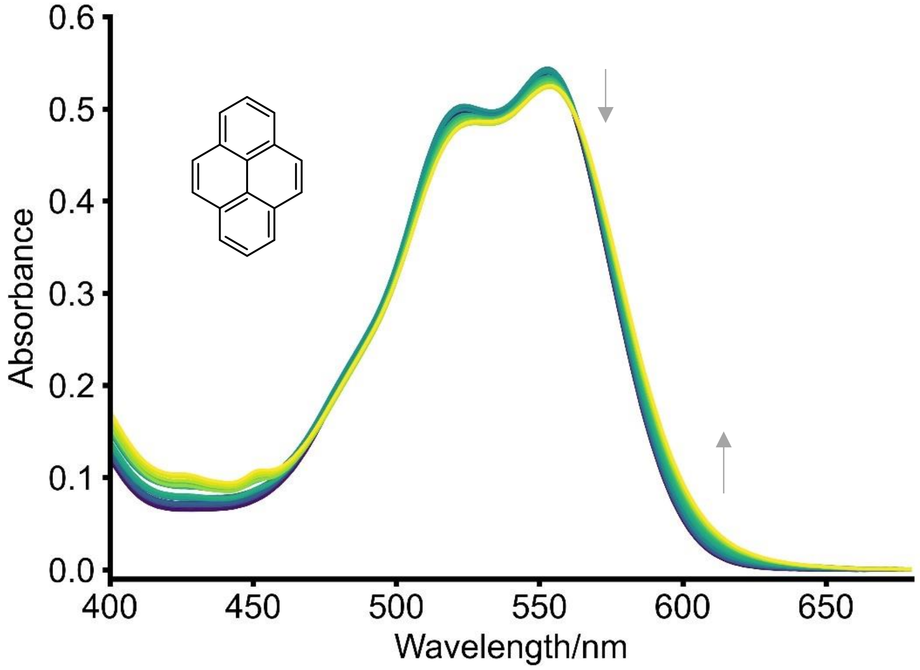

Supplementary Figure 4-1: Changes in absorbance upon titration of pyrene (0-36 mM, purple to yellow) into single-Ph-substituted PDI macrocycle **5** (10 μM) in $CHCl_3$. Non-linear curve fitting of the region of 575-620 nm to a 1:1 binding model yielded K_a = 39 M^{-1} (± 0.6%).

http://app.supramolecular.org/bindfit/view/b0ed6811-7951-4860-b022-2731cc4a4d40.

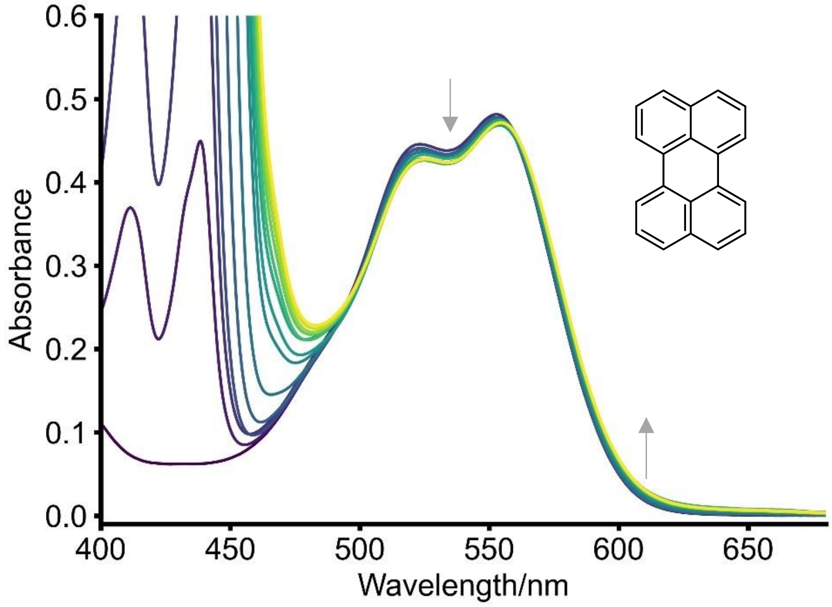

Supplementary Figure 4-2: Changes in absorbance upon titration of perylene (0-3 mM, purple to yellow) into single-Ph-substituted PDI macrocycle **5** (10 μM) in $CHCl_3$. Non-linear curve fitting of the region of 565-625 nm to a 1:1 binding model yielded K_a = 223 M^{-1} (± 0.8%).

http://app.supramolecular.org/bindfit/view/227d6ecb-0e40-464d-8c0b-949685cd1a87

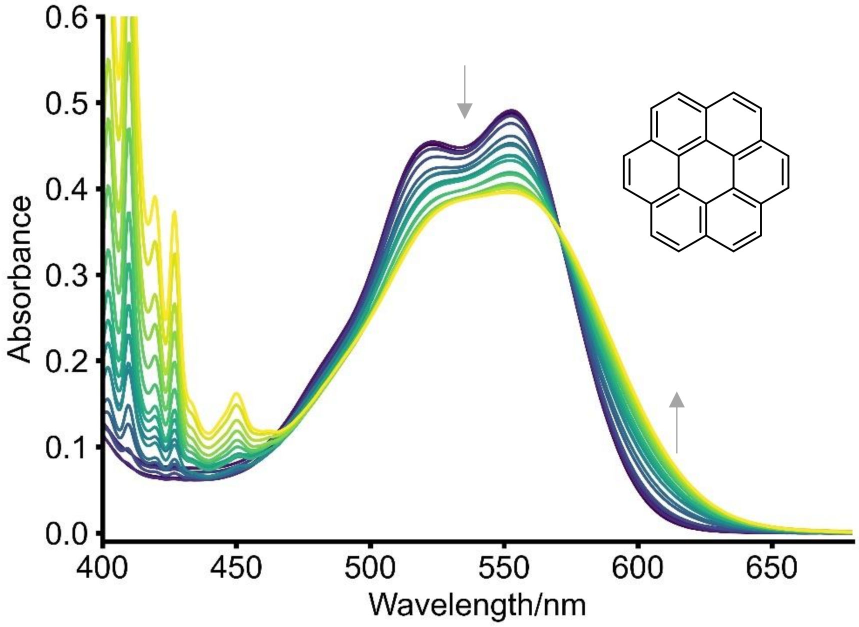

Supplementary Figure 4-3: Changes in absorbance upon titration of perylene (0-2.1 mM, purple to yellow) into single-Ph-substituted PDI macrocycle **5** (10 μM) in $CHCl_3$. Non-linear curve fitting of the region of 525-665 nm to a 1:1 binding model yielded K_a = 2493 M^{-1} (± 0.4%).

http://app.supramolecular.org/bindfit/view/6019ca27-3d54-4917-95ca-47431c5e8169

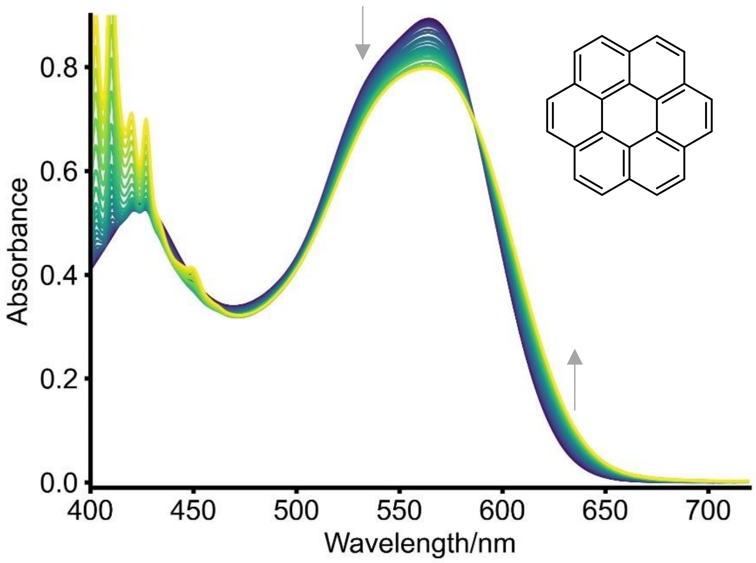

Supplementary Figure 4-4: Changes in absorbance upon titration of perylene (0-1.54 mM, purple to yellow) into the *MM*-isomer of terphenyl-substituted PDI macrocycle **8a** (10 µM) in $CHCl_3$. Non-linear curve fitting of the region of 475-700 nm to a 1:1 binding model yielded K_a = 3689 M^{-1} (± 0.2%).

http://app.supramolecular.org/bindfit/view/46fe533a-d149-443e-bd52-7e96355182a1

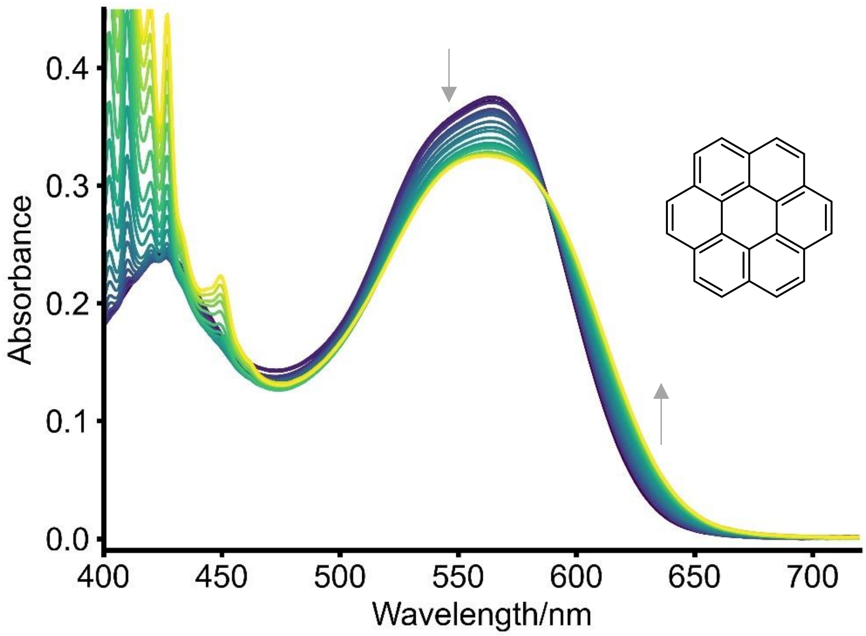

Supplementary Figure 4-5: Changes in absorbance upon titration of perylene (0-1.52 mM, purple to yellow) into the *PP*-isomer of terphenyl-substituted PDI macrocycle **8b** (10 µM) in $CHCl_3$. Non-linear curve fitting of the region of 525-650 nm to a 1:1 binding model yielded K_a = 3536 M^{-1} (± 0.4%).

http://app.supramolecular.org/bindfit/view/aa6ea5c9-287e-4284-8e3f-4c130bf4103b

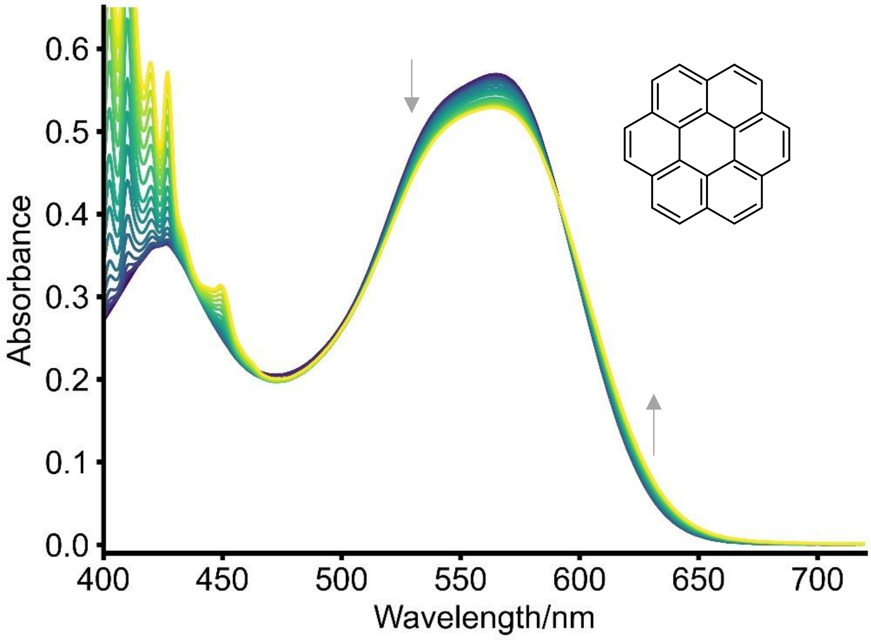

Supplementary Figure 4-6: Changes in absorbance upon titration of perylene (0-1.68 mM, purple to yellow) into the *MP*-isomer of terphenyl-substituted PDI macrocycle **8c** (12 μM) in $CHCl_3$. Non-linear curve fitting of the region of 555-630 nm to a 1:1 binding model yielded K_a = 581 M^{-1} (± 0.5%).

http://app.supramolecular.org/bindfit/view/c1370944-c367-457f-9216-f8fea6942651

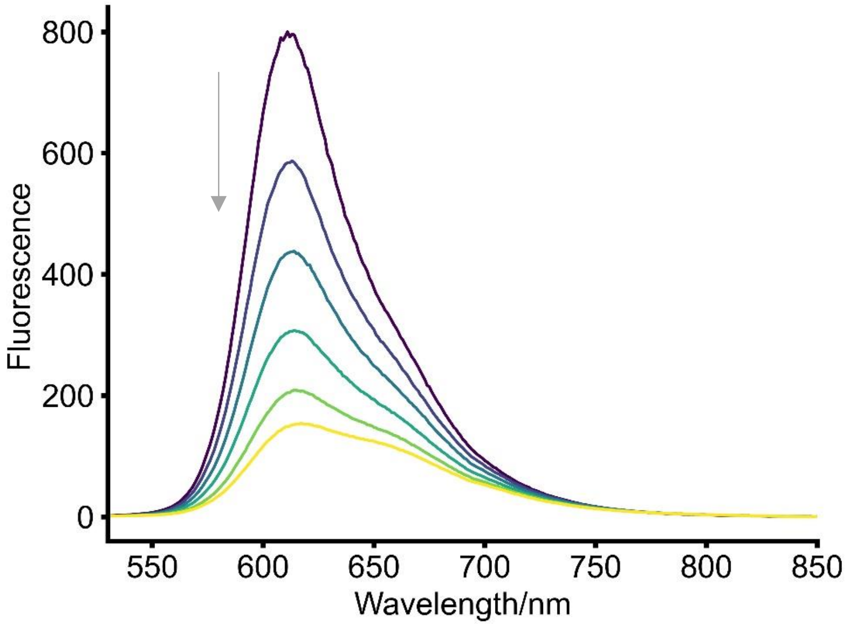

Supplementary Figure 4-7: Changes in fluorescence of single Ph-substituted macrocycle **5** in $CHCl_3$ (10 μM) upon addition of coronene (0%, 12%, 25%, 40%, 54%, 66% complexation). λ_{ex} = 520 nm.

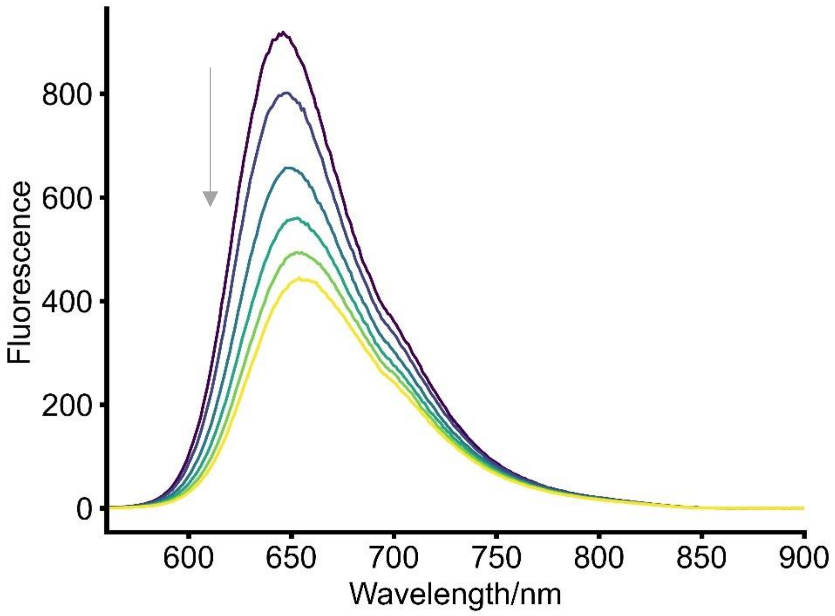

Supplementary Figure 4-8: Changes in fluorescence of the *MM*-isomer of terphenyl-substituted macrocycle **8a** in $CHCl_3$ (10 μM) upon addition of coronene (0%, 20%, 50%, 70%, 80%, 87% complexation). λ_{ex} = 550 nm.

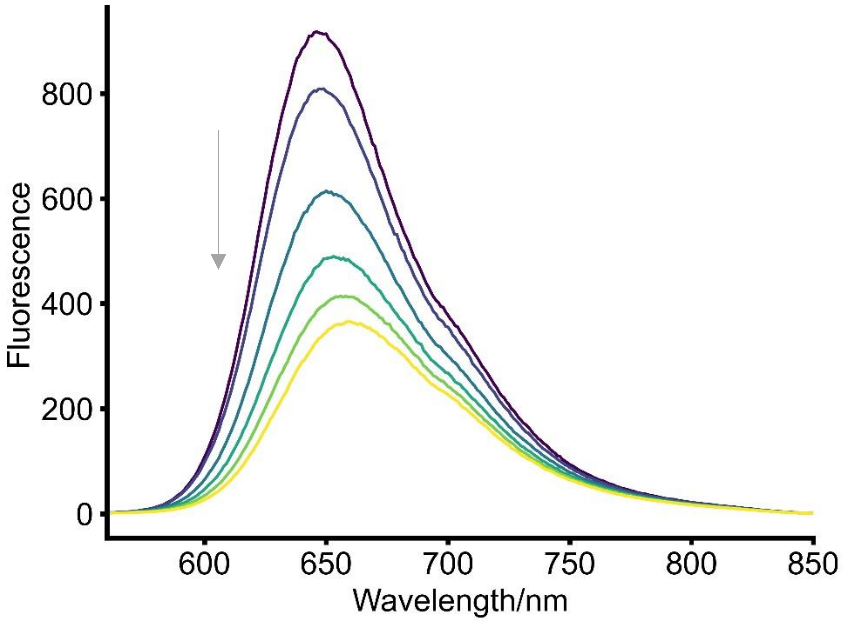

Supplementary Figure 4-9: Changes in fluorescence of the *PP*-isomer of terphenyl-substituted macrocycle **8b** in $CHCl_3$ (10 uM) upon addition of coronene (0%, 20%, 50%, 70%, 80%, 87% complexation). λ_{ex} = 550 nm.

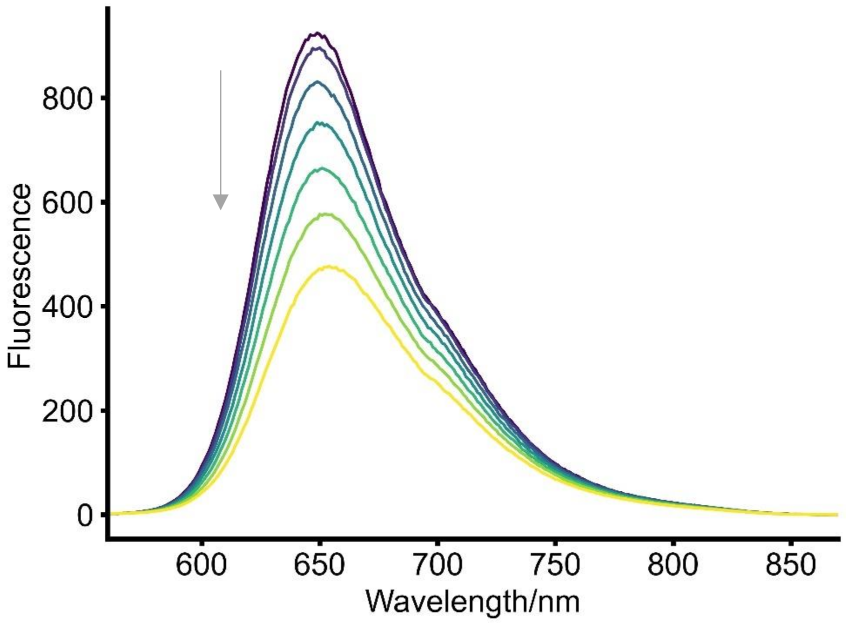

Supplementary Figure 4-10: Changes in fluorescence of the MP-isomer of terphenyl-substituted macrocycle **8c** in $CHCl_3$ (10 μM) upon addition of coronene (0%, 5%, 15%, 25%, 37%, 55% and 70% complexation). λ_{ex} = 550 nm.

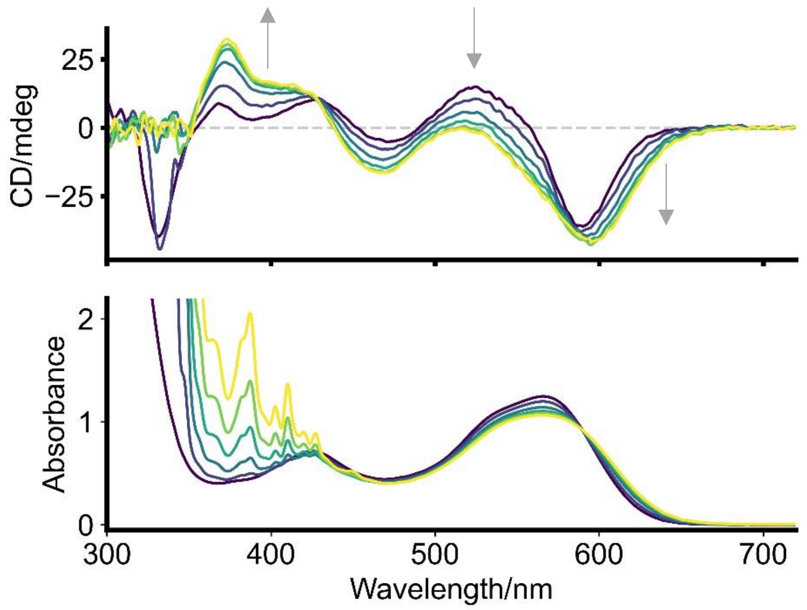

Supplementary Figure 4-11: Changes in CD spectrum of *MM*-isomer **8a** of terphenyl-substituted macrocycle (15 μM) upon addition of coronene in $CHCl_3$ (up to 90% complexation).

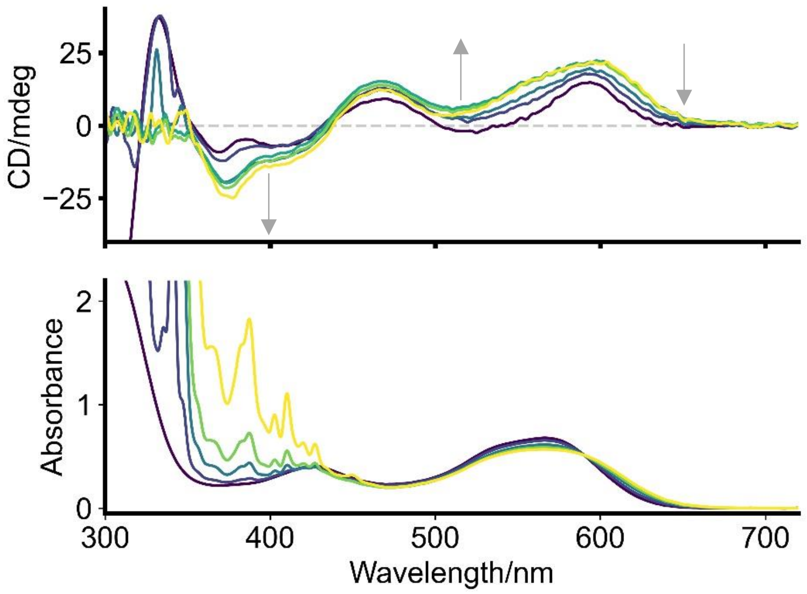

Supplementary Figure 4-12: Changes in CD spectrum of *PP*-isomer **8b** of terphenyl-substituted macrocycle (15 μM) upon addition of coronene in $CHCl_3$ (up to 90% complexation).

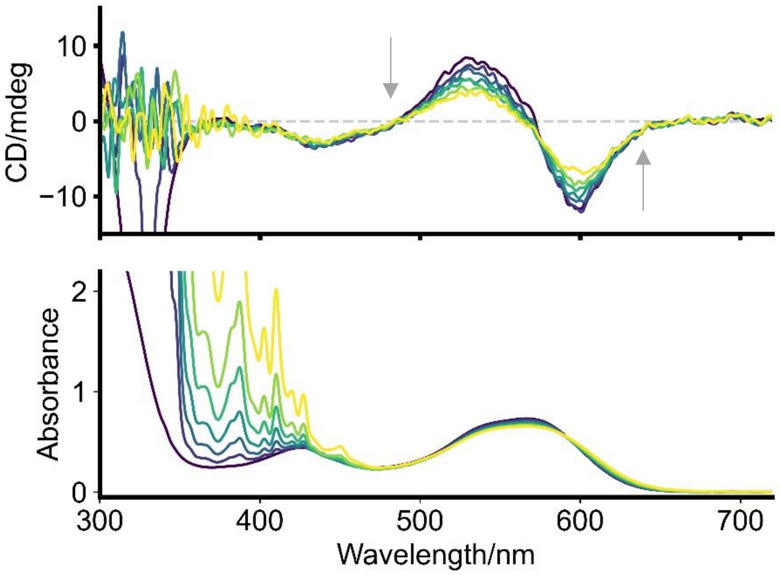

Supplementary Figure 4-13: Changes in CD spectrum of *MP*-isomer **8c** of terphenyl-substituted macrocycle (10 µM) upon addition of coronene in $CHCl_3$ (up to 70% complexation).

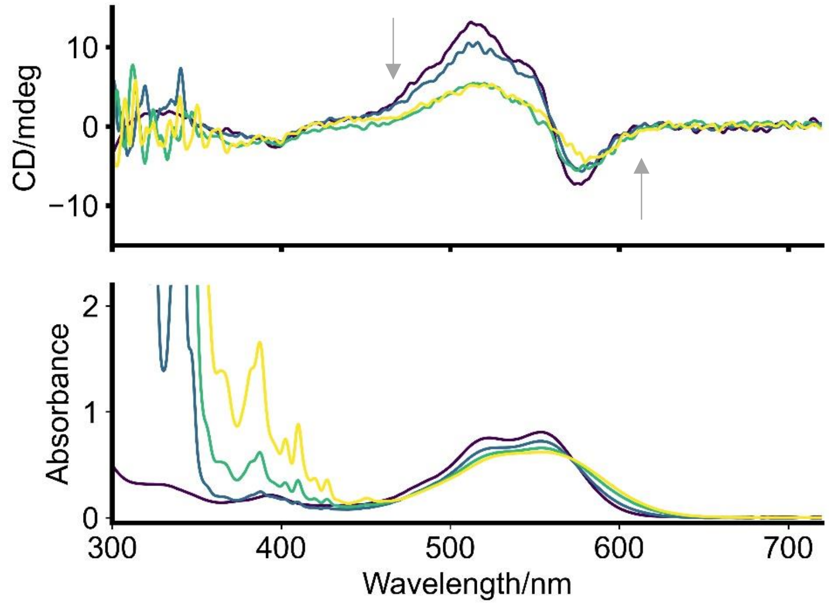

Supplementary Figure 4-14: Changes in CD spectrum of single Ph-substituted Macrocycle **5** (10 µM) upon addition of coronene in $CHCl_3$ (up to 83% complexation).

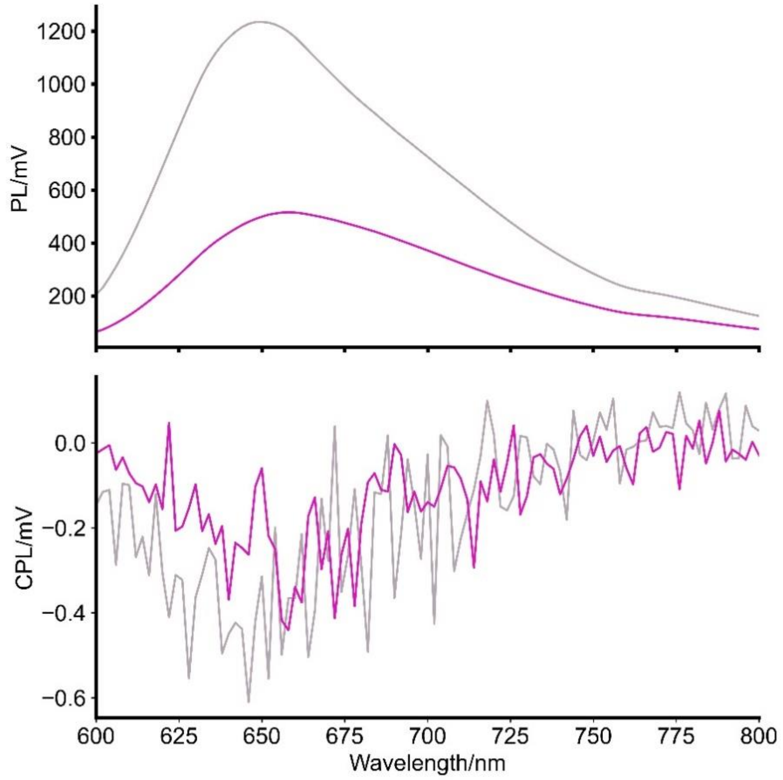

Supplementary Figure 4-15: PL and CPL of *MM*-isomer macrocycle **8a** before (grey) or after (magenta) addition of 1000 equivalents of coronene in $CHCl_3$.

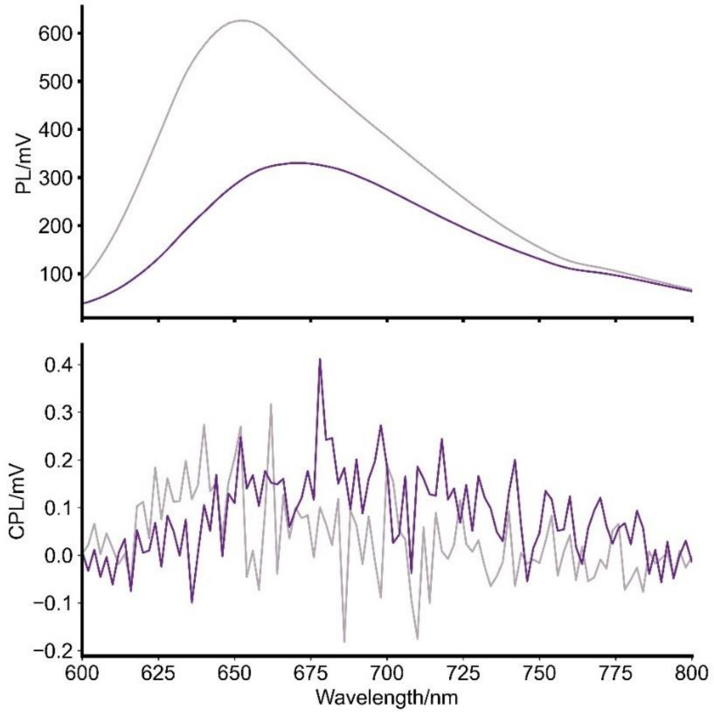

Supplementary Figure 4-16: PL and CPL of *PP*-isomer macrocycle **8b** before (grey) or after (purple) addition of 1000 equivalents of coronene in $CHCl_3$.

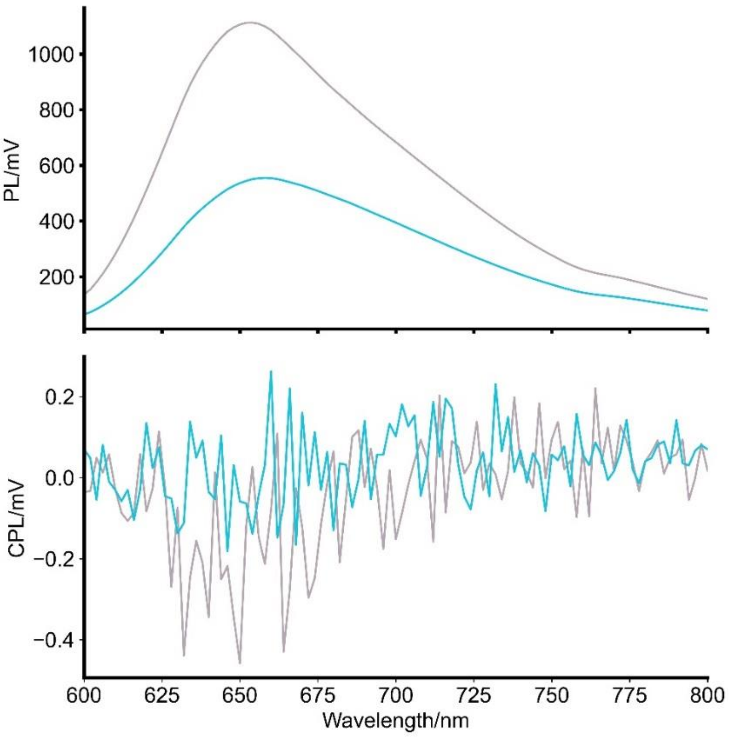

Supplementary Figure 4-17: PL and CPL of *MP*-isomer macrocycle **8c** before (grey) or after (cyan) addition of 1000 equivalents of coronene in $CHCl_3$.

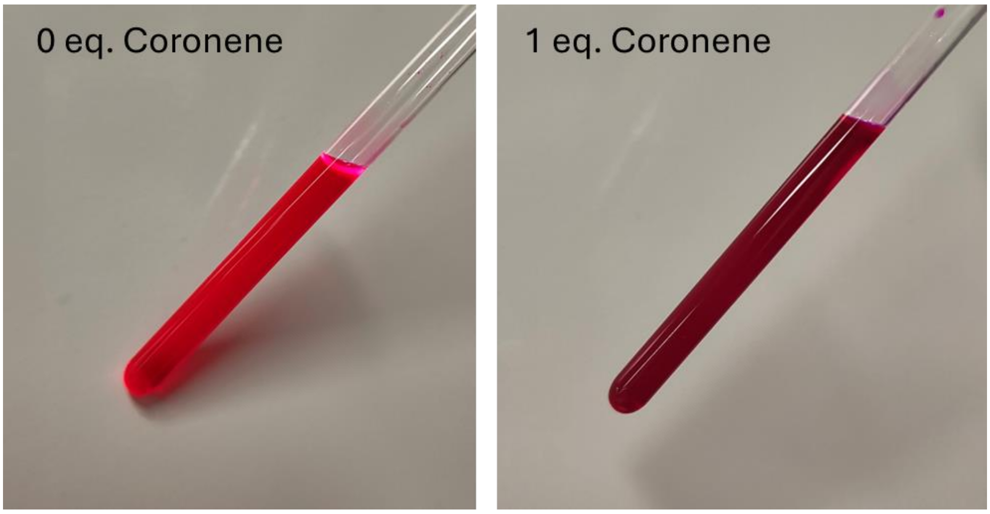

Supplementary Figure 4-18: Colour change of macrocycle **5** in $CDCl_3$ (1.6 mM) upon addition of 1 equivalent of coronene.

5) Crystallography

M/P-isomer of the Bis(terphenyl)-L-Valinol PDI Macrocycle

Crystals of the M/P isomer macrocycle were grown by vapour diffusion of hexane into a solution of the macrocycle in $CHCl_3$. Single crystal X-ray diffraction experiments for the obtained red crystals were performed by the UK EPSRC National Crystallography Service at the University of Southampton[3] on a Rigaku 007HF diffractometer with HF Varimax confocal mirrors, an UG2 goniometer and HyPix 6000HE detector at 100 K using Cu K_α radiation and processed using CrysAlisPro 1.171.43.

All crystals screened were twinned; data was therefore collected on a crystal that showed the least observable amount of twinning. Whilst there is confidence in the resulting structure, it was not possible to integrate and account for the X-ray diffraction of the minor twin component(s). The resulting structure is therefore derived from integrating only the principle diffraction pattern; consequently overlap from the minor twin component(s) have lowered the quality of the statistics of the refined model and likely also reduced bond accuracy.

The hydrogen atoms were fixed as riding models and the isotropic thermal parameters (U_{iso}) based on the U_{eq} of the parent atom. The structure was solved by direct methods using ShelXT[4] and refined with ShelXL[5] using a least squares method. Olex2 software was used as the solution, refinement and analysis program.[6]

The structure showed strongly disordered regions of solvent, which could not be accurately modelled. A solvent mask was therefore applied. The electron density in the disordered region was 120 e^-, which are likely to be 2 hexane molecules (100 e^-). The difference in electron density can at least partially be attributed to the twinning and therefore the structure quality.

Crystal Data for $C_{159}H_{137}Cl_3N_4O_{16}$ (M =2466.07 g/mol): monoclinic, space group $P2_1$ (no. 4), a = 20.1924(4) Å, b = 16.9083(2) Å, c = 20.5086(4) Å, $\alpha = \gamma = 90°$, β = 118.533(2)°, V = 6151.6(2) Å^3, Z = 2, T = 100 (10) K, μ(Cu Kα) = 1.257 mm^{-1}, $Dcalc$ = 1.331 g/cm^3, 118220 reflections measured (7.17° ≤ 2Θ ≤ 144.204°), 22806 unique (R_{int} = 0.1002, R_{sigma} = 0.0658) which were used in all calculations. The final R_1 was 0.0622 (I > 2σ(I)) and wR_2 was 0.1726 (all data).

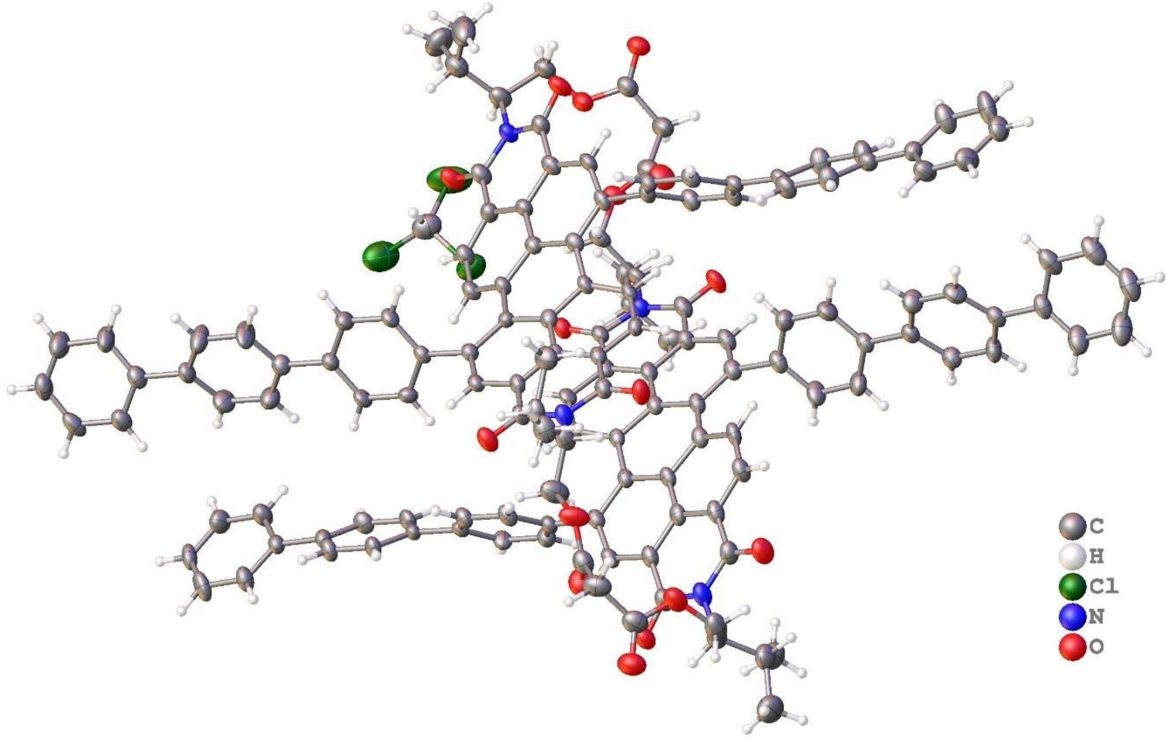

Supplementary Figure 5-1: Asymmetric unit of the *MP*-isomer single crystal structure. Thermal ellipsoids show 50% probability.

6) Computational Chemistry

General

Terphenyl groups have been simplified as Ph groups for all calculations. Density functional theory (DFT) calculations were performed using ORCA 6.0.[7] All functionals and basis sets were chosen following the best-practice DFT protocols published by Grimme.[8]

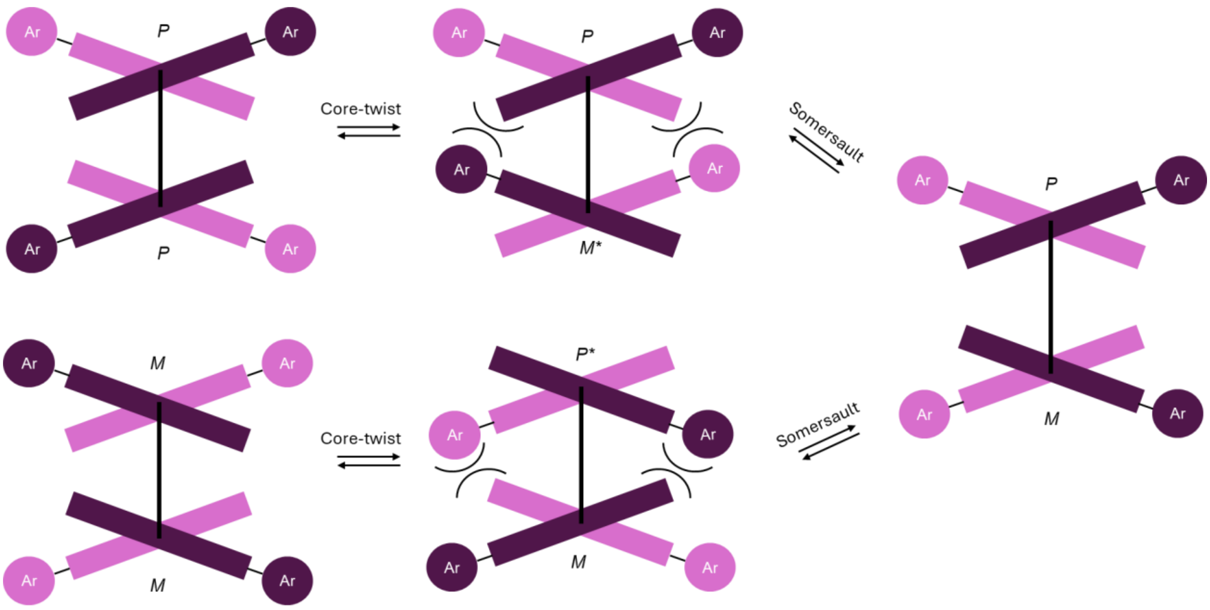

Supplementary Figure 6-1: Proposed mechanism for interconversion between stereoisomers of the bis-PDI macrocycles. Homochiral (*MM* or *PP*) stacking is the lowest energy conformer, with a low barrier core twist to a higher energy state (*MP** or *PM**), disfavoured due to steric clash. A somersault through the cavity (**Supplementary Figure 6-2**) then allows for the population of the heterochiral *MP* isomer.

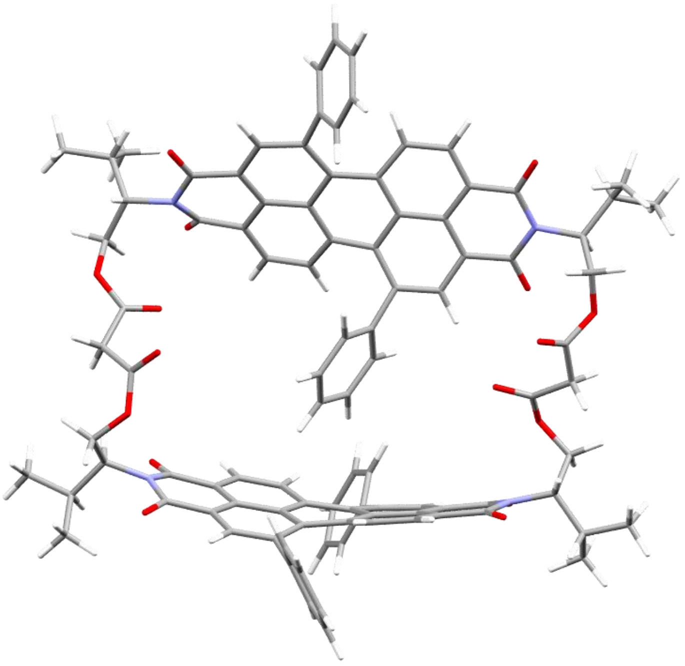

Supplementary Figure 6-2: Depiction of an intermediate step in the somersault mechanism. The bay-substituent is rotating through the macrocycle cavity. This is possible for a small phenyl substituent, but not for an elongated substituent such as terphenyl, as its length exceeds the macrocycle cavity diameter (**Supplementary Figure 6-3**). Molecule optimised using a force-field approach.

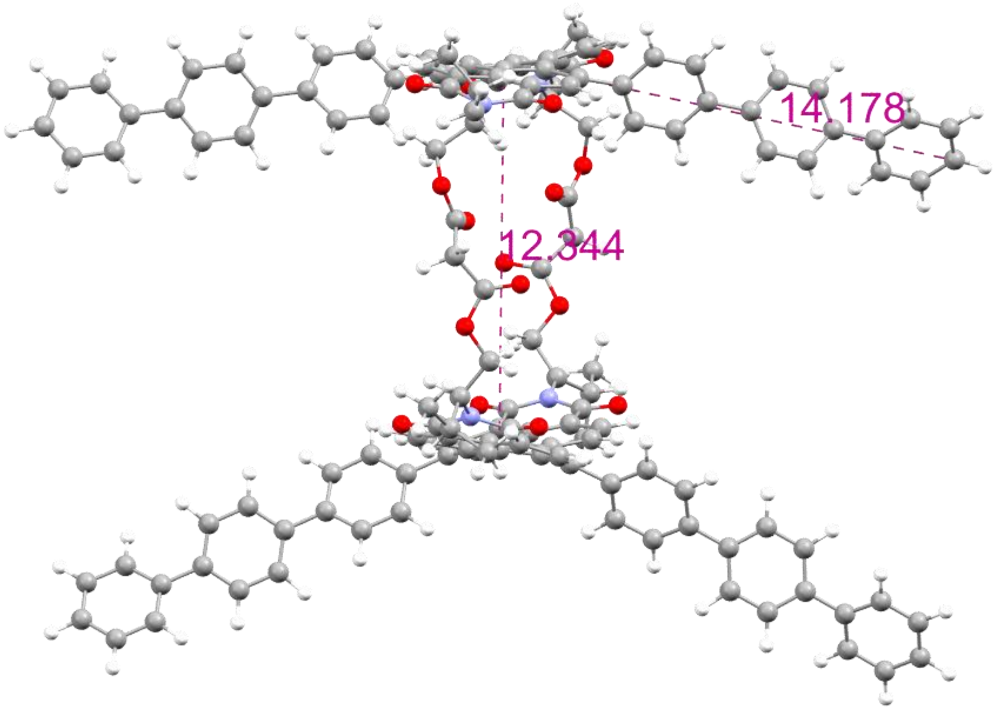

Supplementary Figure 6-3: Terphenyl-substituted macrocycle at maximum expansion, optimised using a force field approach in Avogadro, with cavity diameter and terphenyl-substituent lengths (in Å) indicated.

M/P core-twist interconversion barrier

M and *P* isomers of a Ph-substituted PDI were optimised using the B97-3c functional[9] and the def2-TZVP basis set[10] with implicit solvation in $CHCl_3$ using the conductor-like polarizable continuum model (CPCM).[11] The transition state was then computed using the nudged elastic band climbing image (NEB-CI) approach. Single point energies for the *M*- and *P*-isomer as well as the transition state were then computed using a higher level of theory using the PWPB95 functional[12] and the RIJK approximation[13] with a def2-QZVP basis set and def2/JK and def2-TZVP/C auxiliary basis sets with D3BJ dispersion correction.[14]

Supplementary Table 6-1: Summary of calculated single point energies for the individual isomers (PWPB95/def2-QZVP).

Molecule	Energy (Eh)
M	-1871.453909
P	-1871.453497
TS	-1871.440678

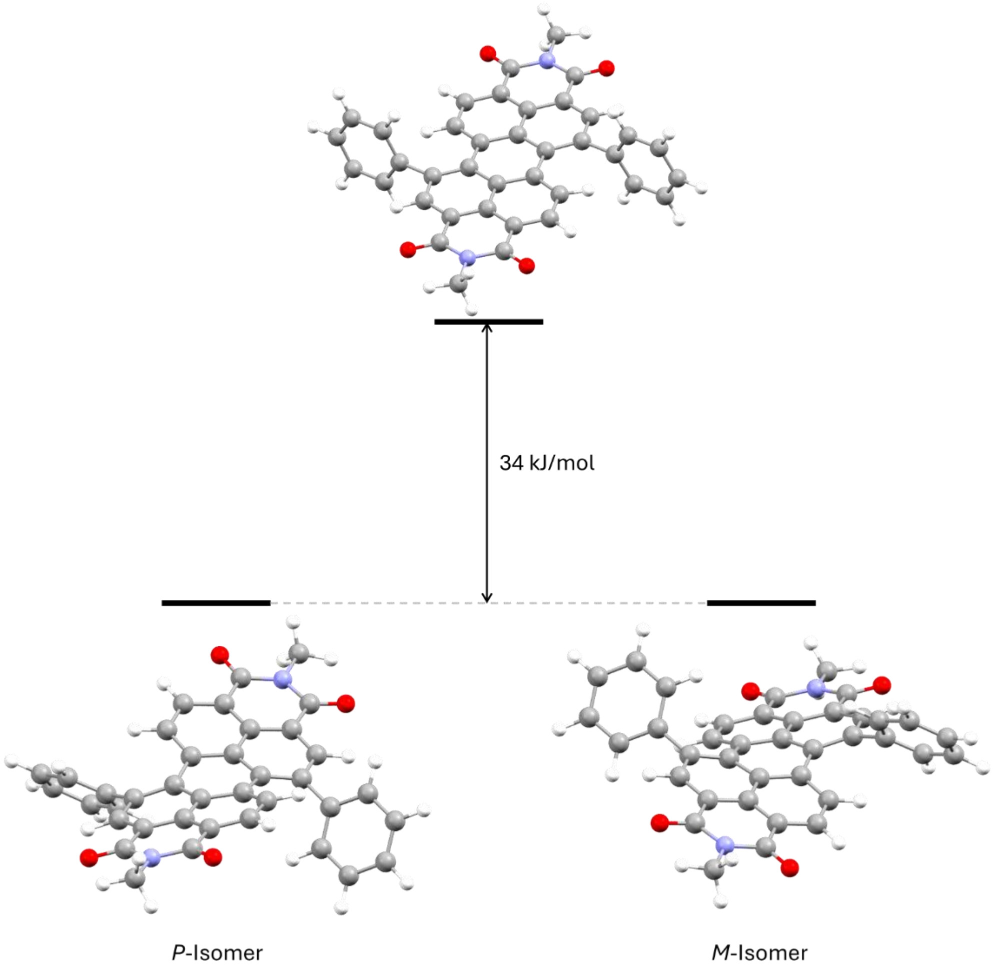

Supplementary Figure 6-4: DFT-optimised structures of the isomers and their transition state, with the energy difference highlighted. Energy difference was calculated from the average energy of *M* and *P* isomer to the transition state.

Conformer searches

The *MM*, *PP* and *MP*-Isomers were predicted using a combination of the CREST[15] and the GFN2-xTB semiempirical tight-binding method.[16,17]

DFT optimisation

Structures obtained by CREST were subsequently optimised by means of DFT, using the B97-3c functional by Grimme and co-workers,[9] and the def2-TZVP basis set[10] with implicit solvation in $CHCl_3$ using the conductor-like polarizable continuum model (CPCM).[11]

Spectrum prediction

Vertical excitation and circular dichroism spectra were predicted using TD-DFT in $CHCl_3$. The calculations employed the TD-ωB97x[18] functional, the def2-SVP basis-set,[10] and the CPCM solvation model[11] and were performed on top of the B97-3c optimised structures.

Supplementary Table 6-2: Summary of calculated single point energies for the individual isomers (B97-3c/def2-TZVP).

Index	Structure	Single Point Energy/Eh
1	*MM*	-5201.0614
2	*PP*	-5201.0429
3	*MP*	-5201.0743

<u>*MM*-Isomer</u>

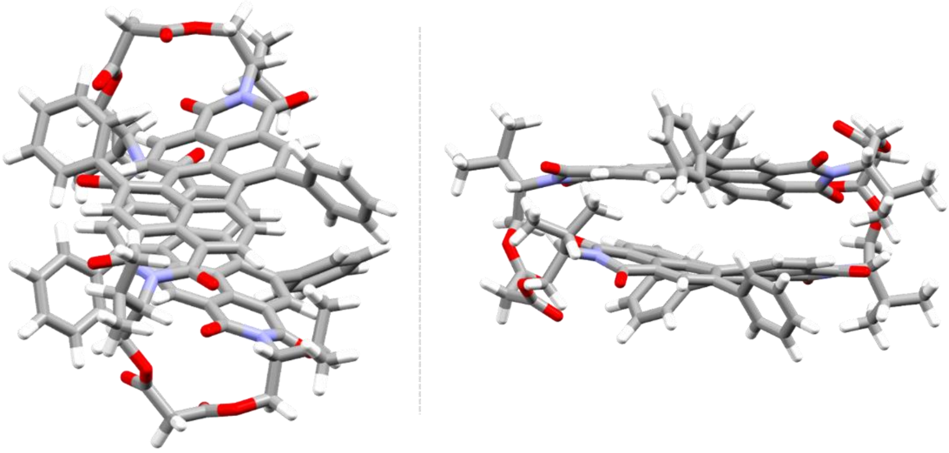

Supplementary Figure 6-5: DFT-optimised structure of the *MM*-isomer.

Supplementary Table 6-3: Calculated transitions (via transition electric dipole moments) of the *MM*-isomer (ωb97x/def2-SVP)

Transition	Wavelength/nm	Oscillator Strength	Rotary Strength
1	508.6	0.2666	-915.73448
2	484.1	1.0598	802.57072
3	387	0.0062	-9.8418
4	385.9	0.0048	10.76448
5	340.3	0.0872	-82.59823
6	339.4	0.0173	-64.6268
7	320.7	0.1948	-223.22084
8	317.3	0.2242	273.1278
9	315.5	0.0212	189.84476
10	313.3	0.0143	-12.0023

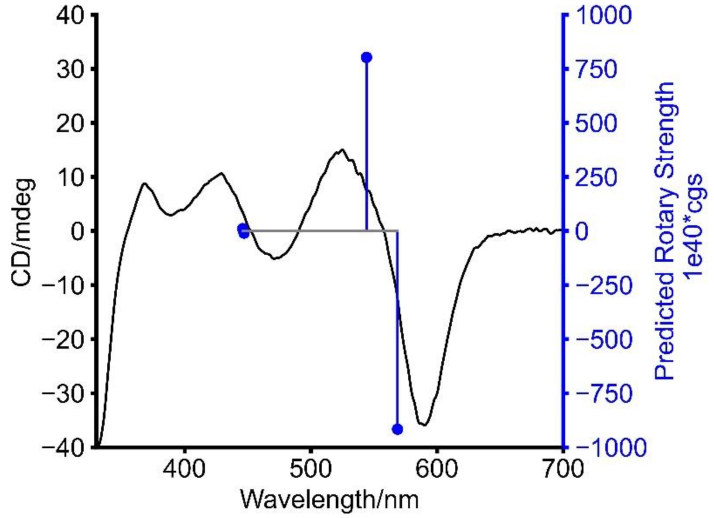

Supplementary Figure 6-6: Predicted CD spectrum of the *MM*-Isomer obtained by DFT. Spectrum was shifted by +60 nm to account for the blue-shift arising from the range-separated functional used and the reduction in π-conjugation from the modelling with only phenyl substituents.

MP-Isomer

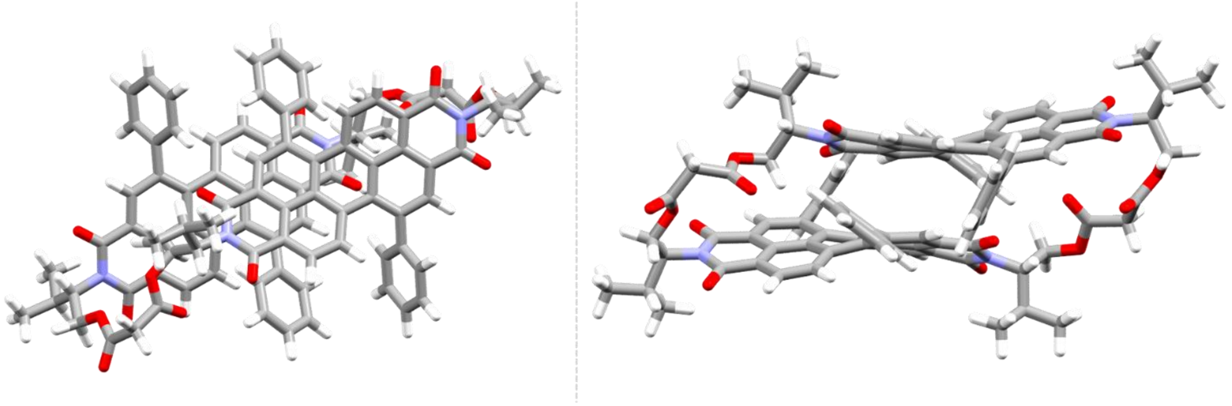

Supplementary Figure 6-7: DFT-optimised structure of the *MP*-isomer.

Supplementary Table 6-4: Calculated transitions (via transition electric dipole moments) of the *MP*-isomer (ωb97x/def2-SVP)

Transition	Wavelength/nm	Oscillator Strength	Rotary Strength
1	499.4	0.0413	-2.50763
2	492.7	1.5500	2.42161
3	352.3	0.0032	2.18707
4	347.4	0.0387	10.21830
5	337.5	0.0222	-79.14255
6	335.3	0.0171	114.31722
7	321.4	0.0092	-61.21918
8	318.6	0.3750	-25.81684
9	317.6	0.0448	-70.56259
10	316.2	0.0057	108.31817

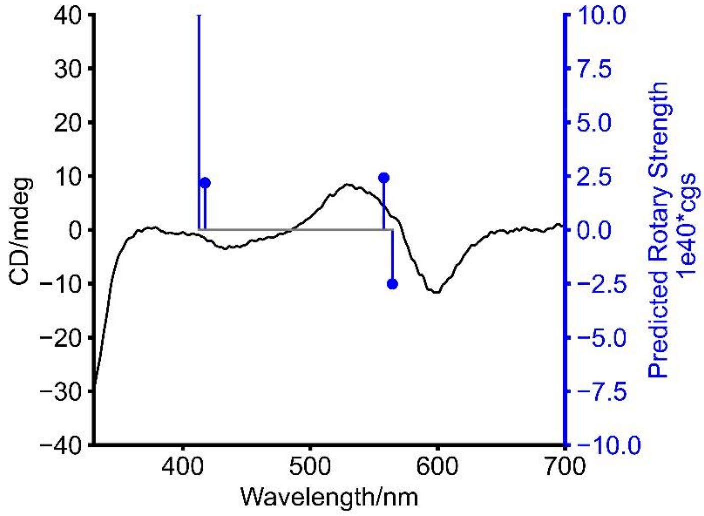

Supplementary Figure 6-8: Predicted CD spectrum of the *MP*-Isomer obtained by DFT. Spectrum was shifted by +65 nm to account for the blue-shift arising from the range-separated functional used and the reduction in π-conjugation from the modelling with only phenyl substituents

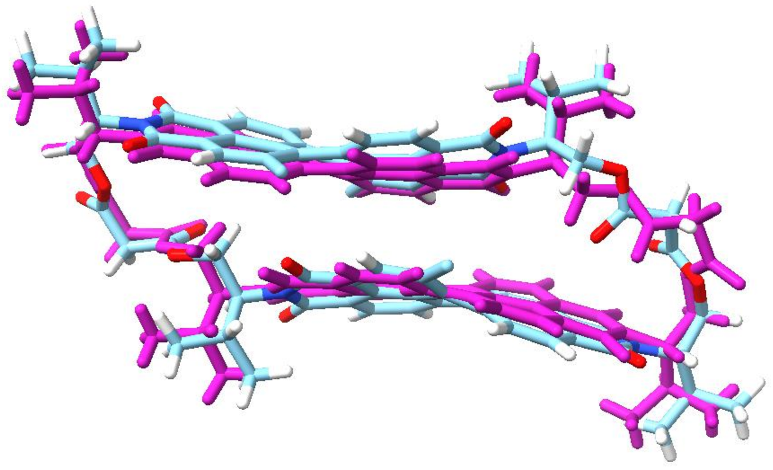

Supplementary Figure 6-9: Overlay of the CREST-predicted and DFT-optimised structure (cyan) with the structure obtained from single-crystal XRD (purple). Aryl-groups were omitted for clarity.

7) NMR Spectra

L-Valinol-Perylene Diimide-1,7-Dibromide 1

^{1}H NMR (400 MHz, $CDCl_3$)

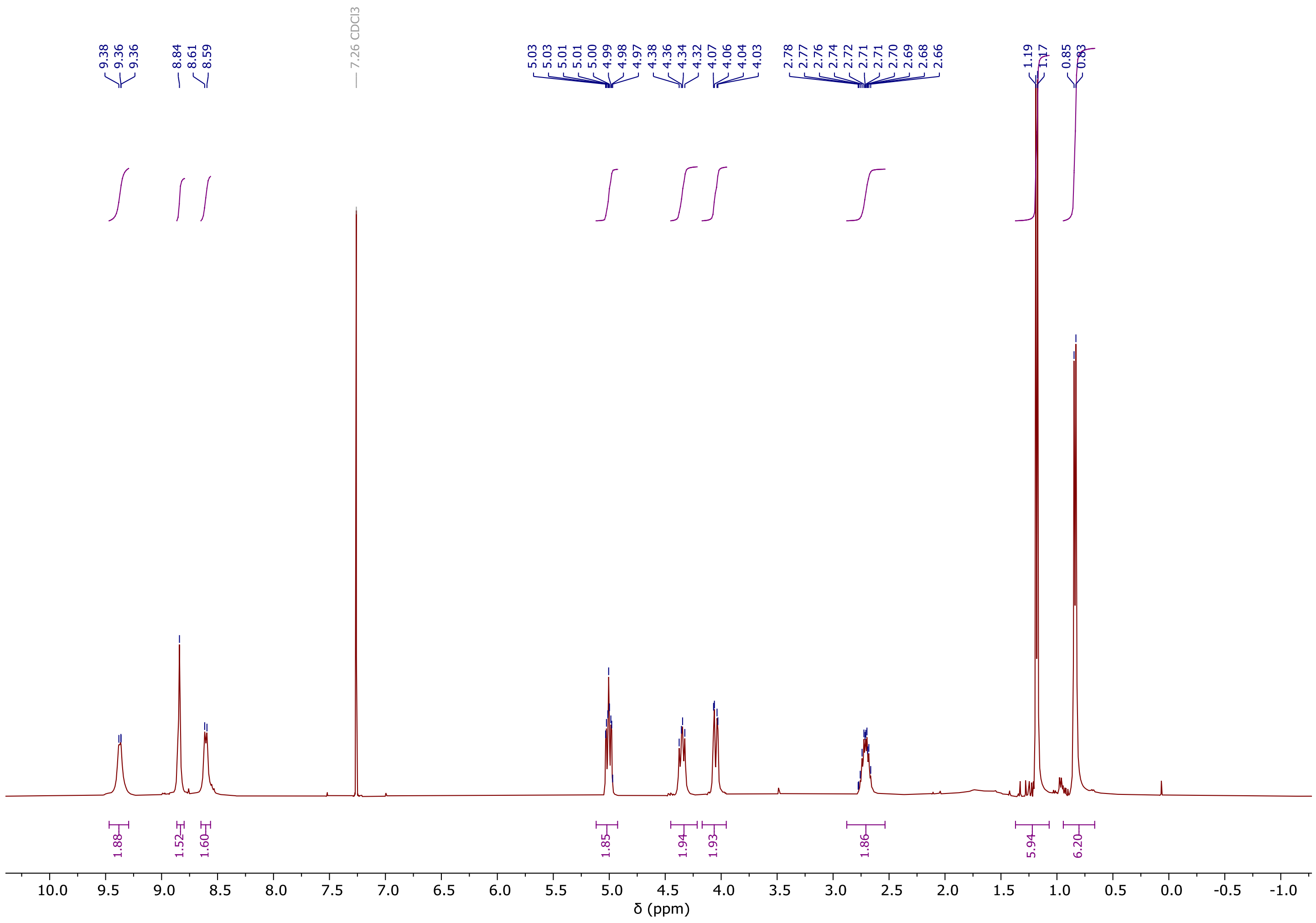

^{13}C NMR (101 MHz, $CDCl_3$)

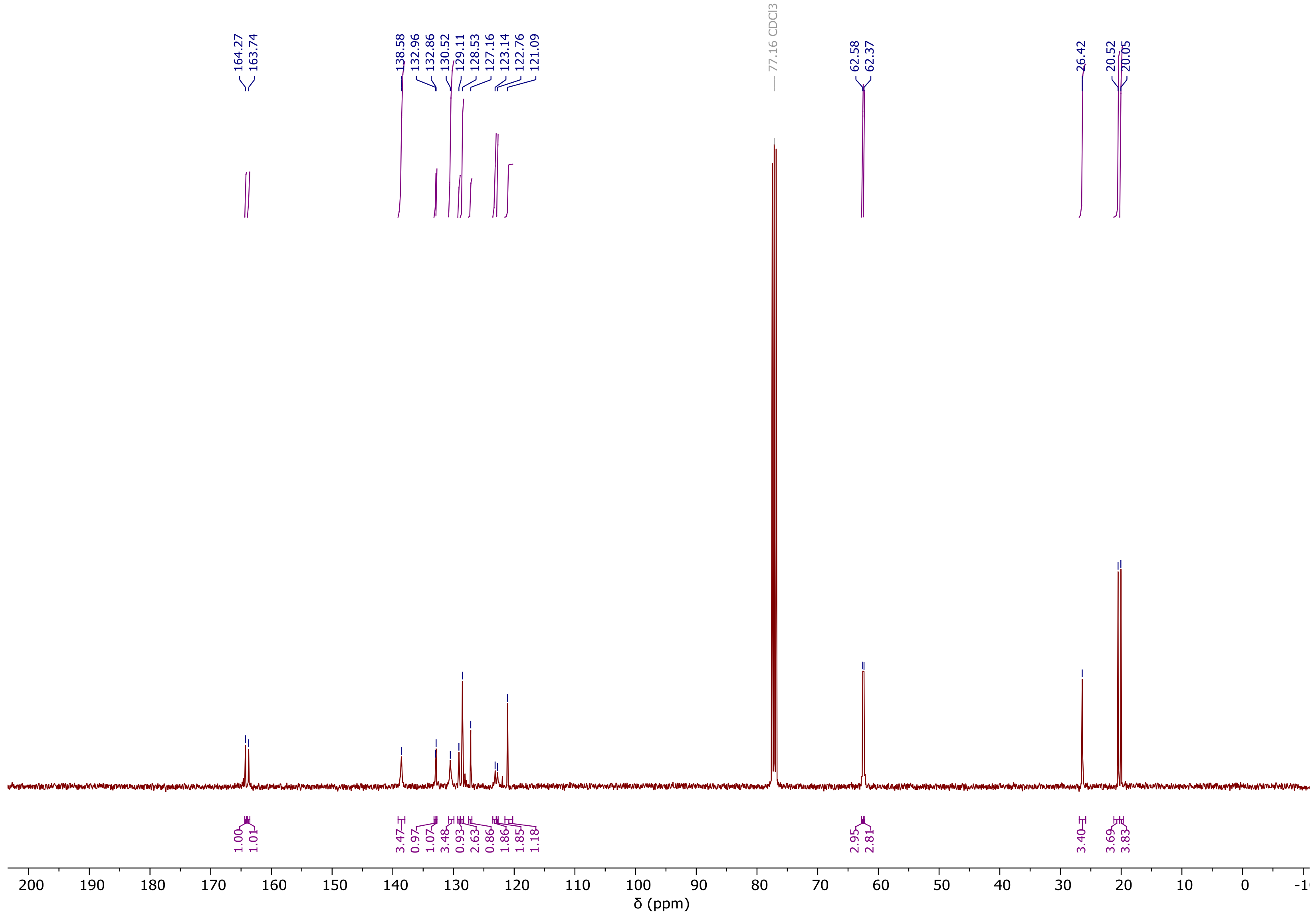

TBS-O-L-Valinol PDI Dibromide 2

^{1}H NMR (400 MHz, $CDCl_3$)

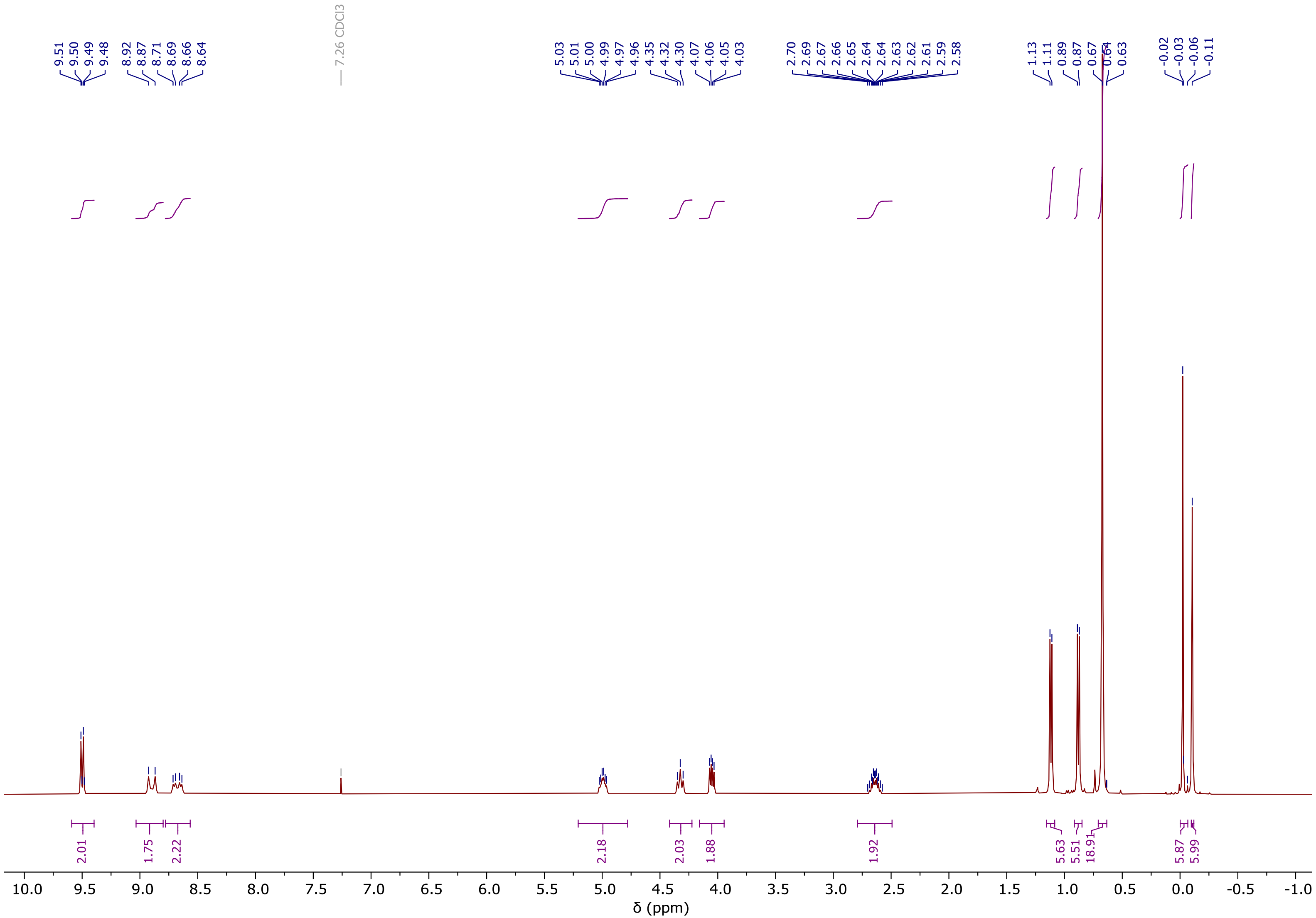

^{13}C NMR (101 MHz, $CDCl_3$)

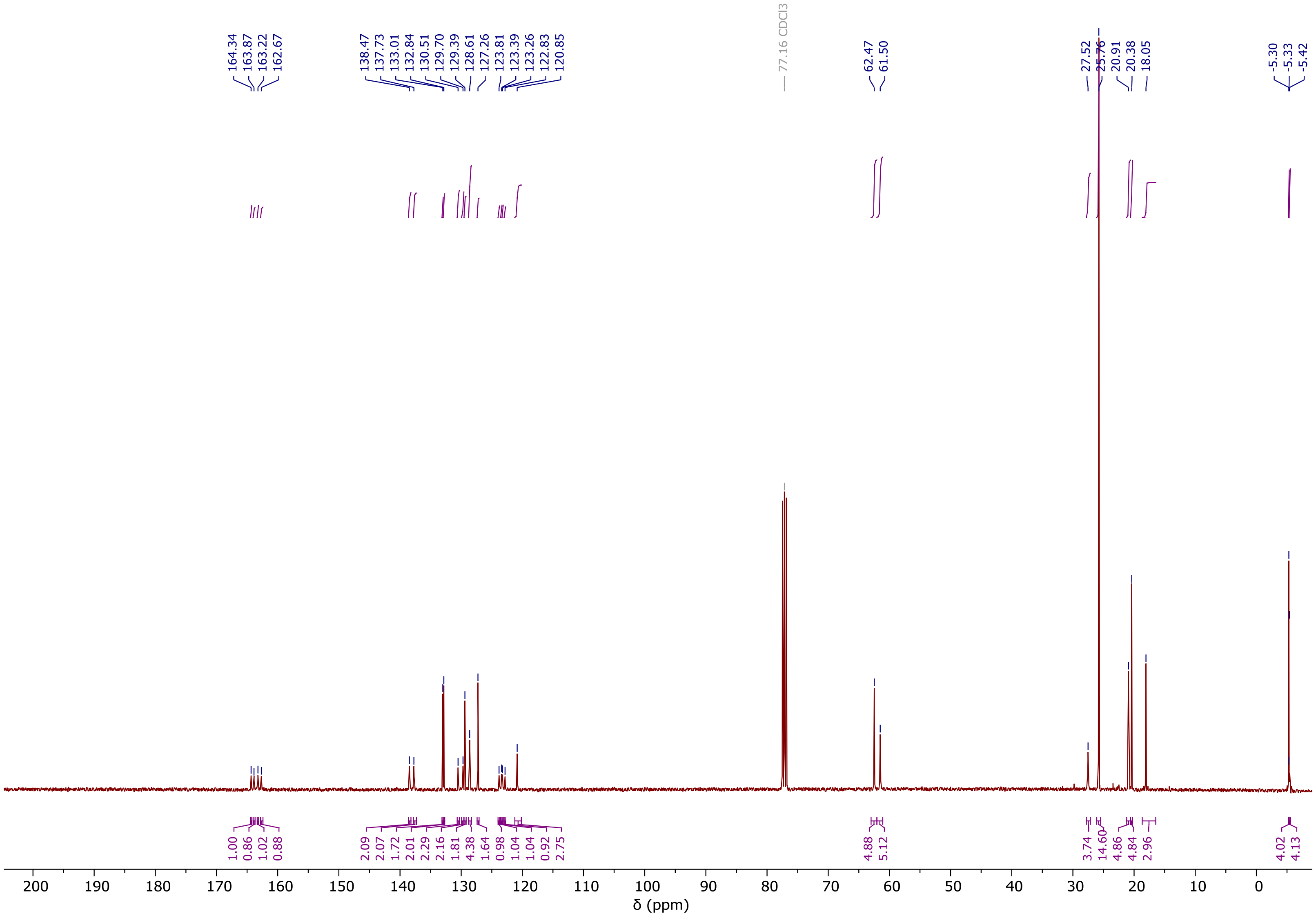

TBS-O-bis(phenyl)-L-Valinol PDI 3

^{1}H NMR (400 MHz, $CDCl_3$)

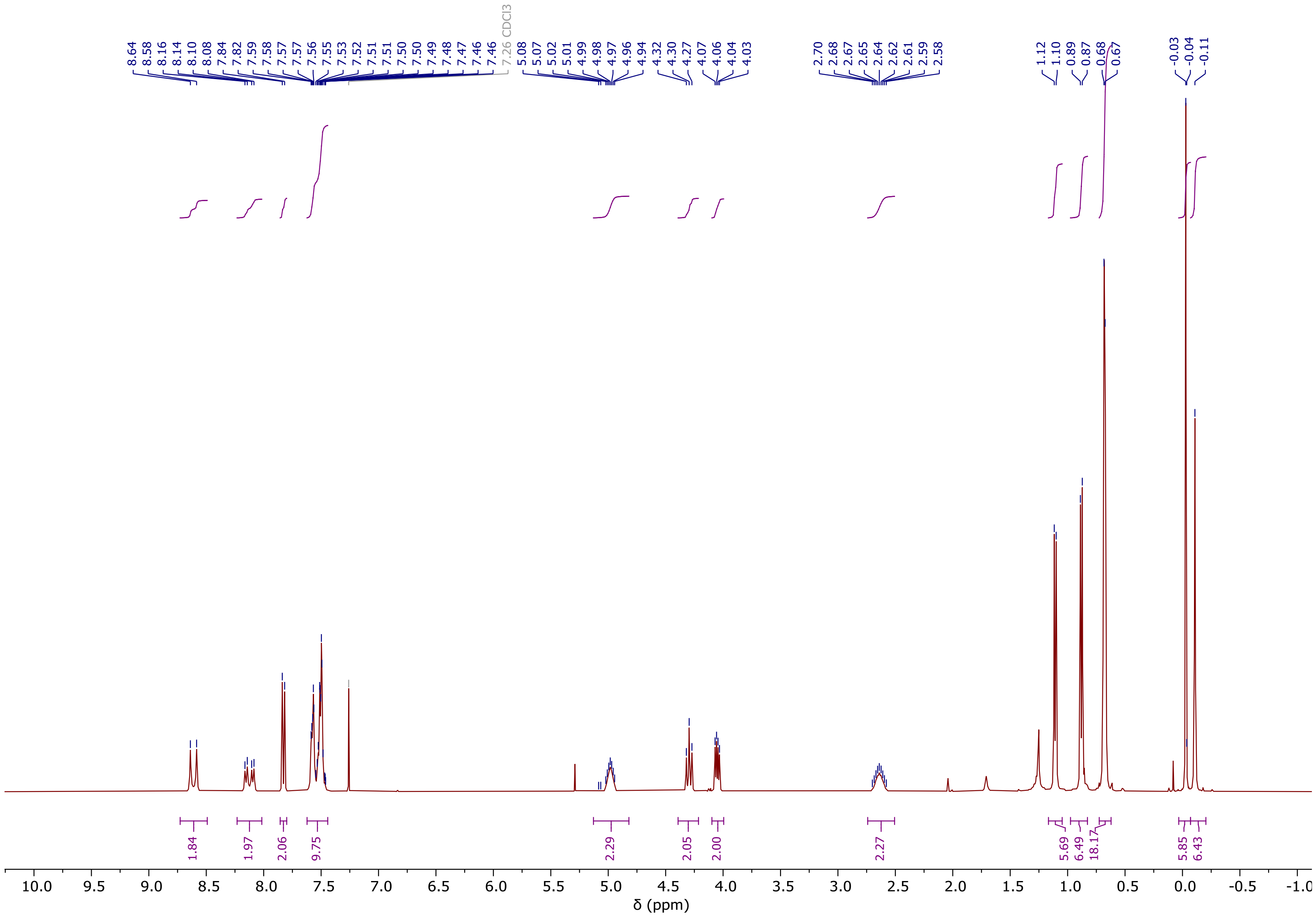

^{13}C NMR (101 MHz, $CDCl_3$)

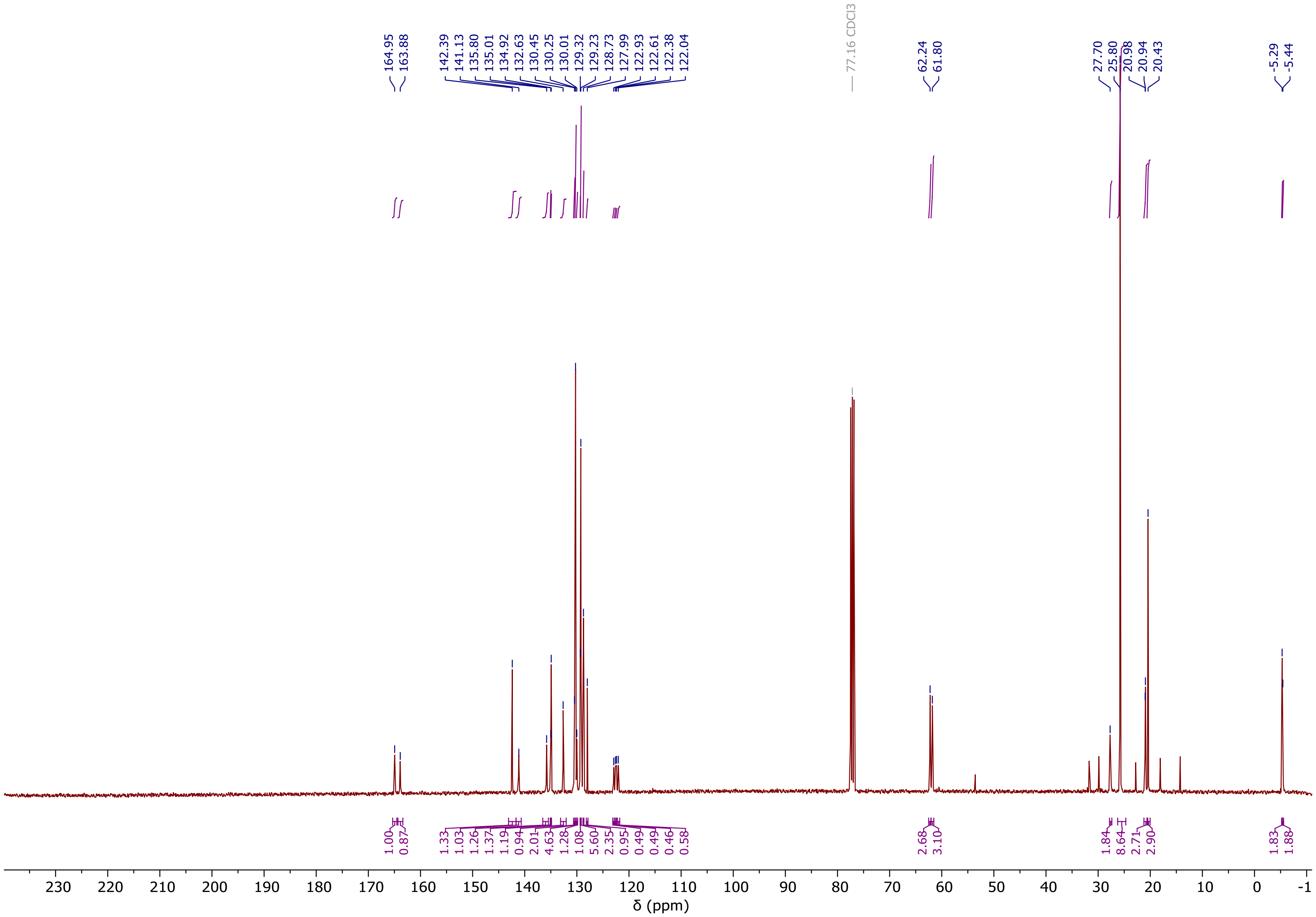

TBS-O-bis(phenyl)-L-Valinol PDI 4

^{1}H NMR (400 MHz, TCE-d_2, 373 K)

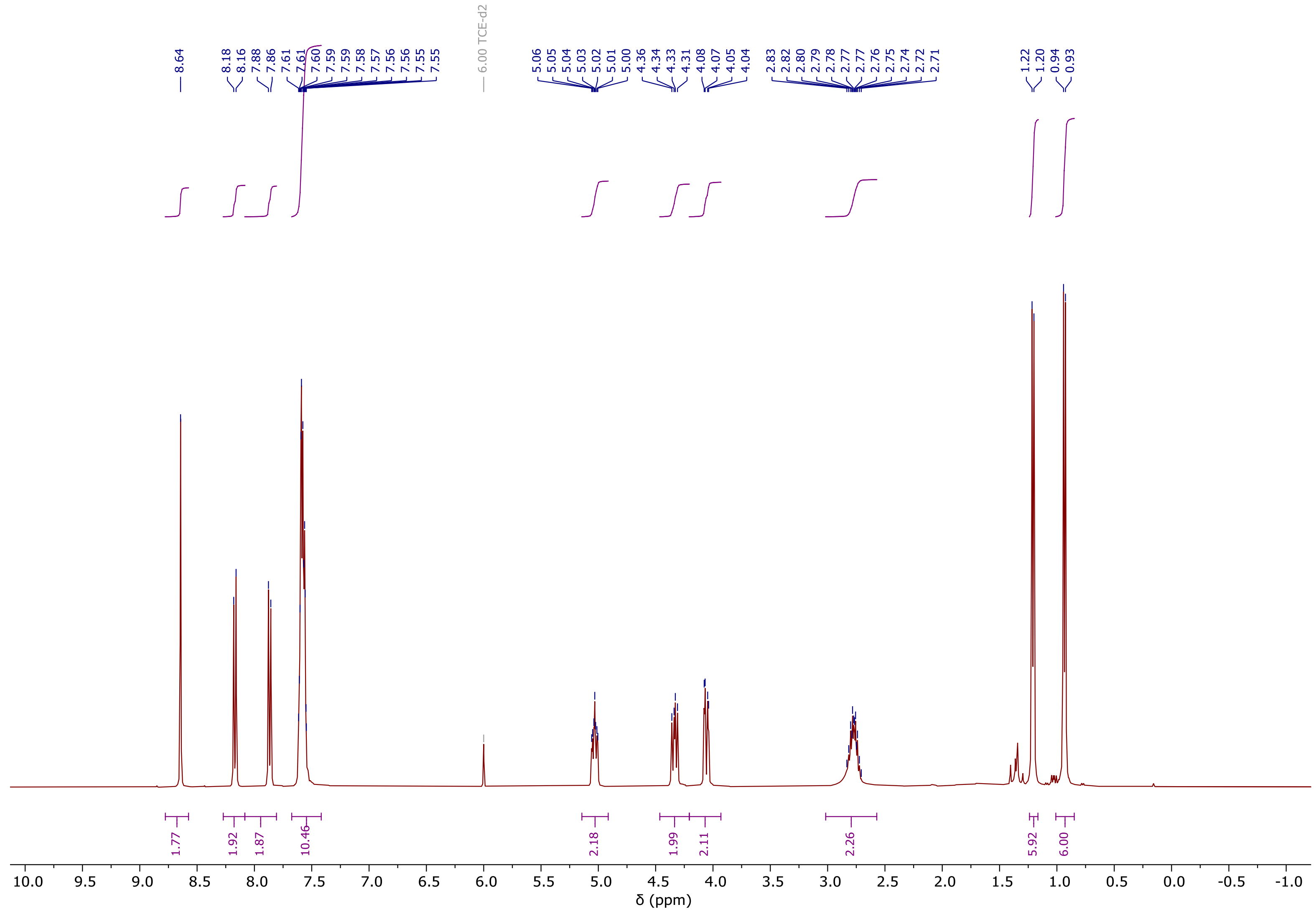

^{13}C NMR (101 MHz, TCE-d_2, 298 K)

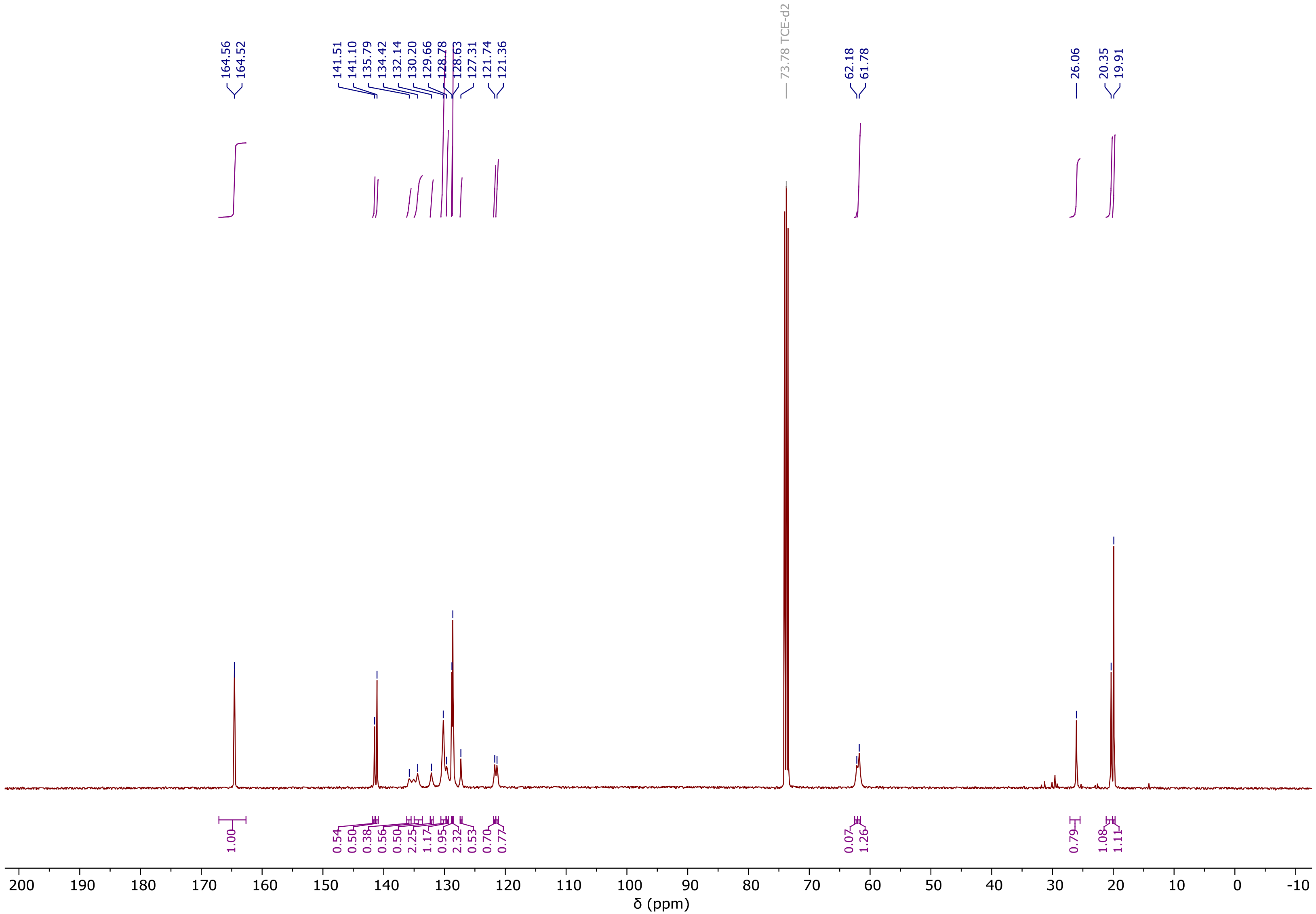

Bis(phenyl)-L-Valinol PDI Macrocycle 5

^{1}H NMR (400 MHz, 373 K, TCE-d_2)

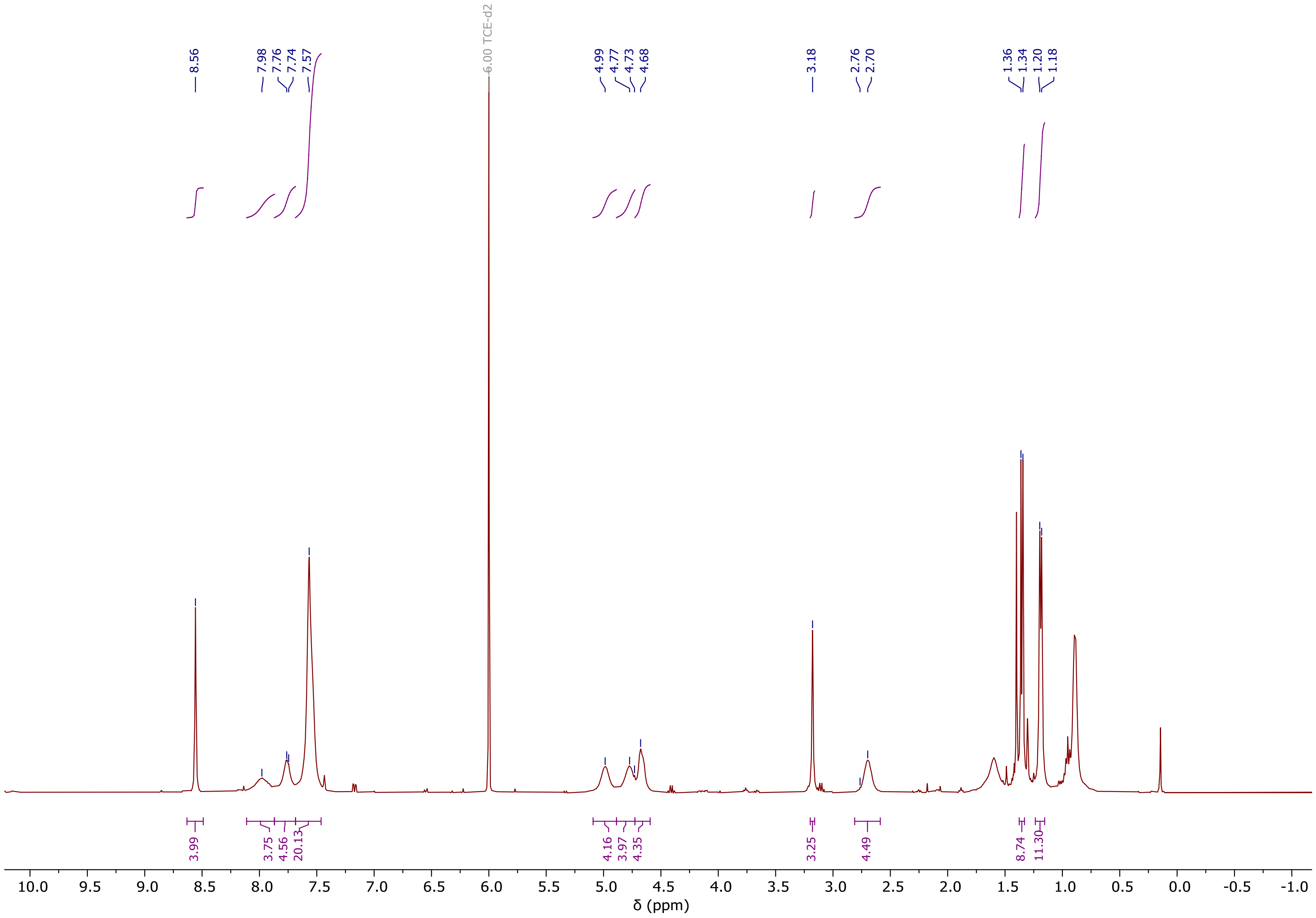

^{13}C NMR (101 MHz, 373 K, TCE-d_2)

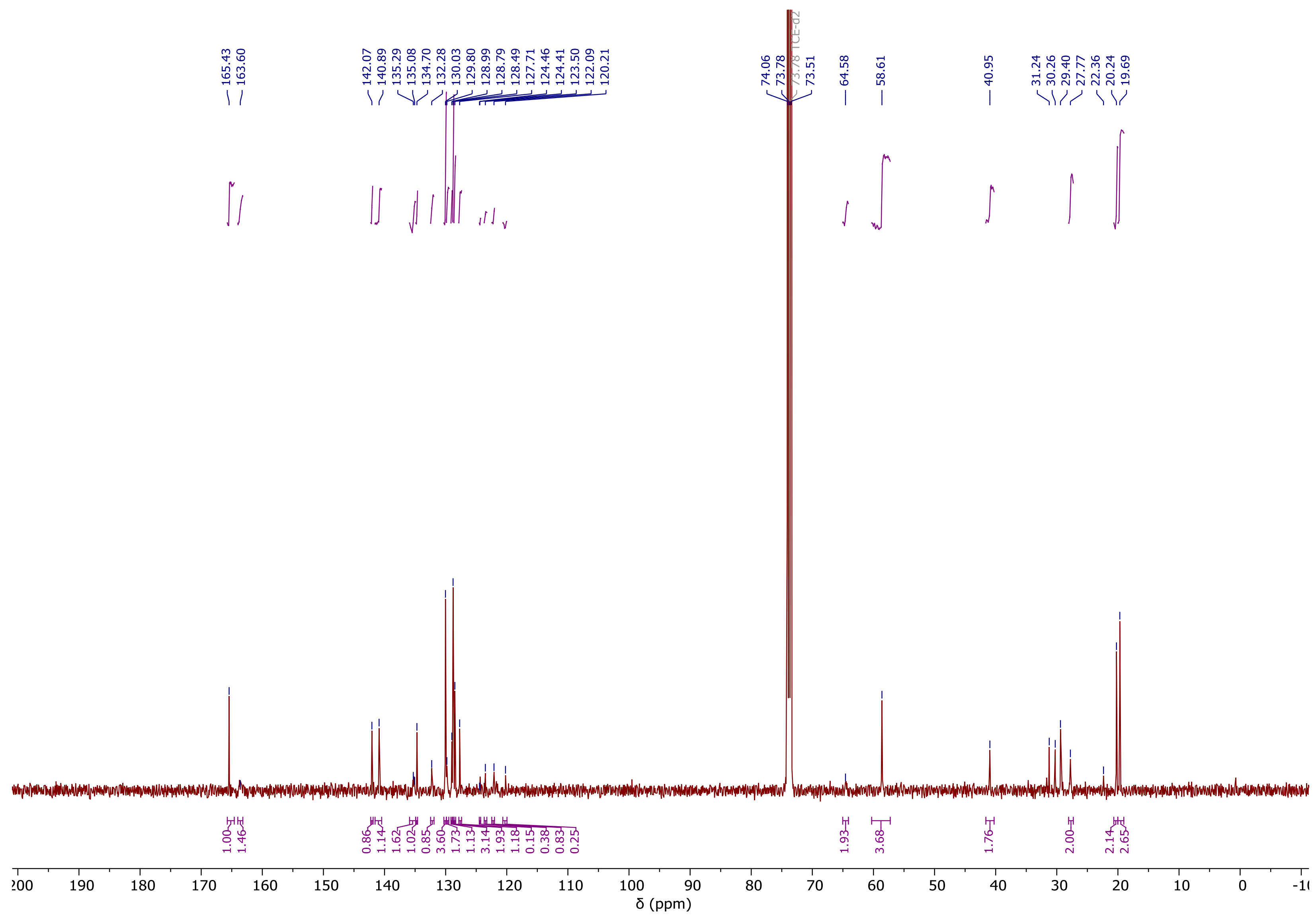

TBS-O-bis(terphenyl)-L-Valinol PDI 6

^{1}H NMR (400 MHz, $CDCl_3$)

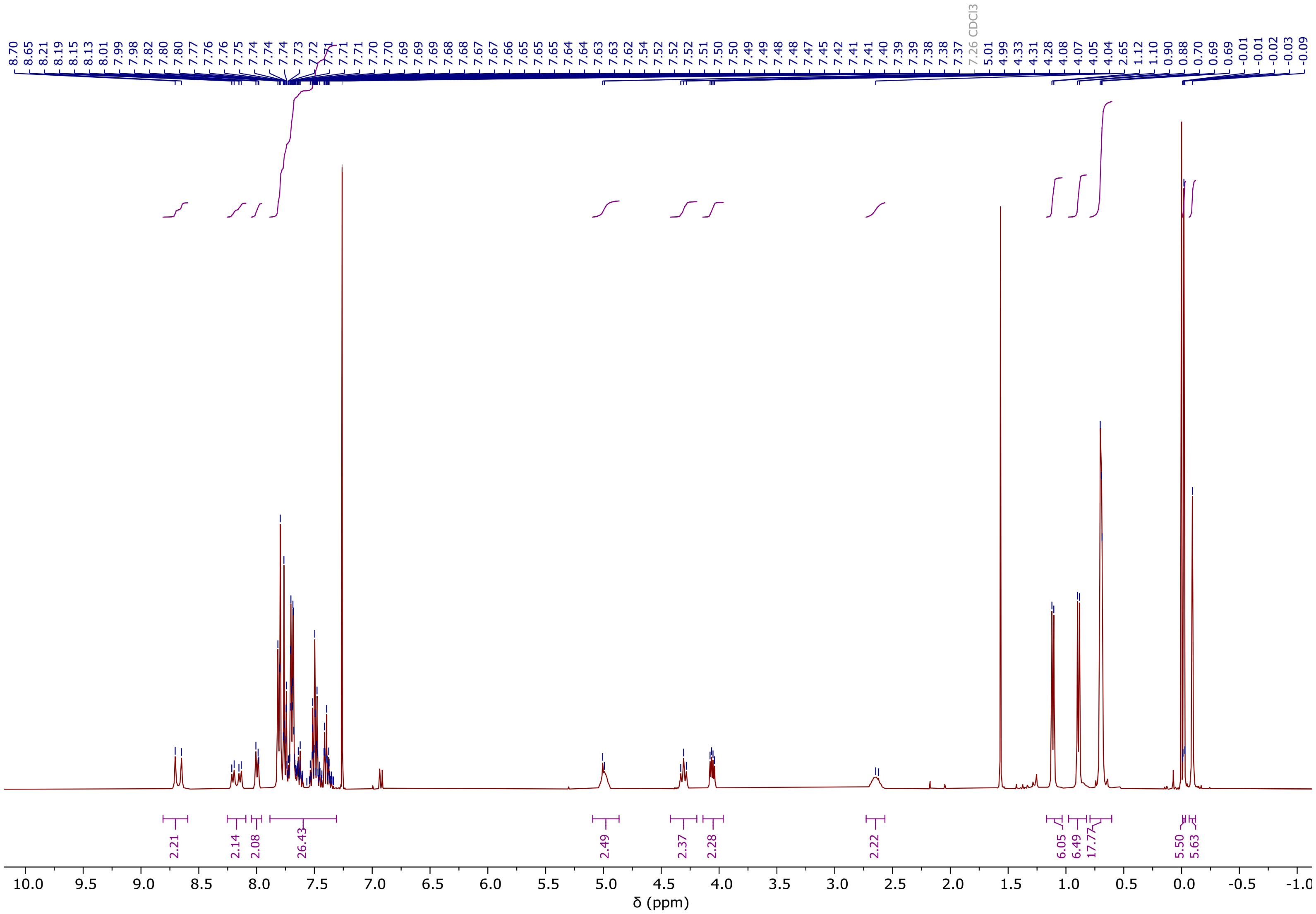

^{13}C NMR (101 MHz, $CDCl_3$)

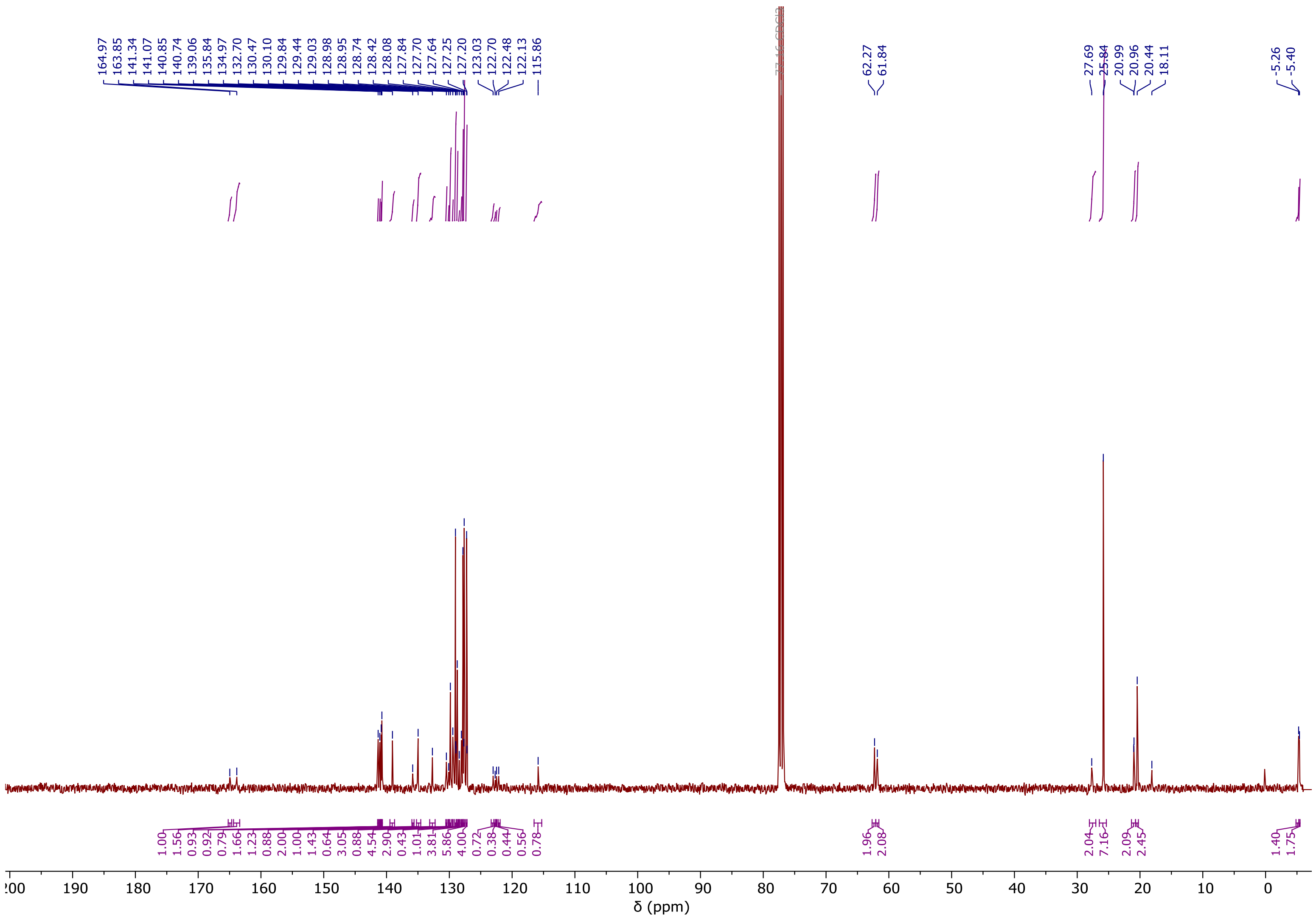

OH-bis(terphenyl)-L-Valinol PDI 7

^{1}H NMR (400 MHz, TCE-d_2, 373 K)

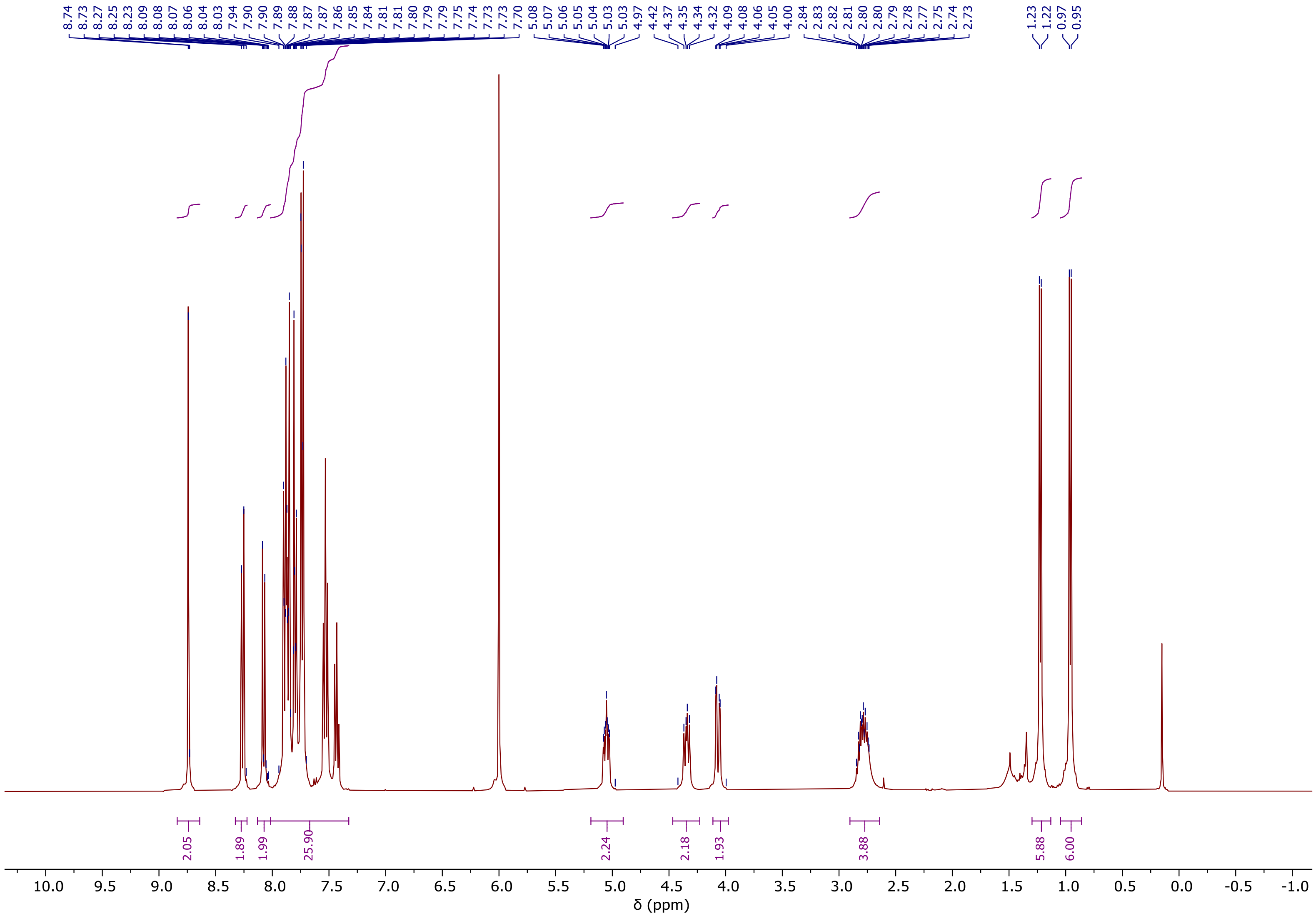

^{13}C NMR (101 MHz, TCE-d_2, 298 K)

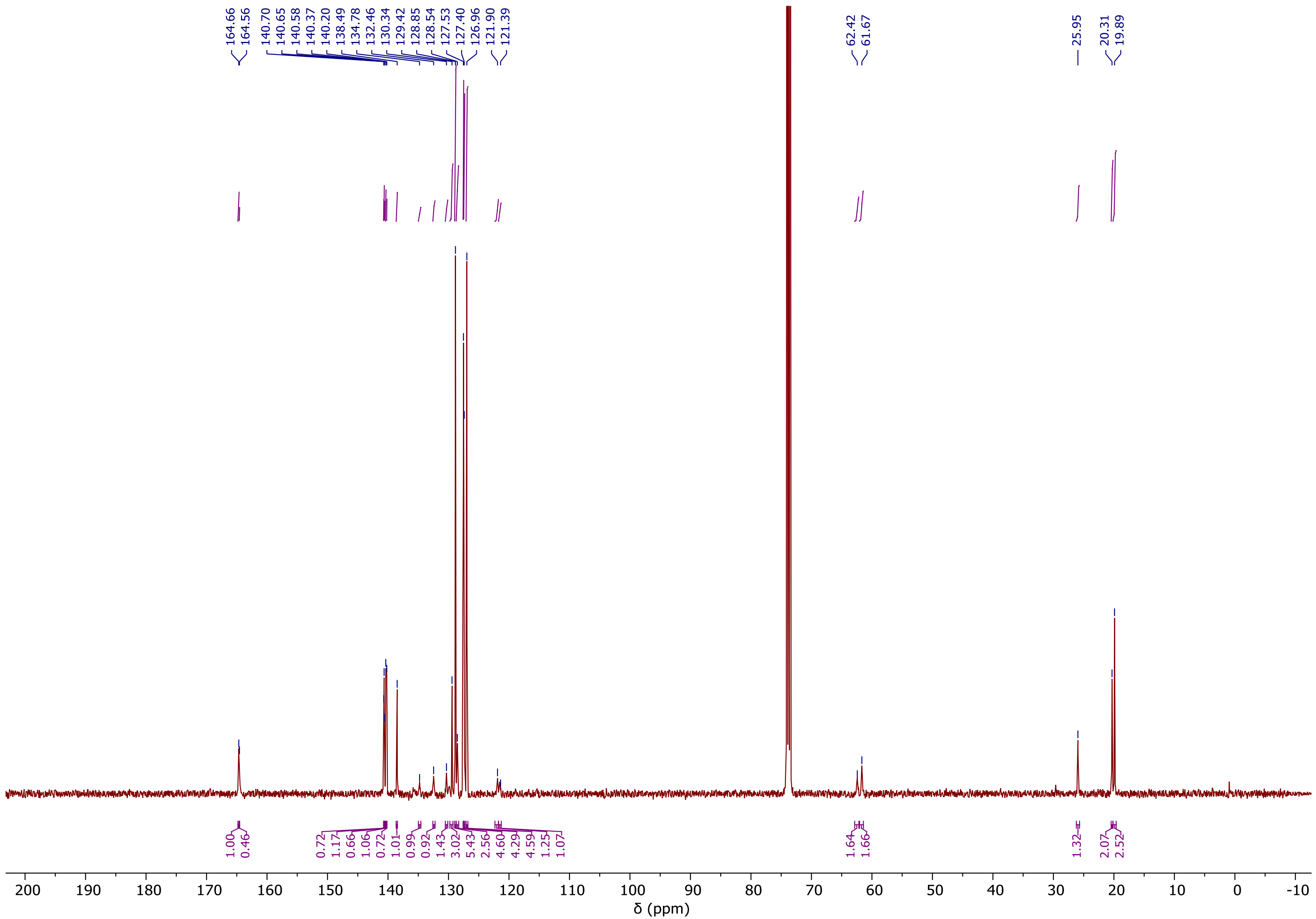

Bis(terphenyl)-L-Valinol PDI Macrocycle (MM Isomer) 8a

^{1}H NMR (400 MHz, $CDCl_3$)

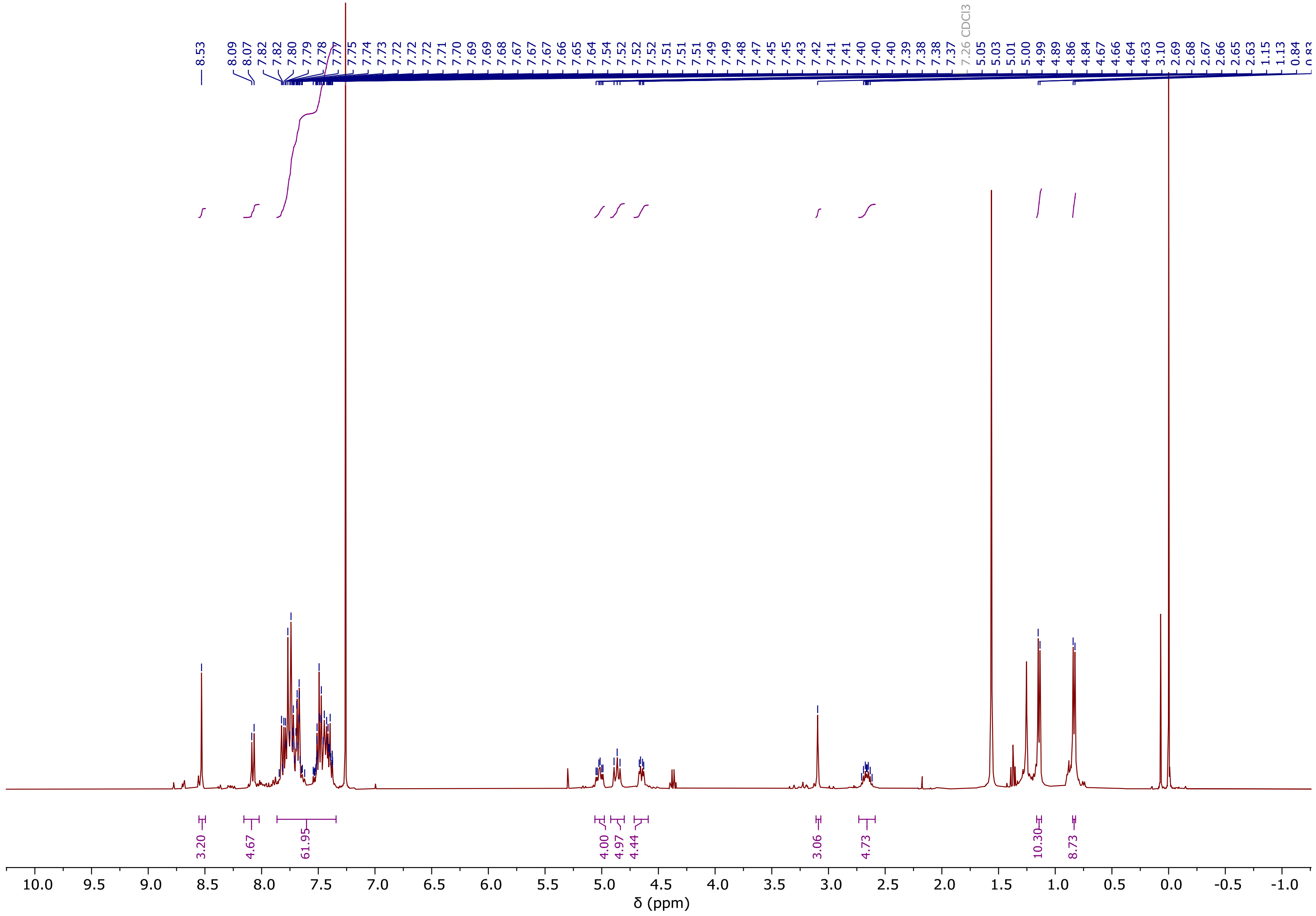

^{13}C NMR (101 MHz, $CDCl_3$)

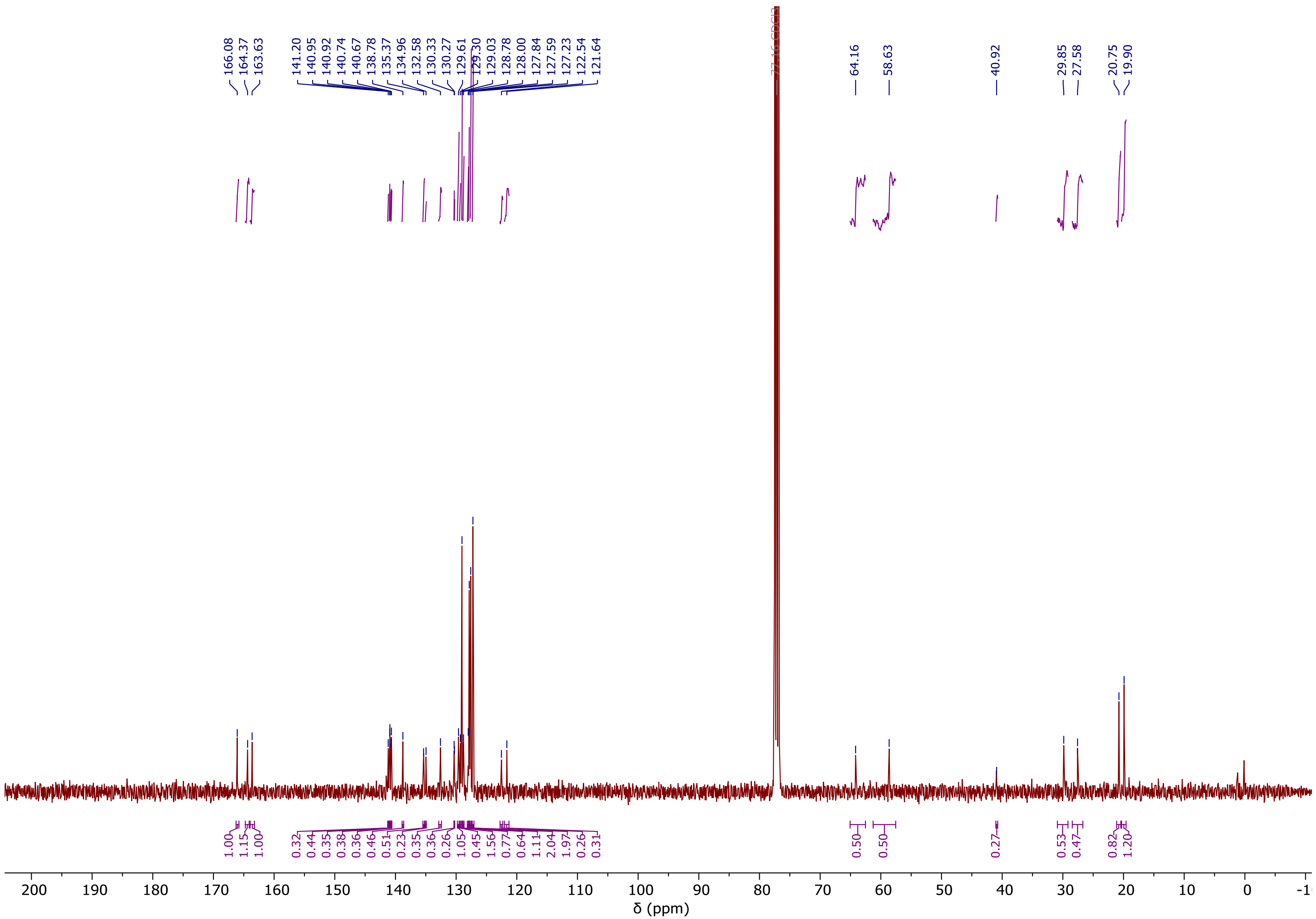

Bis(terphenyl)-L-Valinol PDI Macrocycle (PP Isomer) 8b

^{1}H NMR (400 MHz, $CDCl_3$)

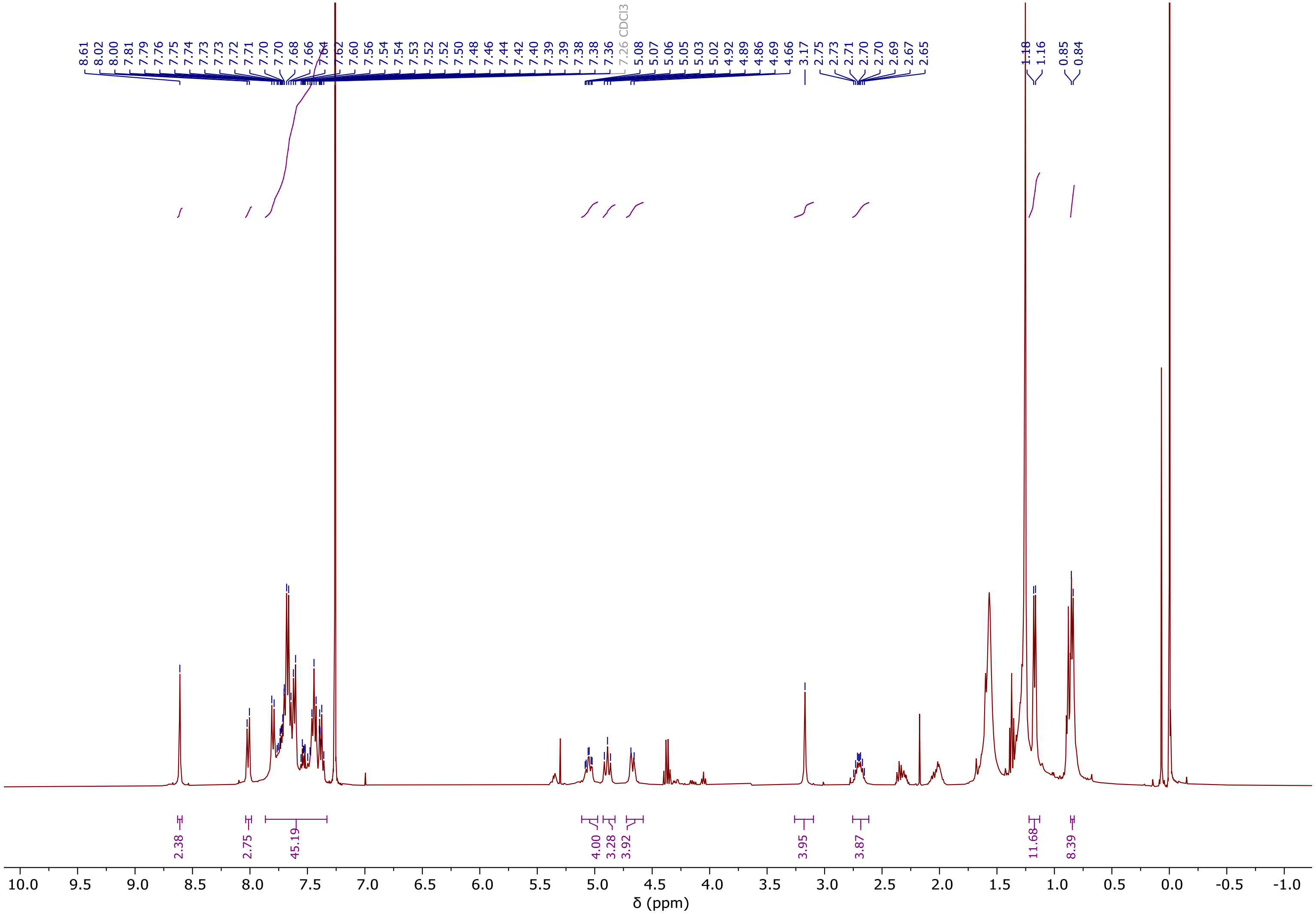

^{13}C NMR (101 MHz, $CDCl_3$)

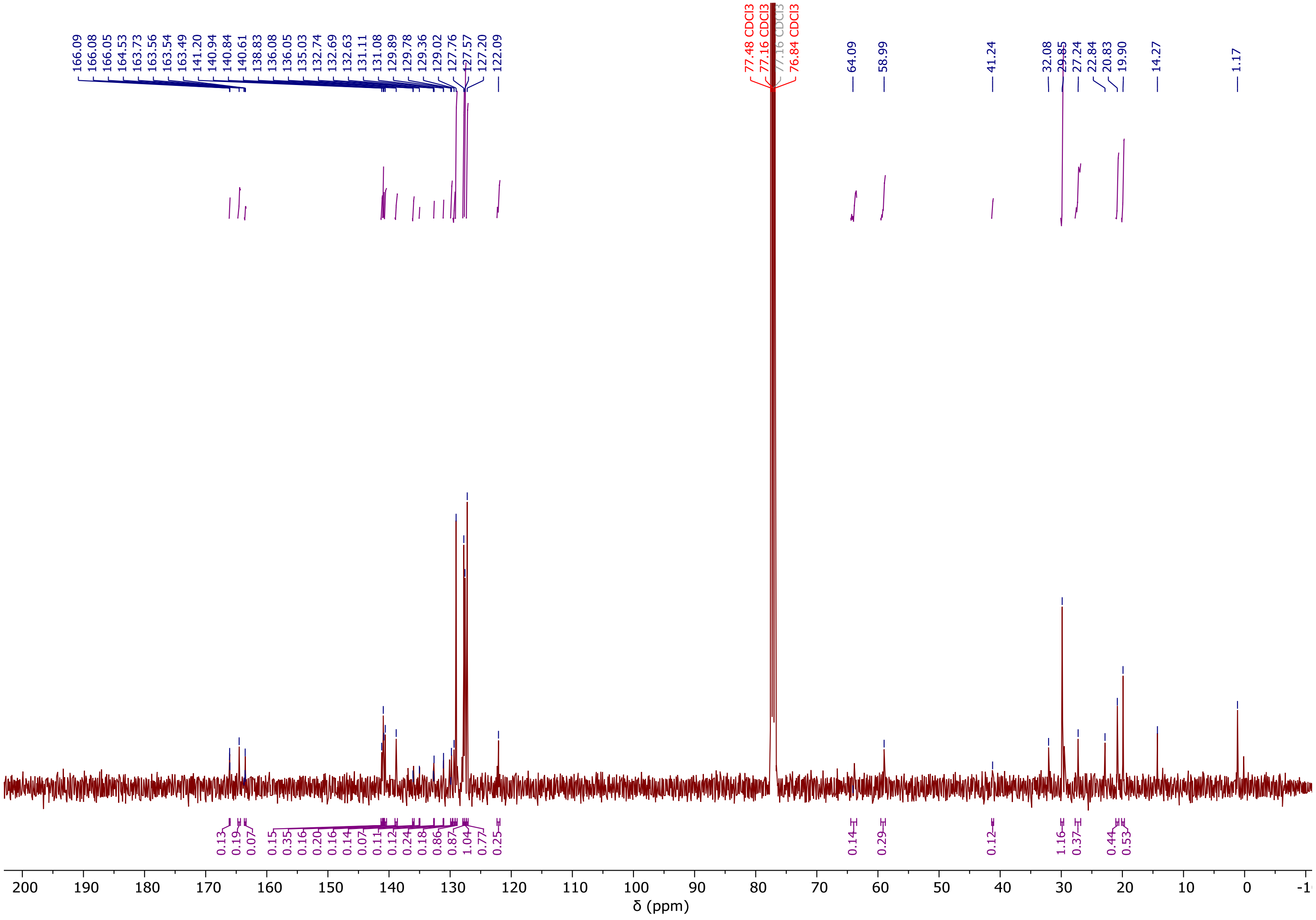

Bis(terphenyl)-L-Valinol PDI Macrocycle (MP Isomer) 8c

^{1}H NMR (400 MHz, TCE-d_2)

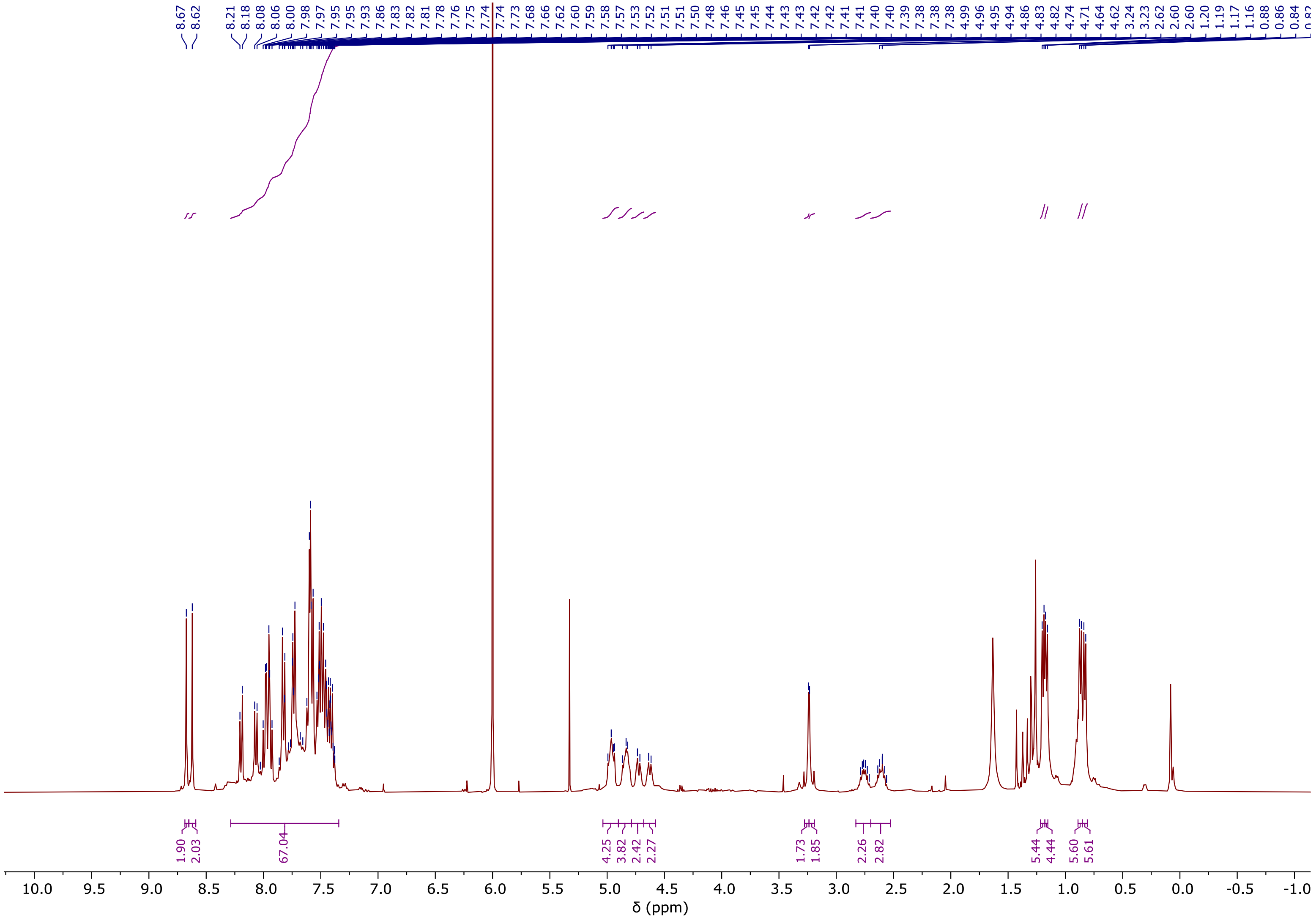

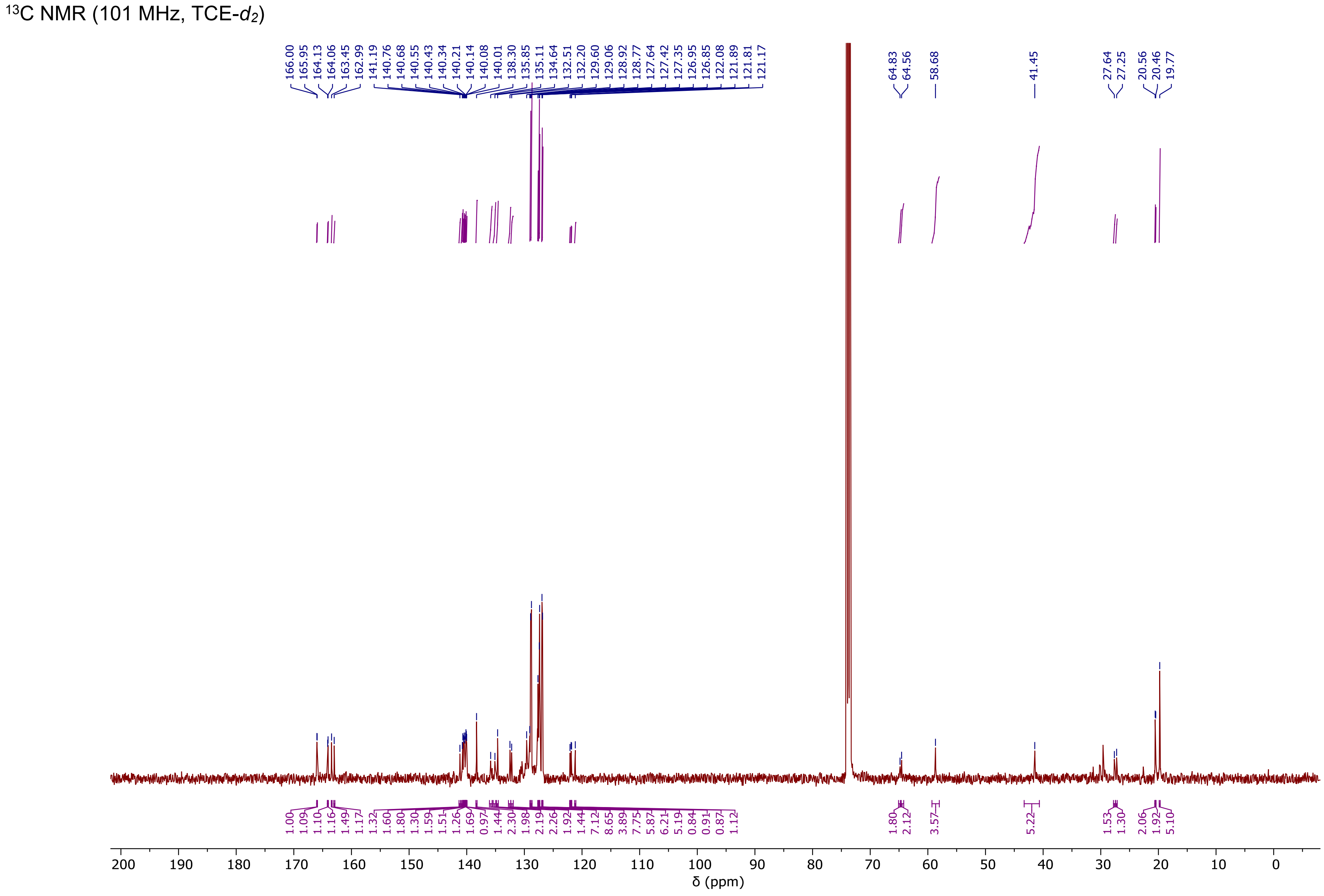

13C NMR (101 MHz, TCE-d2)
166.00
165.95
164.13
164.06
163.45
162.99
141.19
140.76
140.68
140.55
140.43
140.34
140.21
140.14
140.08
140.01
138.30
135.85
135.11
134.64
132.51
132.20
129.60
129.06
128.92
128.77
127.64
127.42
127.35
126.95
126.85
122.08
121.89
121.81
121.17
64.83
64.56
58.68
41.45
27.64
27.25
20.56
20.46
19.77
δ (ppm)

^{1}H NMR (400 MHz, $CDCl_3$)

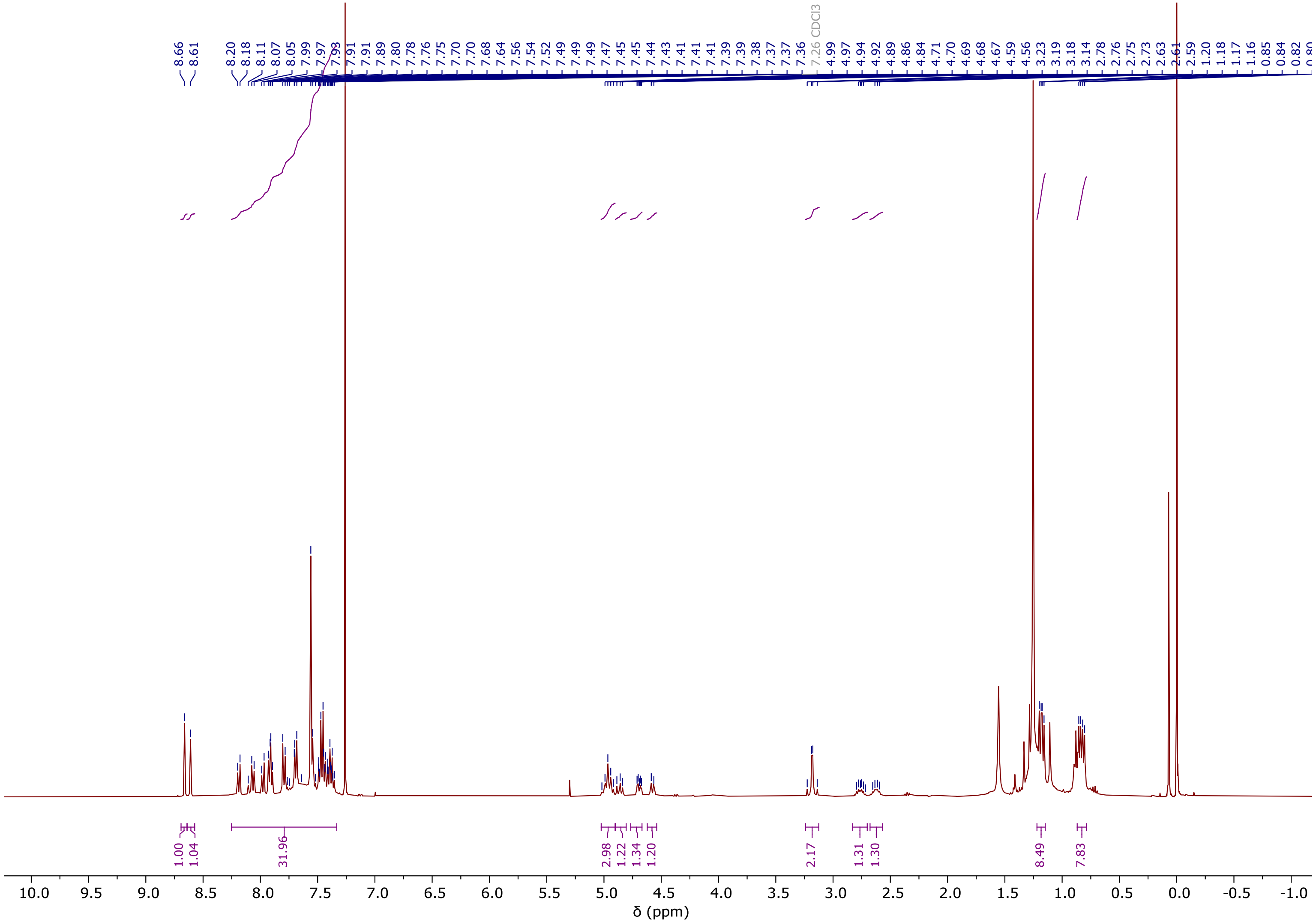

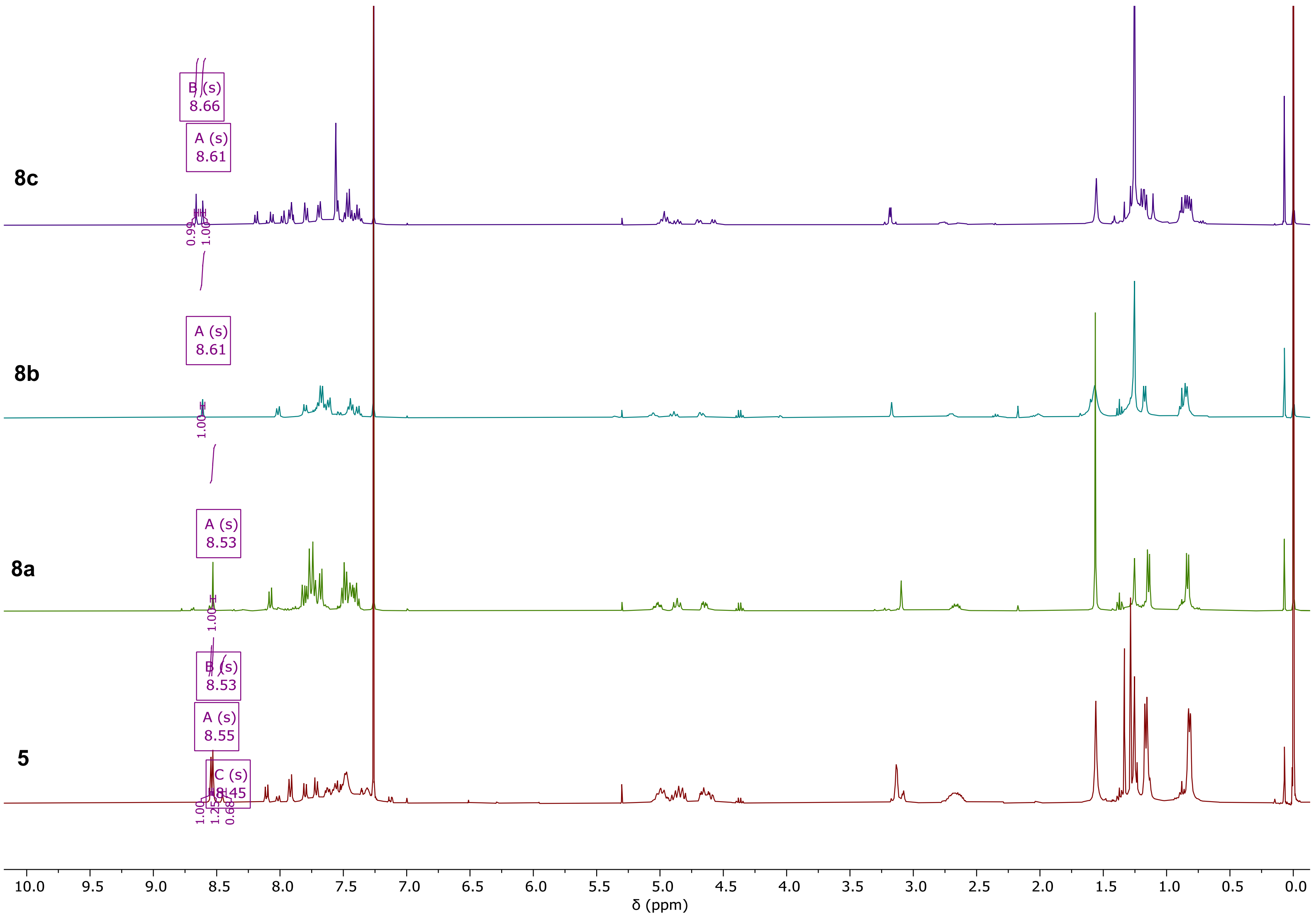

Supplementary Figure 7-1: Comparison of Spectra of **5** and **8a-c** in $CDCl_3$.

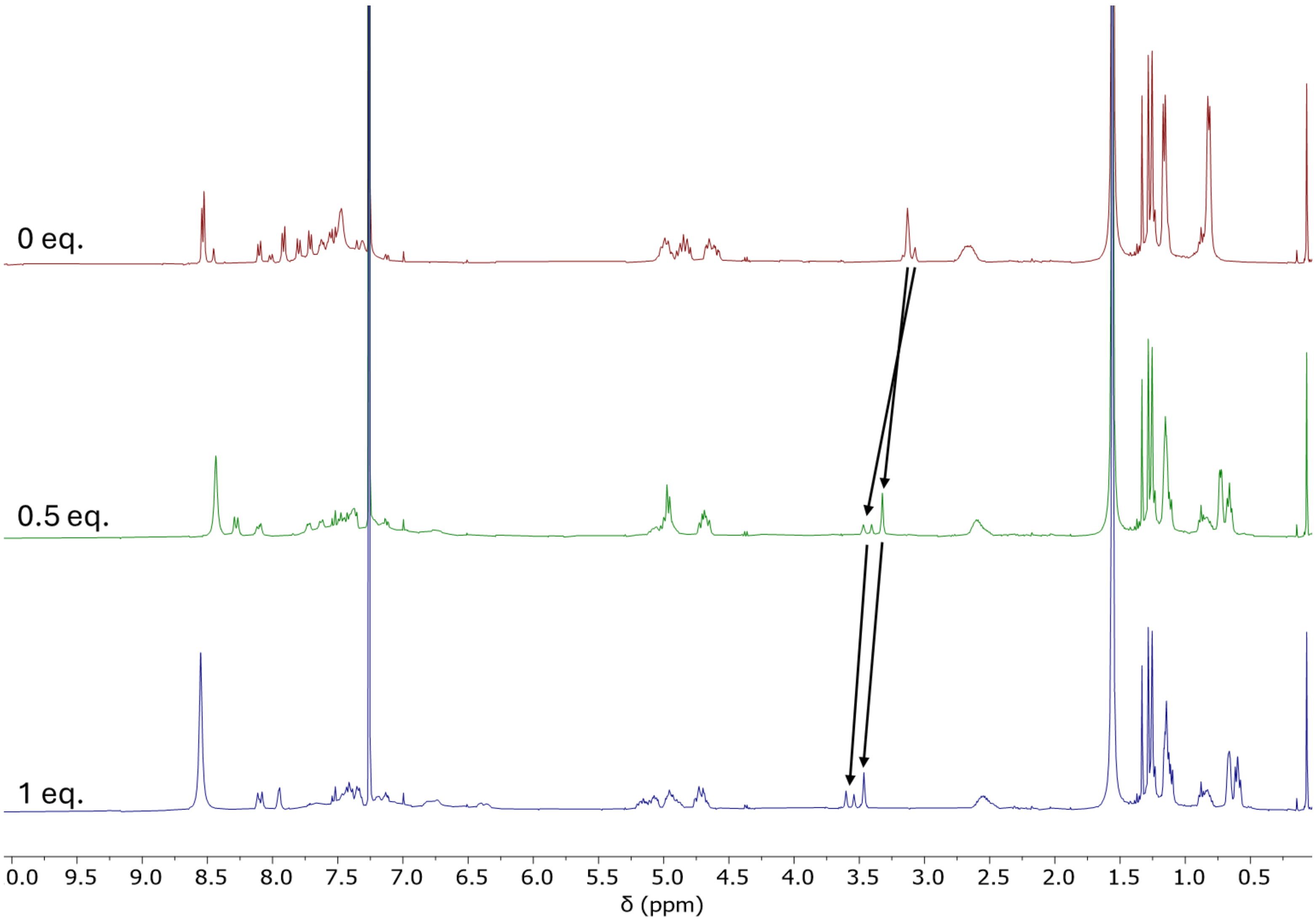

Supplementary Figure 7-2: NMR spectrum of macrocycle **5** in $CDCl_3$ (1.6 mM) upon addition of coronene (0.5 and 1 equivalents) (**Supplementary Figure 4-18**). The deshielding of the protons corresponding to the malonate-CH_2 (black arrows) indicates binding of the coronene inside the macrocycle cavity.

8) Variable Temperature NMR

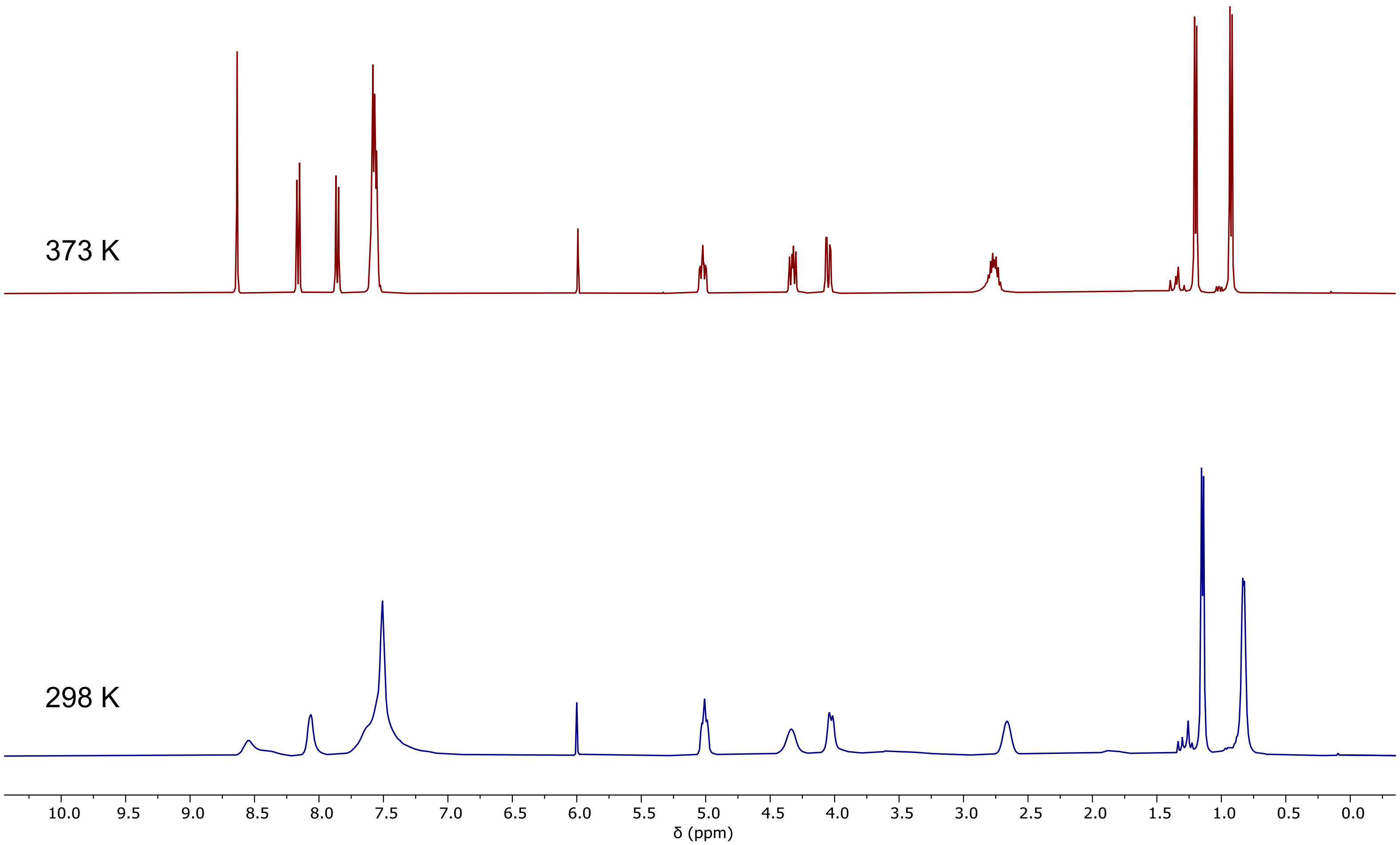

Supplementary Figure 8-1: Low and high-temperature NMR of Ph-substituted Diol-PDI **4** (TCE-d_2)

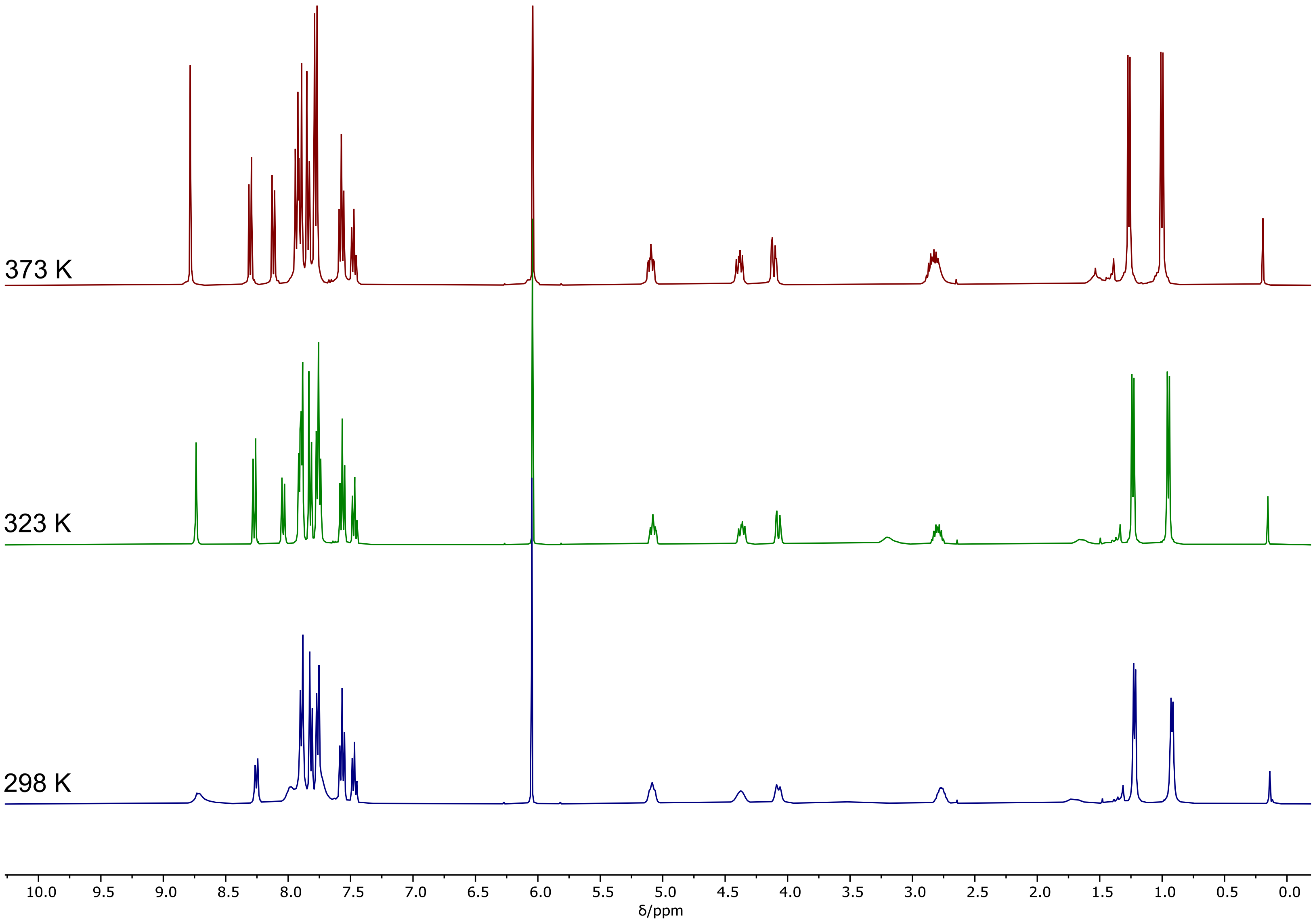

Supplementary Figure 8-2: Variable Temperature NMR of Terphenyl-Diol PDI **7** in TCE-d_2

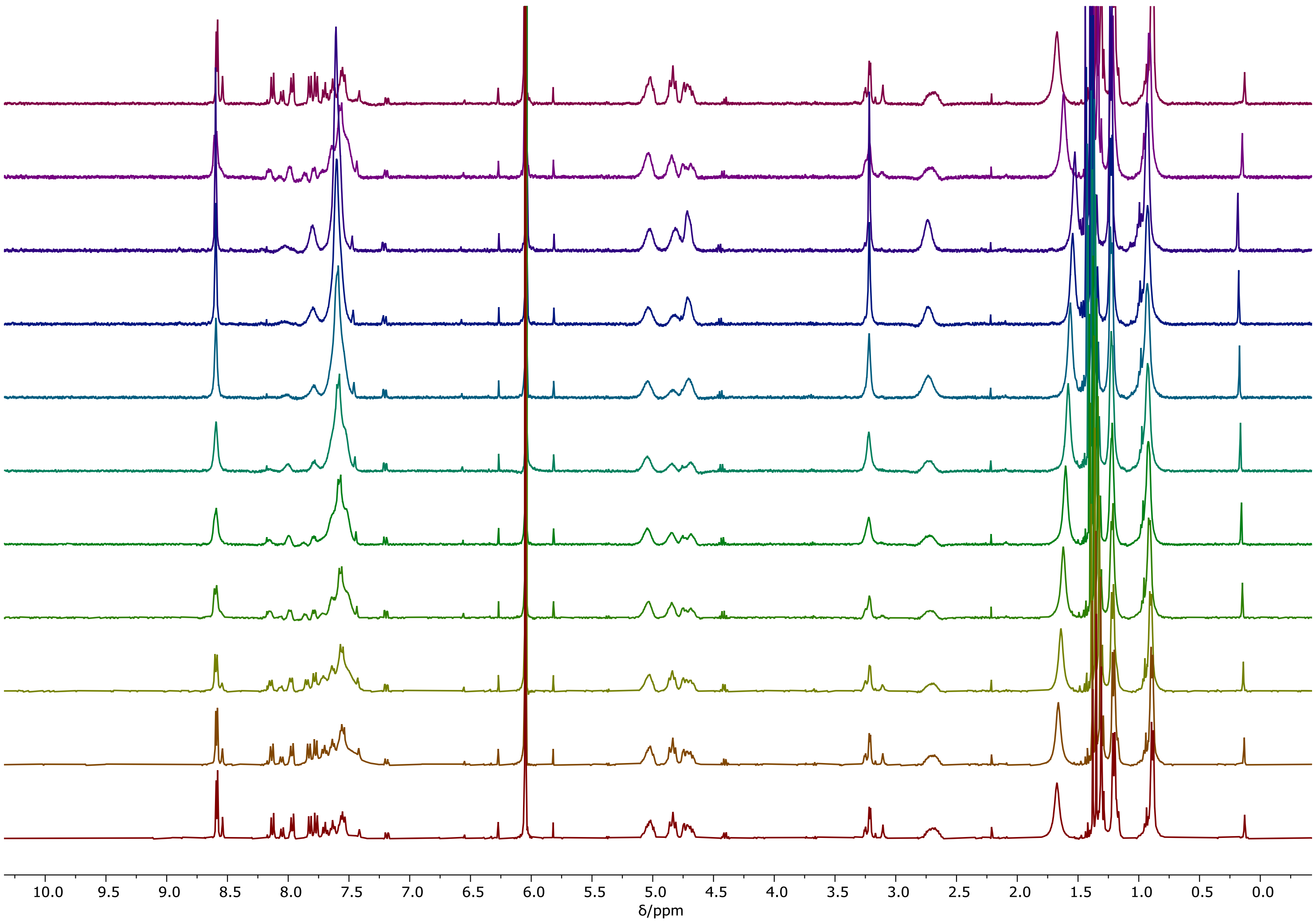

Supplementary Figure 8-3: Variable Temperature NMR of Ph-substituted macrocycle **5** in TCE-d_2.

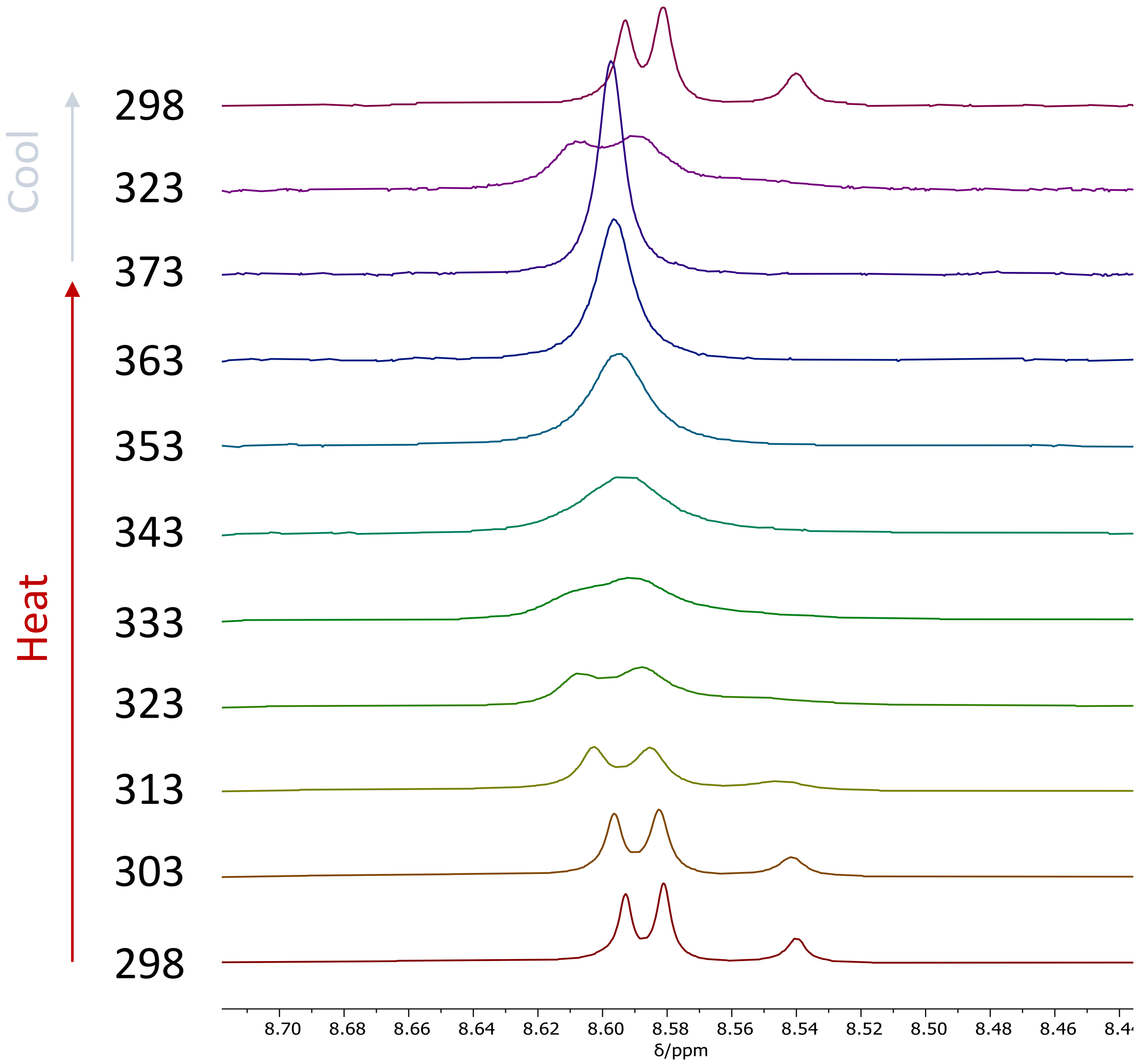

Supplementary Figure 8-4: Zoom on the PDI aromatic protons of a variable temperature NMR of Ph-substituted macrocycle **5** in TCE-d_2. Data allowed for an estimation of the inversion barrier between isomers of ΔG = 78 kJ/mol.

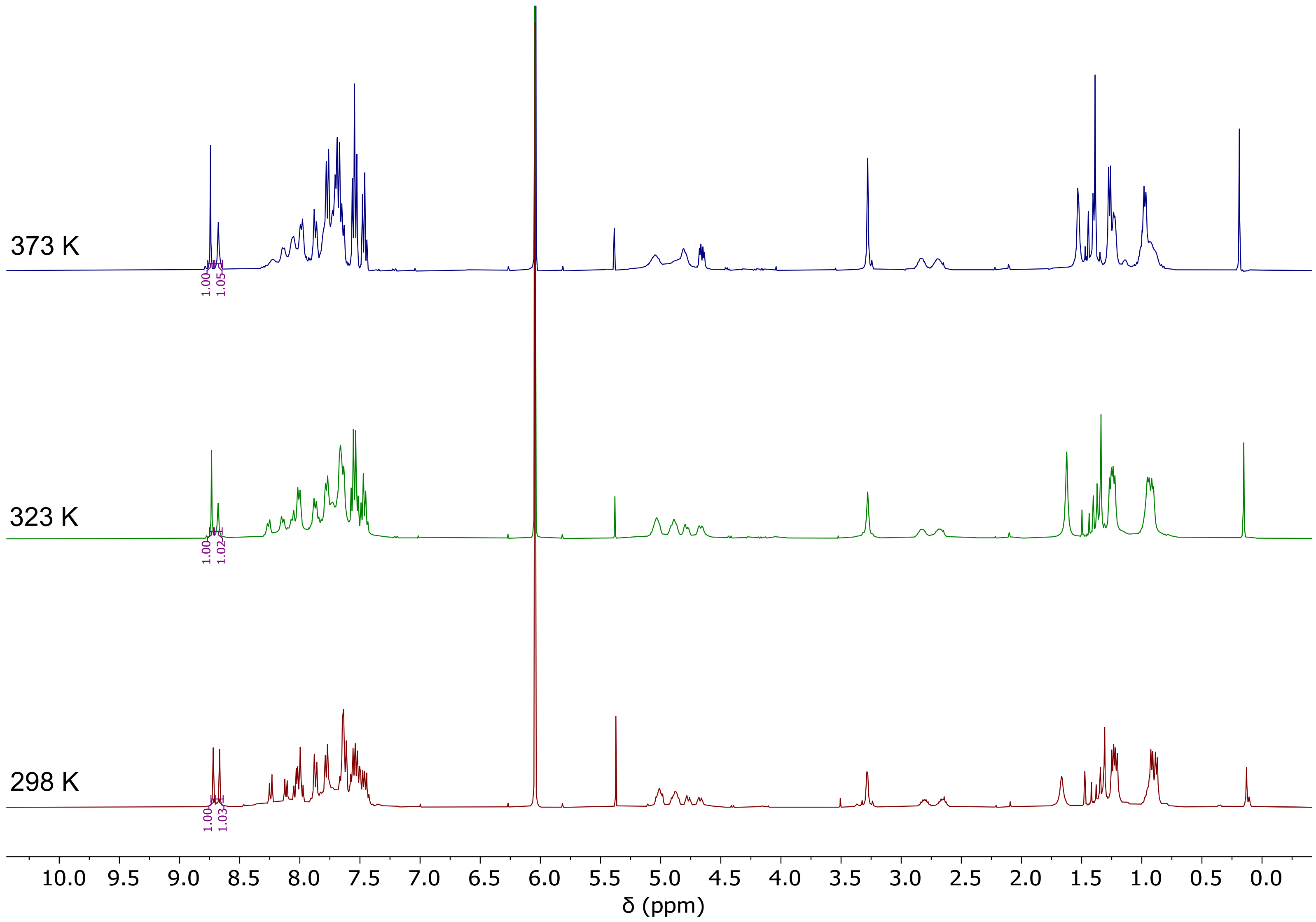

Supplementary Figure 8-5: Variable Temperature NMR of the *MP*-Isomer **8c**. The consistence of the two proton peaks at ~8.75 ppm even at high temperatures (373 K) shows conformational stability.

L-Valinol-Perylene Diimide-1,7-Dibromide 1

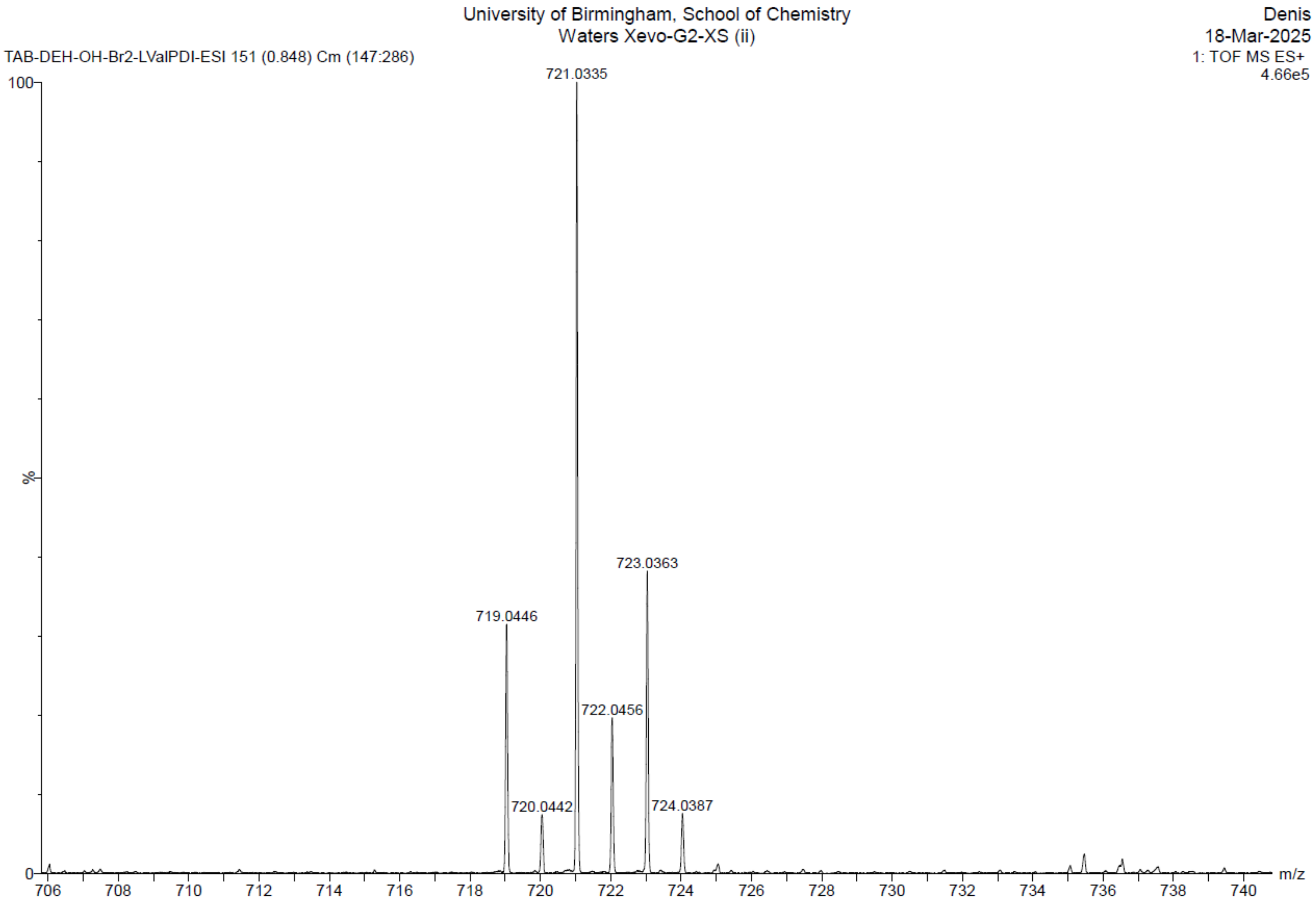

TBS-O-L-Valinol PDI Dibromide 2

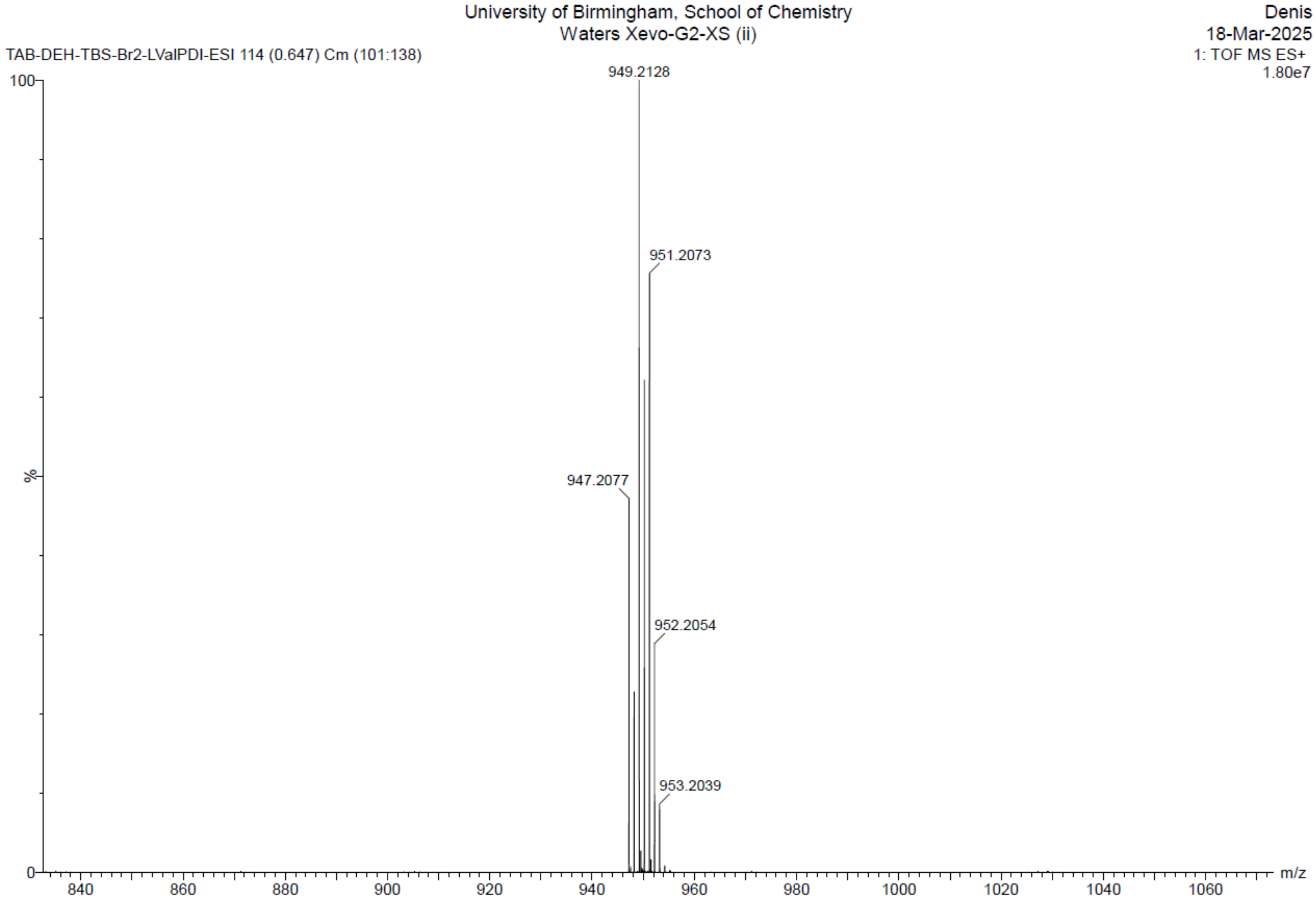

TBS-O-bis(phenyl)-L-Valinol PDI 3

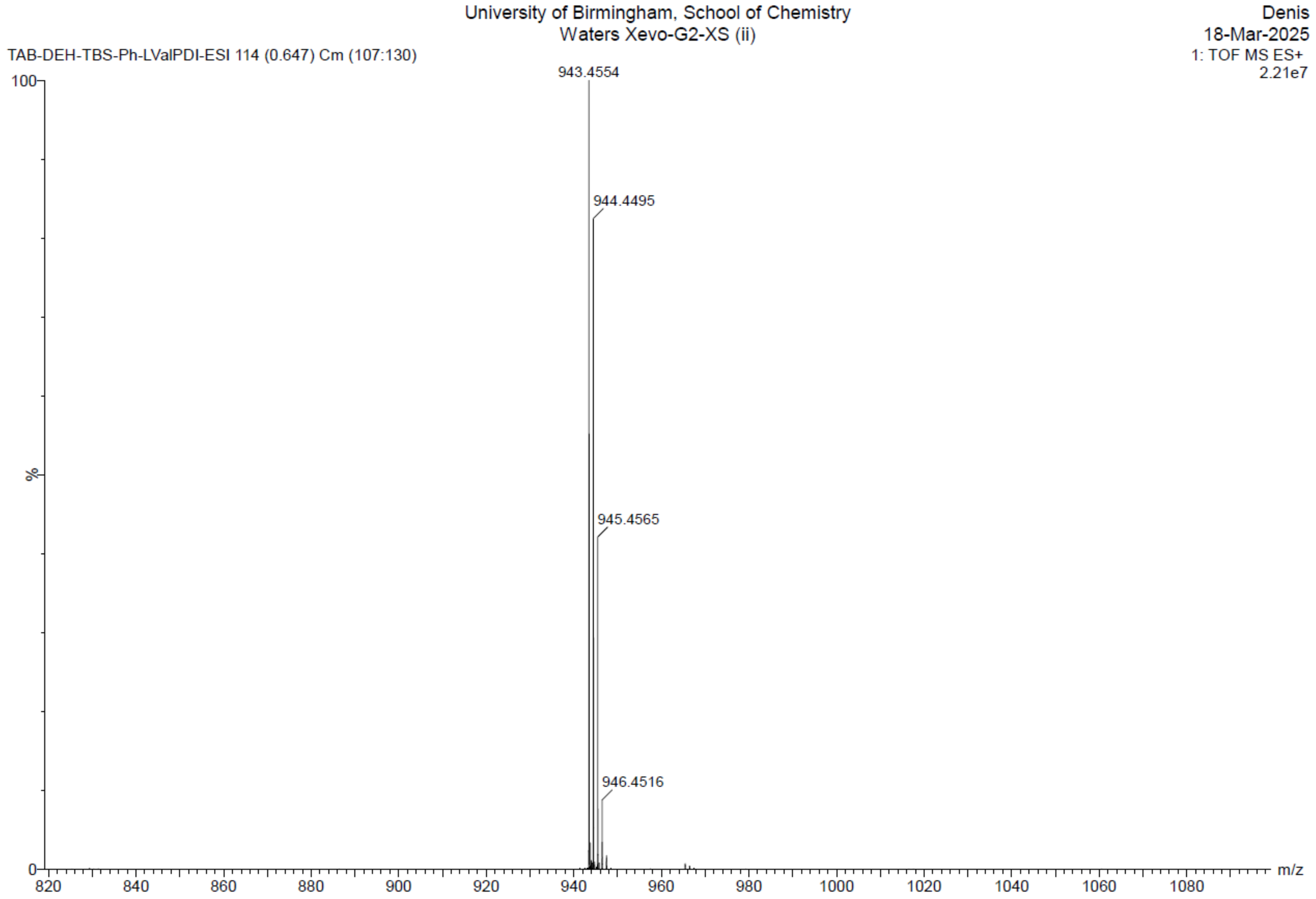

OH-bis(phenyl)-L-Valinol PDI 4

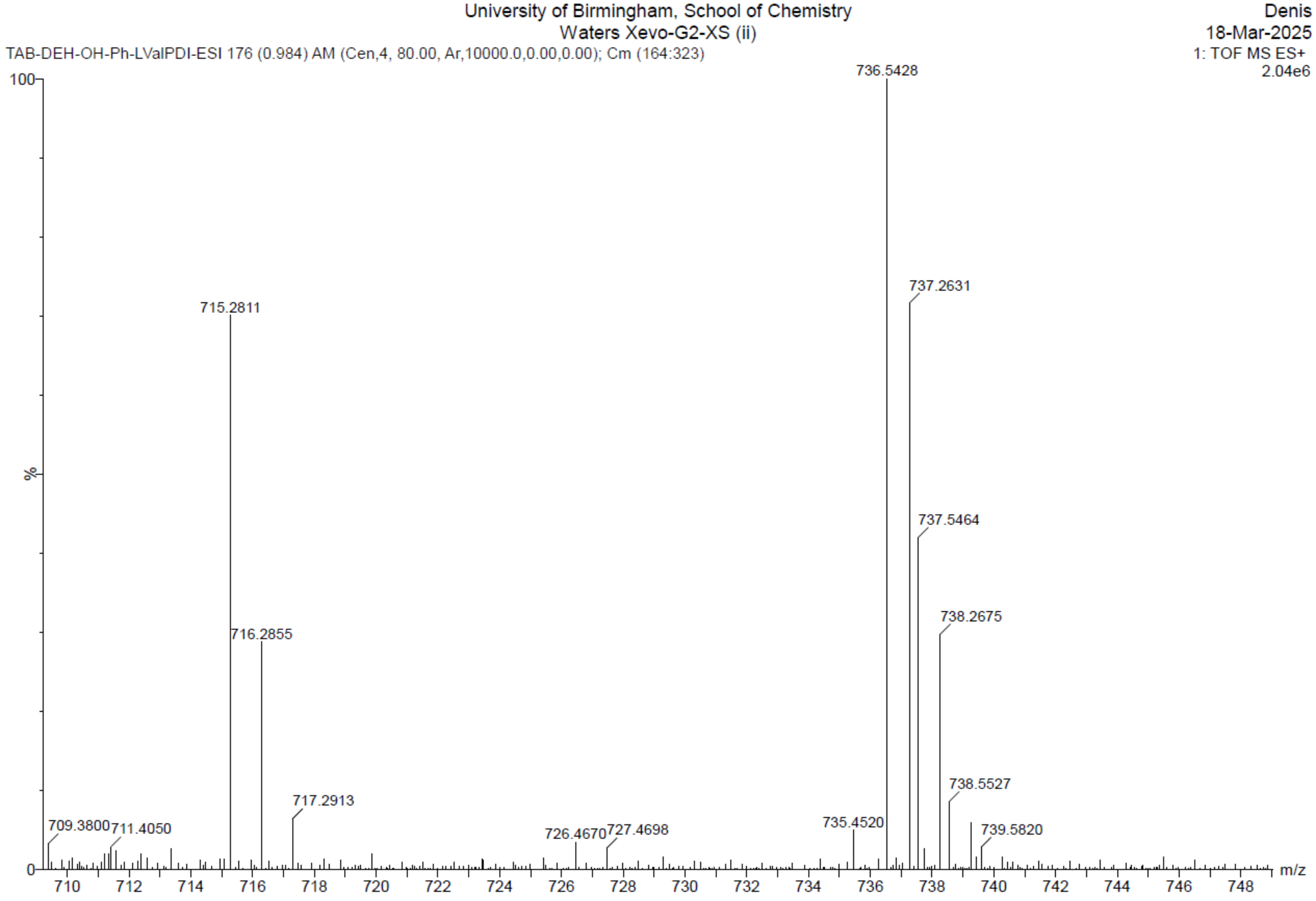

Bis(phenyl)-L-Valinol PDI Macrocycle 5

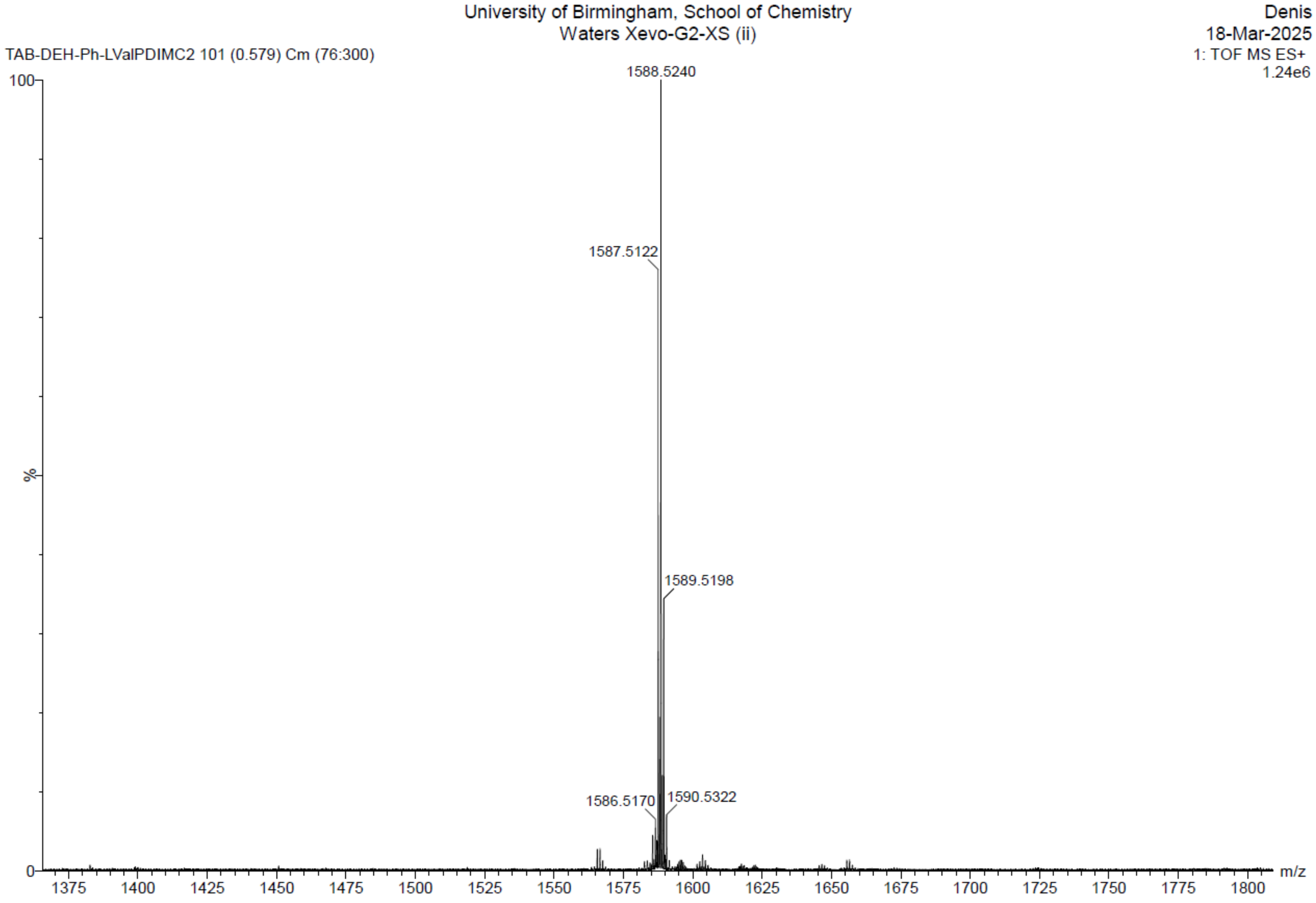

TBS-O-bis(terphenyl)-L-Valinol PDI 6

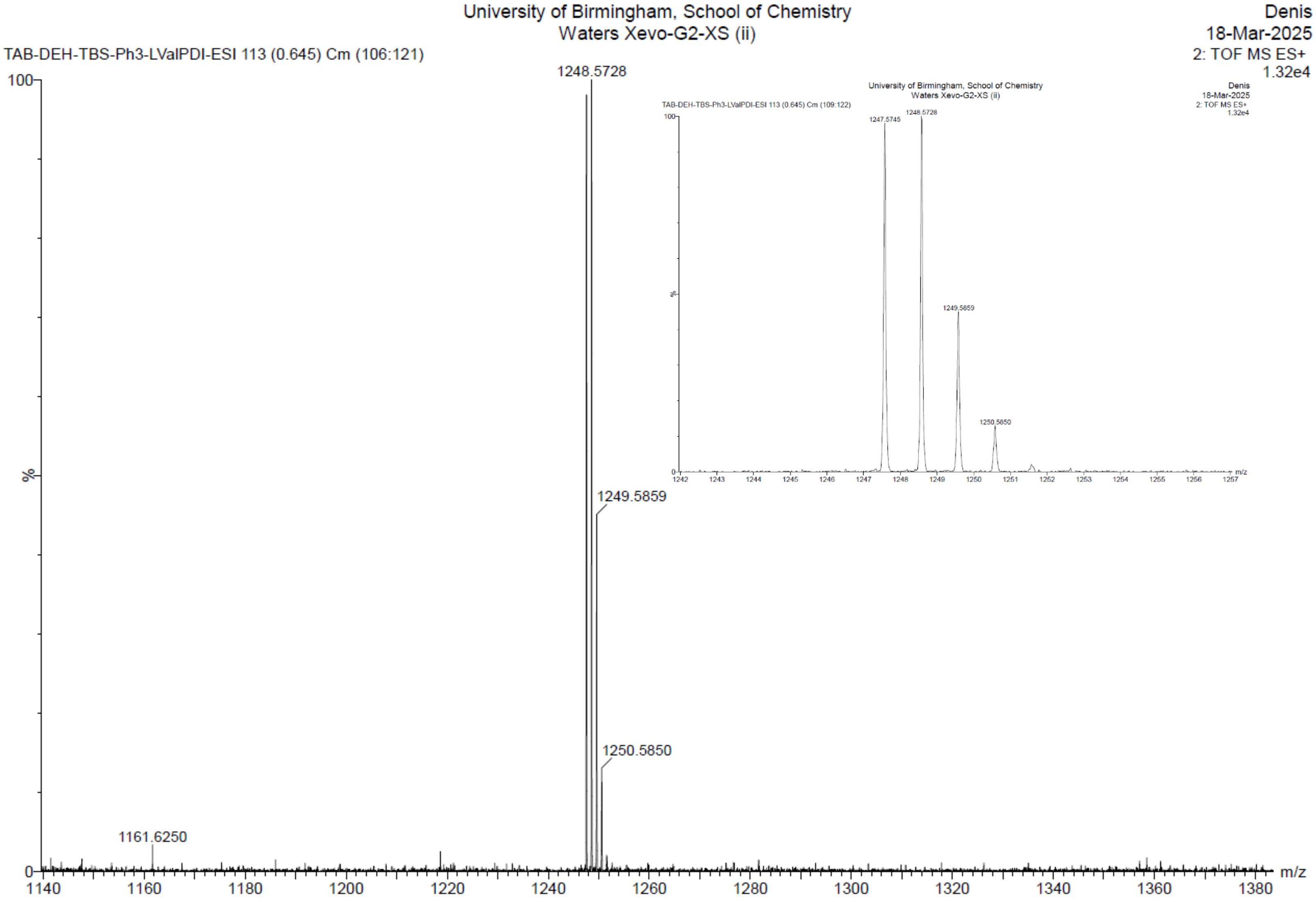

OH-bis(terphenyl)-L-Valinol PDI 7

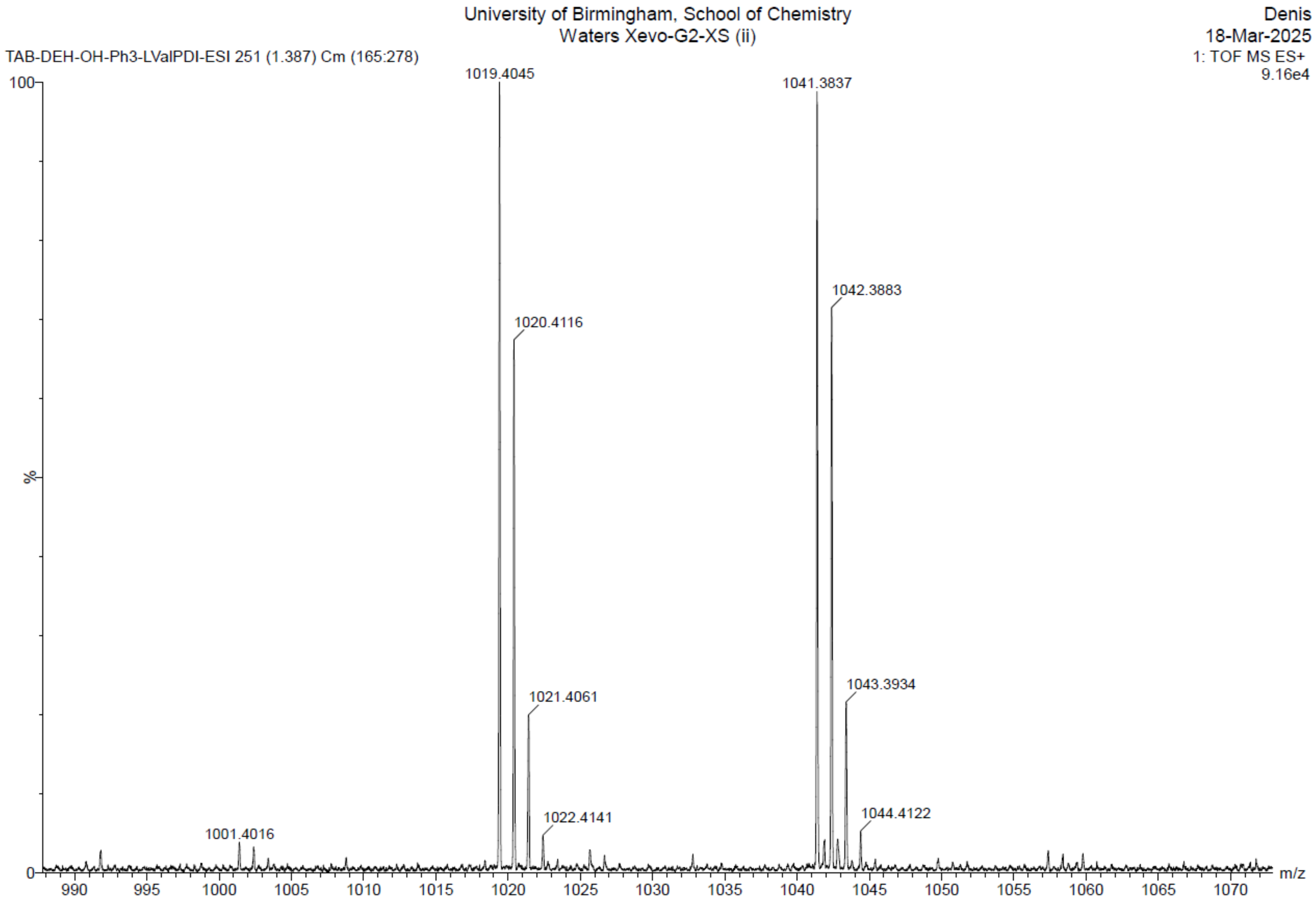

Bis(terphenyl)-L-Valinol PDI Macrocycle 8

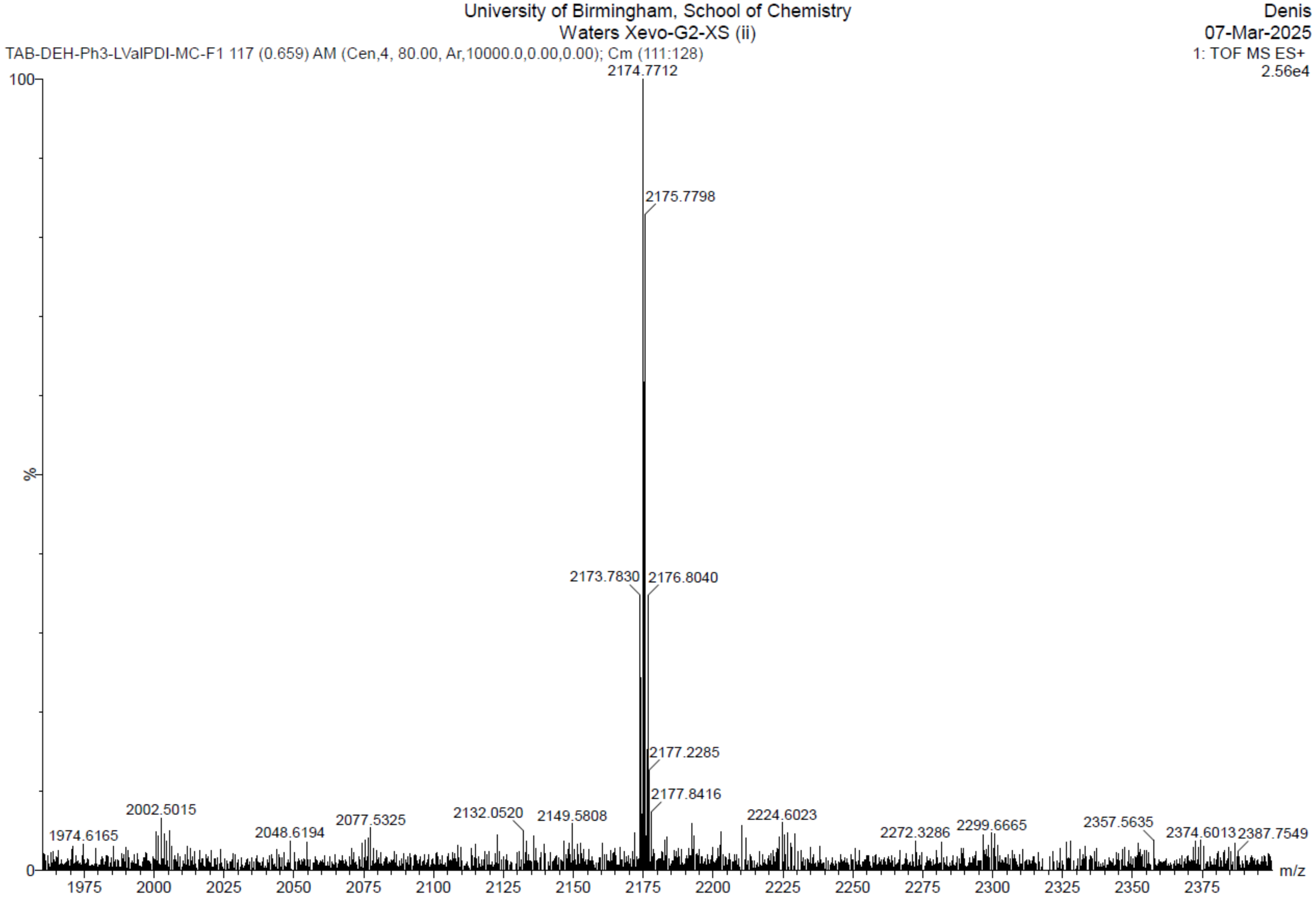

9) Supplementary References

1. Carr, R. *et al.* Induced circularly polarized luminescence arising from anion or protein binding to racemic emissive lanthanide complexes. *Methods Appl. Fluoresc.* **2**, 024007 (2014).
2. Ulatowski, F., Dąbrowa, K., Bałakier, T. & Jurczak, J. Recognizing the Limited Applicability of Job Plots in Studying Host–Guest Interactions in Supramolecular Chemistry. *J. Org. Chem.* **81**, 1746–1756 (2016).
3. Coles, S. J. *et al.* Leading Edge Chemical Crystallography Service Provision and Its Impact on Crystallographic Data Science in the Twenty-First Century. in *21st Century Challenges in Chemical Crystallography I: History and Technical Developments* (eds. Mingos, D. M. P. & Raithby, P. R.) 69–140 (Springer International Publishing, Cham, 2020). doi:10.1007/430_2020_63.
4. Sheldrick, G. M. SHELXT – Integrated space-group and crystal-structure determination. *Acta Cryst A* **71**, 3–8 (2015).
5. Sheldrick, G. M. Crystal structure refinement with SHELXL. *Acta Cryst C* **71**, 3–8 (2015).
6. Dolomanov, O. V., Bourhis, L. J., Gildea, R. J., Howard, J. a. K. & Puschmann, H. OLEX2: a complete structure solution, refinement and analysis program. *J Appl Cryst* **42**, 339–341 (2009).
7. Neese, F. Software update: The ORCA program system—Version 5.0. *WIREs Computational Molecular Science* **12**, e1606 (2022).
8. Bursch, M., Mewes, J.-M., Hansen, A. & Grimme, S. Best-Practice DFT Protocols for Basic Molecular Computational Chemistry. *Angewandte Chemie International Edition* **61**, e202205735 (2022).
9. Brandenburg, J. G., Bannwarth, C., Hansen, A. & Grimme, S. B97-3c: A revised low-cost variant of the B97-D density functional method. *The Journal of Chemical Physics* **148**, 064104 (2018).
10. Weigend, F. & Ahlrichs, R. Balanced basis sets of split valence, triple zeta valence and quadruple zeta valence quality for H to Rn: Design and assessment of accuracy. *Phys. Chem. Chem. Phys.* **7**, 3297–3305 (2005).
11. Barone, V. & Cossi, M. Quantum Calculation of Molecular Energies and Energy Gradients in Solution by a Conductor Solvent Model. *J. Phys. Chem. A* **102**, 1995–2001 (1998).
12. Goerigk, L. & Grimme, S. Efficient and Accurate Double-Hybrid-Meta-GGA Density Functionals—Evaluation with the Extended GMTKN30 Database for General Main Group Thermochemistry, Kinetics, and Noncovalent Interactions. *J. Chem. Theory Comput.* **7**, 291–309 (2011).
13. Kossmann, S. & Neese, F. Comparison of two efficient approximate Hartee–Fock approaches. *Chemical Physics Letters* **481**, 240–243 (2009).
14. Grimme, S., Ehrlich, S. & Goerigk, L. Effect of the damping function in dispersion corrected density functional theory. *Journal of Computational Chemistry* **32**, 1456–1465 (2011).
15. Pracht, P., Bohle, F. & Grimme, S. Automated exploration of the low-energy chemical space with fast quantum chemical methods. *Phys. Chem. Chem. Phys.* **22**, 7169–7192 (2020).
16. Bannwarth, C., Ehlert, S. & Grimme, S. GFN2-xTB—An Accurate and Broadly Parametrized Self-Consistent Tight-Binding Quantum Chemical Method with Multipole Electrostatics and Density-Dependent Dispersion Contributions. *J. Chem. Theory Comput.* **15**, 1652–1671 (2019).
17. Ehlert, S., Stahn, M., Spicher, S. & Grimme, S. Robust and Efficient Implicit Solvation Model for Fast Semiempirical Methods. *J. Chem. Theory Comput.* **17**, 4250–4261 (2021).
18. Chai, J.-D. & Head-Gordon, M. Systematic optimization of long-range corrected hybrid density functionals. *The Journal of Chemical Physics* **128**, 084106 (2008).
