# Supplementary material for: Chirally locked and dynamic bis-perylene diimide macrocycles with multiple sources of chirality
Source: Commun Chem. 2026 Jan 23;9:102. doi: 10.1038/s42004-026-01904-z (PMC12932710; doi:10.1038/s42004-026-01904-z)
Supplement: Supplementary file 3 — Description of Additional Supplementary Files [file 42004_2026_1904_MOESM3_ESM.pdf]

Description of Additional Supplementary Files:

File name: Supplementary Data 1

Description: DFT optimised structure of macrocycle **5** *MM* stereoisomer

File name: Supplementary Data 2

Description: DFT optimised structure of macrocycle **5** *MP* stereoisomer

File name: Supplementary Data 3

Description: DFT optimised structure of a Ph-substituted PDI monomer *M* stereoisomer

File name: Supplementary Data 4

Description: DFT optimised structure of a Ph-substituted PDI monomer *P* stereoisomer

File name: Supplementary Data 5

Description: Transition state for the interconversion between the *P* and *M* PDI monomer stereoisomers

File name: Supplementary Data 6

Description: cif for macrocycle **8c**
